# Supplementary material for: Two-year persistence of MERS-CoV-specific antibody and T cell responses after MVA-MERS-S vaccination in healthy adults
Source: Nat Commun. 2026 Jan 9;17:480. doi: 10.1038/s41467-025-68248-5 (PMC12800105; doi:10.1038/s41467-025-68248-5)
Supplement: Supplementary file 1 — Supplementary Information [file 41467_2025_68248_MOESM1_ESM.pdf]

Supplementary Information

Two-year persistence of MERS-CoV-specific antibody and T cell responses after MVA-MERS-S vaccination in healthy adults

Leonie Mayer[1,2,3*], Anahita Fathi[1,2,3,4], Hanna-Marie Weichel[1,2,3], Matthijs P. Raadsen[5], Christine Dahlke[1,2,3], Anna Mykytyn[5], Jordi Rodon[6], Gesche K. Gerresheim[7,8], Merel R. te Marvelde[5], Leonie M. Weskamm[1,2,3], Ilka Grewe[1,2,3], Claudia Schlesner[1,2,3], Marc Lütgehetmann[3,9], Christian Drosten[6,10], Stephan Becker[7,8], Bart L. Haagmans[5], Svenja Hardtke[1,2,3], Marylyn M. Addo[1,2,3*]

[1] Institute for Infection Research and Vaccine Development (IIRVD), University Medical Centre Hamburg-Eppendorf, Hamburg, Germany

[2] Department for Clinical Immunology of Infectious Diseases, Bernhard Nocht Institute for Tropical Medicine, Hamburg, Germany

[3] German Centre for Infection Research, partner site Hamburg-Lübeck-Borstel-Riems, Hamburg, Germany

[4] University Medical Centre Hamburg-Eppendorf, First Department of Medicine, Division of Infectious Diseases, Hamburg, Germany

[5] Department of Viroscience, Erasmus Medical Centre, Rotterdam, The Netherlands

[6] Institute of Virology, Charité - Universitätsmedizin Berlin, corporate member of Freie Universität Berlin, Humboldt-Universität zu Berlin and Berlin Institute of Health, Berlin, Germany

[7] Institute of Virology, Philipps University Marburg, Marburg, Germany

[8] German Centre for Infection Research, partner site Gießen-Marburg-Langen, Marburg, Germany

[9] Institute of Medical Microbiology, Virology and Hygiene, University Medical Centre Hamburg-Eppendorf, Hamburg, Germany

[10] German Centre for Infection Research, associated partner Charité, Berlin, Germany

*Correspondence: Leonie Mayer (l.mayer@uke.de), Marylyn M. Addo (m.addo@uke.de)

Supplementary Tables

Supplementary Table 1: Demographics of the safety set. Summary of the demographic characteristics of all participants of the long-term follow-up study, stratified by study group. Data were collected at the screening visit before V1. V = vaccination, M = month, SD = standard deviation, PFU = plaque-forming units.

	28-day 10^7 PFU (*n* = 11)	56-day 10^7 PFU (*n* = 13)	28-day 10^8 PFU (*n* = 14)	56-day 10^8 PFU (*n* = 12)	Placebo (*n* = 5)	Total (*n* = 55)
Sex						
Male – n (%)	7 (64%)	7 (54%)	6 (43%)	6 (50%)	1 (20%)	27 (49%)
Female – n (%)	4 (36%)	6 (46%)	8 (57%)	6 (50%)	4 (80%)	28 (51%)
Age – years						
mean (SD)	30 (±12.7)	36 (±11.6)	35 (±12.1)	34 (±10.8)	41 (±8.1)	35 (±11.5)
Body-mass index						
mean (SD)	24 (±3.5)	24 (±2.6)	23 (±2.5)	23 (±2.3)	22 (±1.8)	23 (±2.6)

Supplementary Table 2: Targeted illness and concomitant medication. Participants included in the safety set (*n* = 55) reporting COVID-19 or any febrile illness, having received COVID-19 or any other vaccination (within 4 weeks of visit), and having received immunosuppressive therapy (defined as >14 days treatment with immune suppressants or other immune-modifying drugs) between timepoints V3M1-V3M6, V3M6-V3M12, or V3M12-V3M24 of the two-year follow-up. V = vaccination, M = month.

Indication	V3M1-V3M6	V3M6-V3M12	V3M12-V3M24
Documented COVID-19 – *n* (%)	14 (25%)	4 (7%)	8* (15%)
Any febrile illness – *n* (%)	1 (2%)	1 (2%)	3 (5%)
COVID-19 vaccination⁺ – *n* (%)	5 (9%)	2 (4%)	4 (7%)
Other vaccinations# – *n* (%)	2 (4%)	3 (5%)	0
Immunosuppressive therapy‡ – *n* (%)	0	1 (2%)	1 (2%)

*One participant reported two COVID-19 episodes during this time period.
⁺ Two participants reported two COVID-19 vaccinations during the long-term follow-up.
# Reported vaccines: FSME-Immun®, Encepur®, Shingrix®, Ixiaro®.
‡ Immunosuppressive treatment: Prednisolone (oral), Budesonide/Formoterol (inhalation).

Supplementary Table 3: Serious adverse events. Newly reported serious adverse events (SAE) of the safety set during the two-year follow-up period. mITT = modified intention-to-treat, PFU = plaque-forming units.

Participant	Adverse event (reported term)	Adverse event (preferred term)	System organ class (SOC)	Severity	Relatedness	Days since last study vaccination	Study group
1	Tibia fracture left	Tibia fracture	Injury, poisoning and procedural complications	Severe (Grade 3)	Not related	359	28-day, 10^8 PFU[#]
	Symptomatic Cholecystolithiasis *	Cholelithiasis	Hepatobiliary disorders	Severe (Grade 3)	Not related	927	
	Cholecystectomy *	Cholecystectomy	Surgical and medical procedures	Moderate (Grade 2)	Not related	947	
2	Cruciate ligament rupture left	Ligament rupture	Musculoskeletal and connective tissue disorders	Severe (Grade 3)	Not related	644	Placebo[‡]
3	Stillbirth (female partner)	Stillbirth	Pregnancy, puerperium and perinatal conditions	Severe (Grade 3)	Not related	635	56-day, 10^7 PFU[‡]
4	Surgery for inguinal hernia left	Inguinal hernia	Gastrointestinal disorders	Moderate (Grade 2)	Not related	628	56-day, 10^8 PFU[#]

* These SAEs are timely and casually related.
[#] Safety cohort.
[‡] Immunogenicity cohort.

Supplementary Table 4: Targeted exposure. Two participants (included in the immunogenicity set) reported stays in countries where MERS-CoV is endemic during the long-term follow-up period. PFU = plaque-forming units.

Participant	Country of stay	Duration of stay (days)	Study group
1	Saudi Arabia	1	28-day, 10^8 PFU
	United Arab Emirates	7	
	Jordan	1	
	Qatar	1	
2	United Arab Emirates	1	56-day, 10^7 PFU

Supplementary Table 5: Geometric mean titers (GMTs) of S1 IgG by study group. Antibody responses were measured before first vaccination (V1), before second vaccination (V2), after second vaccination at months 1 (V2M1), 3 (V2M3), and 6 (V2M6) as well as after third vaccination at months 1 (V3M1), 6 (V3M6), 12 (V3M12) and 24 (V3M24).

	28-day 10^7 PFU		56-day 10^7 PFU		28-day 10^8 PFU		56-day 10^8 PFU		Placebo	
Timepoint	*n*	GMT [IU/ml] (95% CI)	*n*	GMT [IU/ml] (95% CI)	*n*	GMT [IU/ml] (95% CI)	*n*	GMT [IU/ml] (95% CI)	*n*	GMT [IU/ml] (95% CI)
baseline	11	6 (4.5-8.5)	12	5 (4.3-5.6)	12	6 (4.5-8.4)	8	8 (3.8-16.1)	5	5 (4.2-5.7)
V1M1-2	11	7 (4.2-13.0)	12	7 (4.9-9.7)	12	8 (5.4-11.5)	8	9 (4.4-17.8)	5	5 (4.6-4.6)
V2M1	10	48 (18.1-126.0)	12	27 (12.5-60.3)	11	57 (35.5-92.5)	7	48 (23.8-98.5)	4	5 (4.6-4.6)
V2M4-5	11	8 (3.6-19.5)	12	16 (8.0-33.3)	11	7 (4.4-12.0)	7	20 (6.5-59.3)	5	5 (3.9-6.6)
V2M6	11	8 (3.9-15.7)	12	13 (6.6-25.2)	12	6 (4.1-10.1)	8	19 (6.7-51.7)	5	5 (4.6-4.6)
V3M1	11	166 (58.2-470.5)	12	125 (37.9-412.2)	12	163 (96.2-274.7)	8	190 (77.5-463.7)	5	5 (4.6-4.6)
V3M6	11	69 (22.1-212.6)	12	44 (17.1-111.3)	11	85 (44.3-162.0)	8	89 (42.4-186.4)	5	5 (4.6-4.6)
V3M12	11	49 (16.5-146.8)	11	28 (10.7-74.2)	11	52 (24.0-112.5)	8	72 (34.8-149.3)	4	5 (4.6-4.6)
V3M24	9	43 (12.2-153.7)	11	19 (8.3-45.0)	12	44 (24.5-79.9)	8	41 (20.2-83.6)	4	5 (4.4-5.0)

Supplementary Table 6: S1 IgG seropositivity rates by study group at timepoints after V3. Participants are defined as being seropositive if the titer at the specific timepoint after vaccination is at least 4-fold above the individuals baseline titer before V1.

	28-day 10^7 PFU	56-day 10^7 PFU	28-day 10^8 PFU	56-day 10^8 PFU	Total
Timepoint	*n* (%)	*n* (%)	*n* (%)	*n* (%)	*n* (%)
V3M1	10/11 (91%)	9/12 (75%)	11/12 (92%)	7/8 (88%)	37/43 (86%)
V3M6	9/11 (82%)	8/12 (67%)	10/11 (91%)	6/8 (75%)	33/42 (79%)
V3M12	7/11 (64%)	6/11 (55%)	9/11 (82%)	5/8 (63%)	27/41 (64%)
V3M24	5/9 (56%)	4/11 (36%)	10/12 (83%)	5/8 (63%)	24/40 (60%)

Supplementary Table 7: Demographics of immunogenicity set by vaccine response. Summary of the demographic characteristics of participants of the long-term follow-up that met the modified intention-to-treat definition, stratified by the S1 IgG response at V3M1 into low and high responders. Data were collected at the screening visit before V1. V = vaccination, M = month, SD = standard deviation, PFU = plaque-forming units.

	Low responders (*n* = 22)	High responders (*n* = 21)
Sex		
Male – *n* (%)	9 (50%)	13 (52%)
Female – *n* (%)	13 (50%)	8 (48%)
Age – years		
mean (SD)	34 (±11.5)	35 (±11.4)
Body-mass index		
mean (SD)	24 (±2.6)	23 (±2.6)

Supplementary Figures

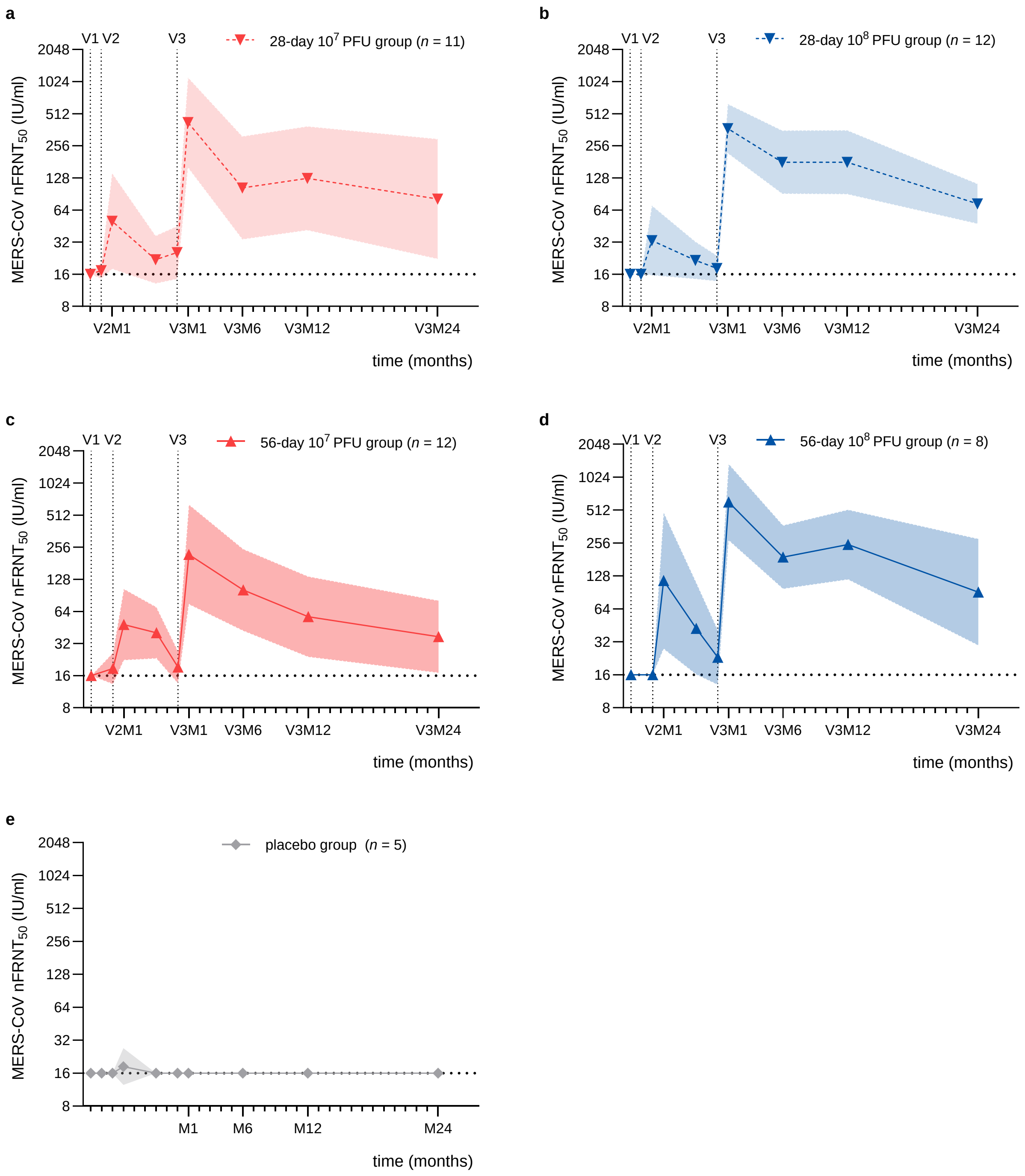

Supplementary Fig. 1: Longitudinal pseudovirus neutralizing antibody responses elicited by MVA-MERS-S vaccination. Longitudinal neutralizing responses are shown as geometric mean titers with 95% confidence interval bands for the 28-day 10^7 PFU group (a), 28-day 10^8 PFU group (b), 56-day 10^7 PFU group (c), 56-day 10^8 PFU group (d) and placebo group (e). Neutralizing titers were calibrated to the WHO standard (IU/ml, y-axis) and shown as a function of time since last vaccination (months, x-axis). The dotted horizontal line shows the assay-specific cut-off (16 IU/ml). The dotted vertical lines show the timepoints of vaccinations V1, V2, and V3. V = vaccination, M = month, PFU = plaque-forming units, IU = international units, $nFRNT_{50}$ = normalized pseudovirus 50% focus reduction neutralization assay. Source data are provided as a Source Data file.

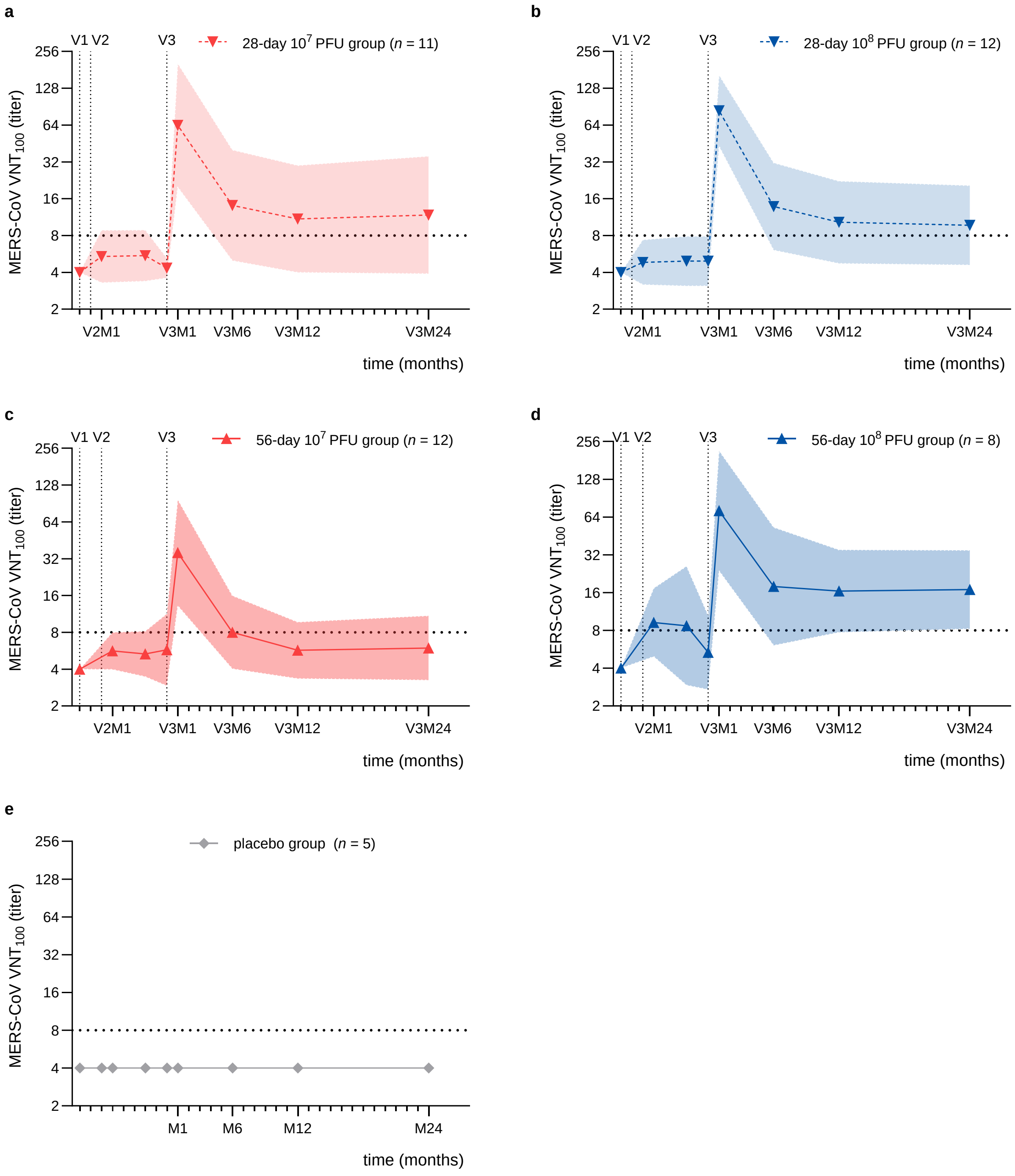

Supplementary Fig. 2: Longitudinal live-virus neutralizing antibody responses elicited by MVA-MERS-S vaccination. Longitudinal neutralizing responses are shown as geometric mean titers with 95% confidence interval bands for the 28-day 10^7 PFU group (a), 28-day 10^8 PFU group (b), 56-day 10^7 PFU group (c), 56-day 10^8 PFU group (d) and placebo group (e). Neutralizing responses (titers, y-axis) are shown as a function of time since last vaccination (months, x-axis). The dotted horizonal line indicate the lower limit of detection (titer = 8). Samples without neutralization were set to 4. The dotted vertical lines show the timepoints of vaccinations V1, V2, and V3. V = vaccination, M = month, PFU = plaque-forming units, VNT_{100} = live-virus neutralization test. Source data are provided as a Source Data file.

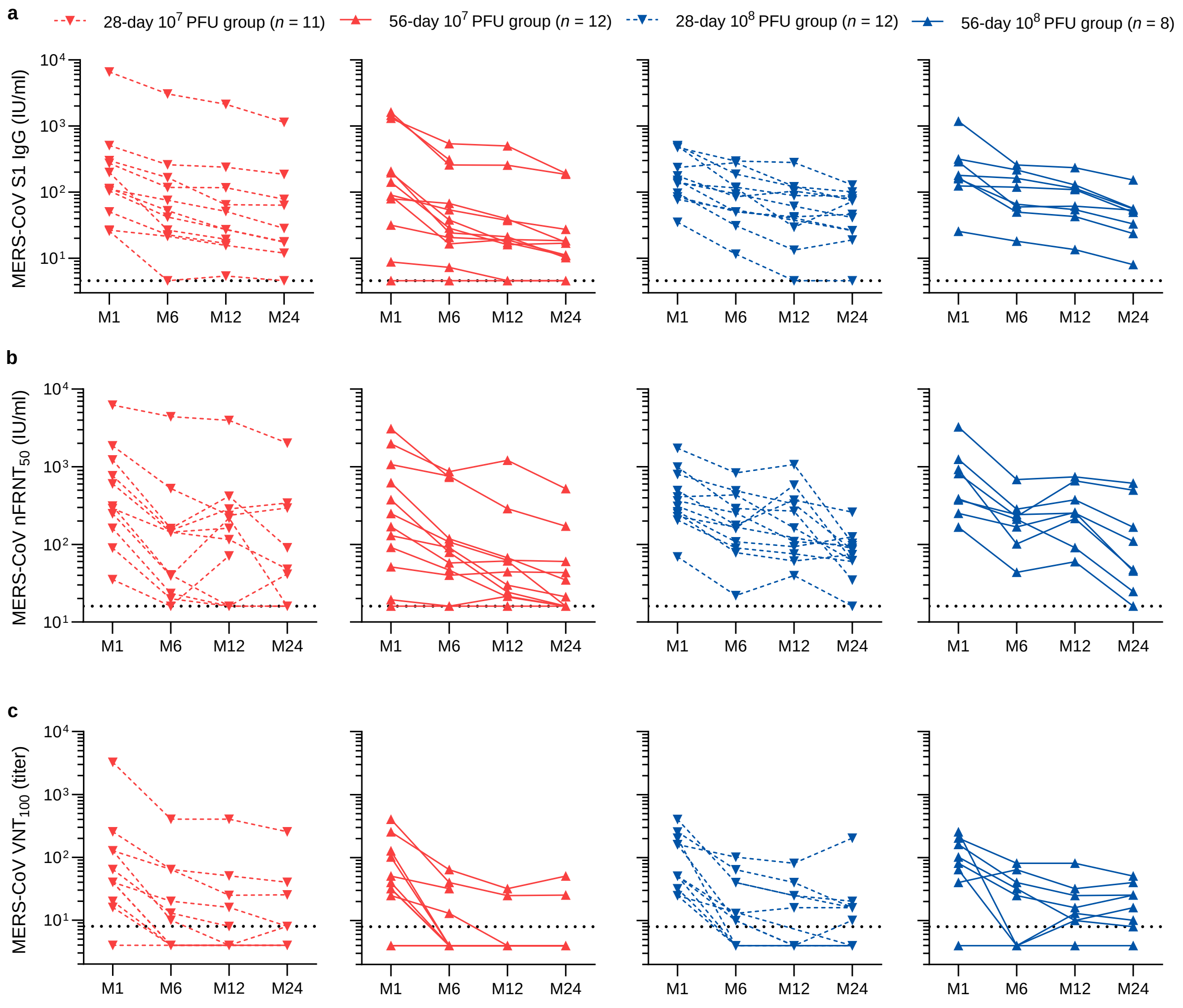

Supplementary Fig. 3: Antibody responses by individual at long-term follow-up timepoints. Antibody responses were measured by S1 IgG ELISA (a), nFRNT$_{50}$ pseudovirus neutralization assay (b) and VNT$_{100}$ live-virus neutralization assay (c). Lines connect data points of individual participants. The dotted horizontal lines indicate the assay-specific lower limit of detection. M = month, PFU = plaque-forming units, IU = international units, nFRNT$_{50}$ = normalized pseudovirus 50% focus reduction neutralization assay, VNT$_{100}$ = live-virus neutralization test. Source data are provided as a Source Data file.

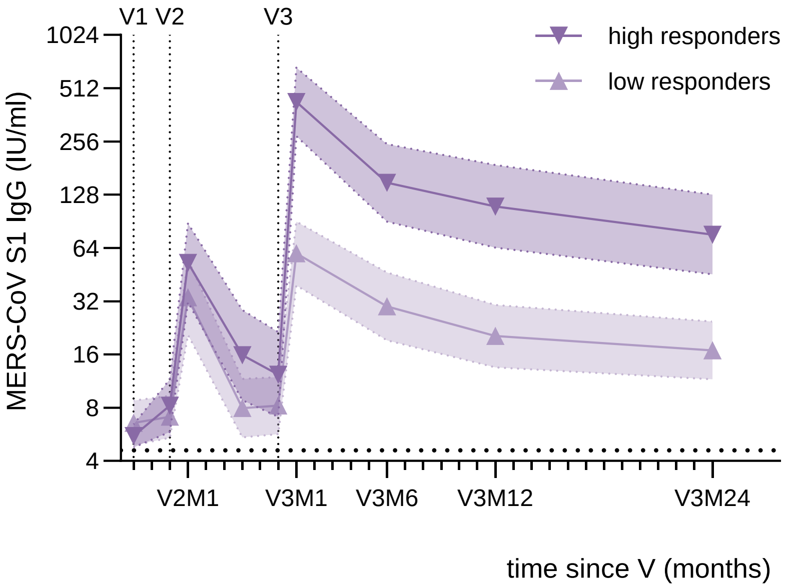

Supplementary Fig. 4: Longitudinal S1 IgG responses of high and low responders. Longitudinal S1 IgG responses are shown as geometric mean titers with 95% confidence interval bands. Participants were divided into high responders (dark purple, *n* = 21), defined as having V3M1 S1 IgG titers above the geometric mean titer of all vaccinated individuals and low responders (light purple, *n* = 22), defined as having V3M1 S1 IgG titers below the geometric mean titer of all vaccinated individuals. S1 IgG titers were calibrated to the WHO standard (IU/ml, y-axis) and shown as a function of time since last vaccination (months, x-axis). The dotted horizontal lines indicate the lower limit of detection (4.6 IU/ml). The dotted vertical lines show the timepoints of vaccinations V1, V2, and V3. V = vaccination, M = month, S = spike, IU = international units. Source data are provided as a Source Data file.

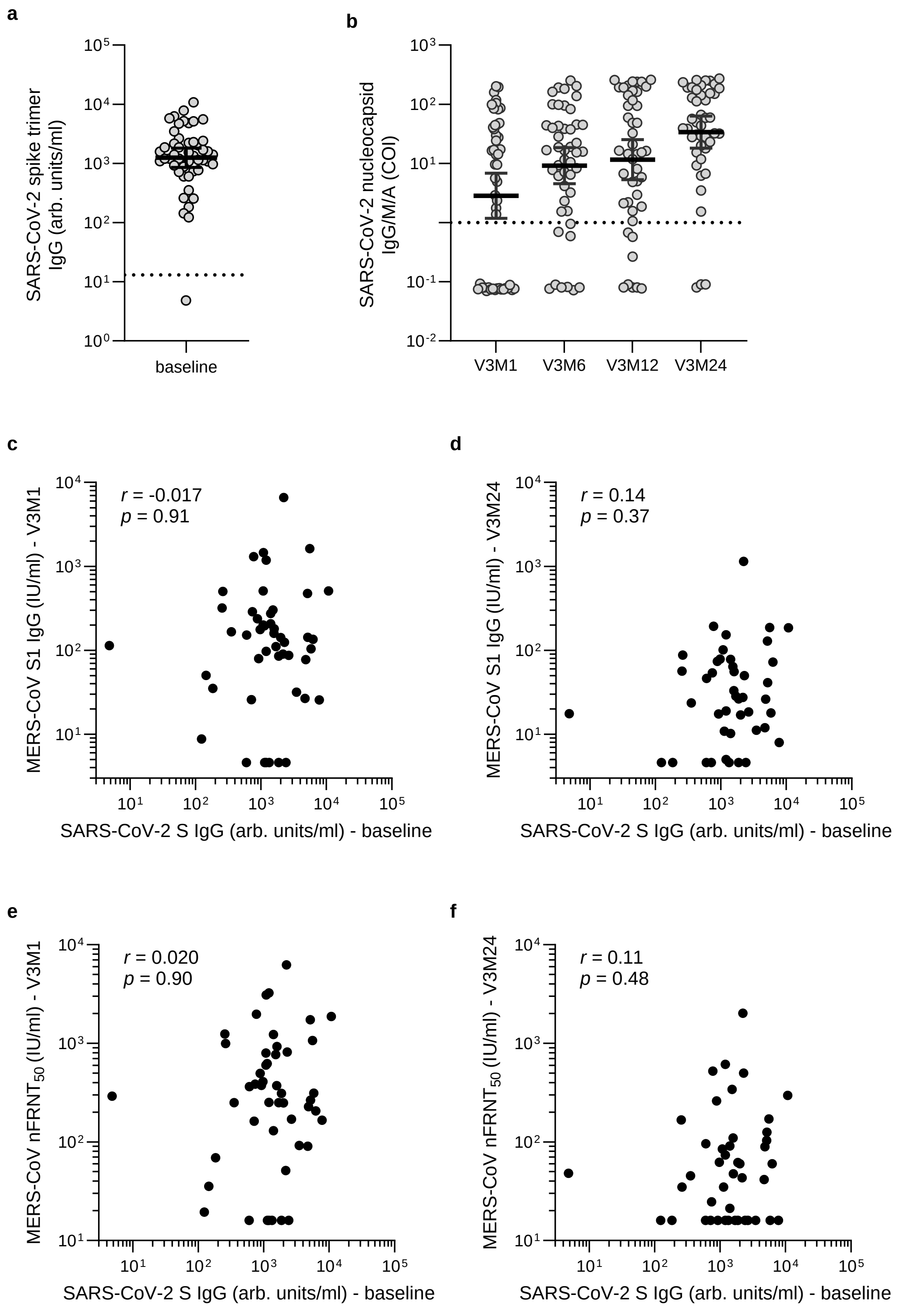

Supplementary Fig. 5: SARS-CoV-2-specific antibody response. a) Anti-spike IgG at baseline before MVA-MERS-S vaccination (*n* = 48). b) Anti-nucleocapsid IgG/M/A at the long-term follow-up timepoints (*n* = 48). Correlation between SARS-CoV-2 spike (S)-specific IgG at baseline and (c) MERS-CoV S1 IgG at V3M1 (*n* = 48) and (d) MERS-CoV S1 IgG at V3M24 (*n* = 44), as well as (e) MERS-CoV neutralizing titers at V3M1 (*n* = 48) and (f) MERS-CoV neutralizing titers at V3M24 (*n* = 44). COI = cut-off index, arb. units = arbitrary units, IU = international units, $nFRNT_{50}$ = normalized pseudovirus 50% focus reduction neutralization assay. Source data are provided as a Source Data file.

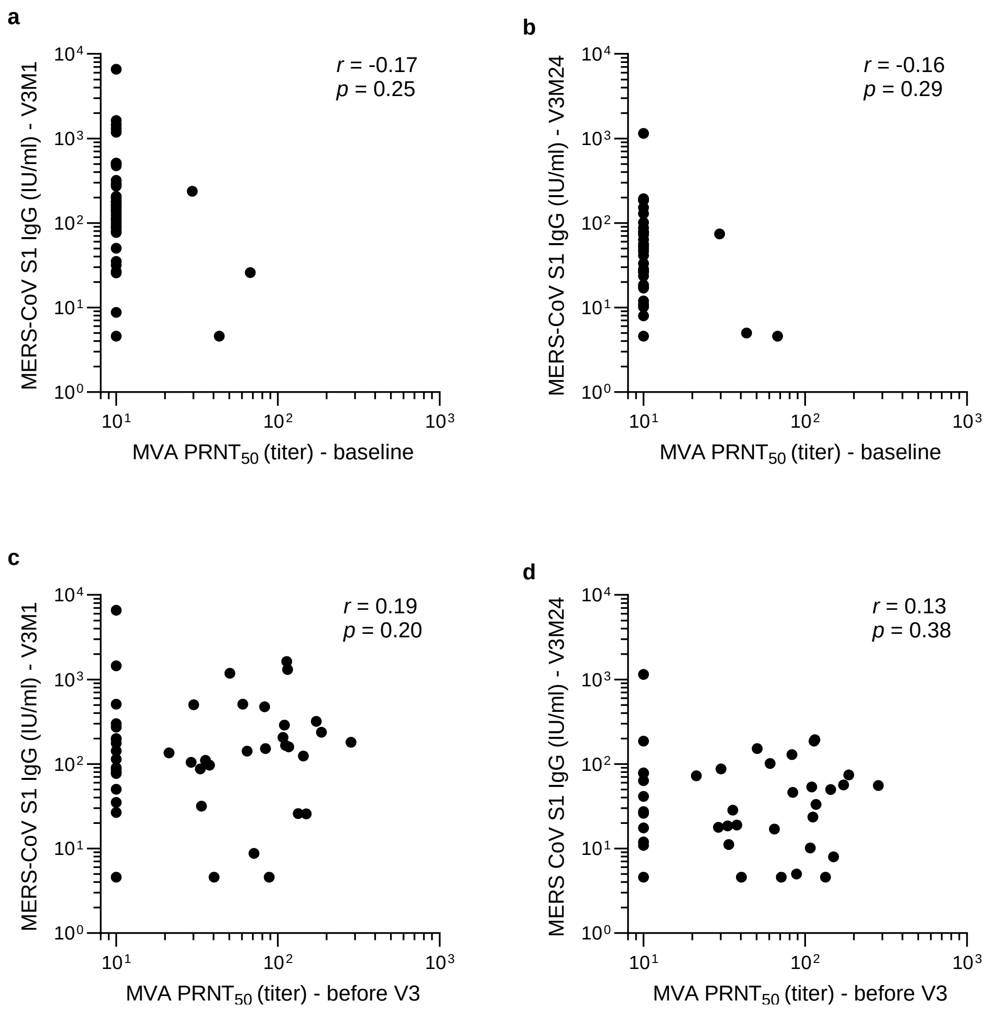

Supplementary Fig. 6: Anti-vector immunity. Correlation of baseline MVA-neutralizing antibody titers at baseline and (a) MERS-CoV S1 IgG at V3M1 and (b) at V3M24 (n = 48). Correlation of MVA-neutralizing antibody titers before V3 and (c) MERS-CoV S1 IgG at V3M1 and (d) at V3M24. V = vaccination, M = month, $FRNT_{50}$ = pseudovirus 50% focus reduction neutralization assay, IU = international units. Source data are provided as a Source Data file.

MVA-MERS-S_DF1 study group

Maher Almahfoud[1,2,3,4], Monika Friedrich[1,2,3], Cordula Grüttner[1,2,3], My Linh Ly[1,2,3], Stephanie Petereit[1,2,3], Tamara Zoran[1,2,3], Dominik Hillesheim[1,2], Alina Grefe[1,2], Asisa Volz[11,12], Verena Krähling[7,8], Eric van Gorp[5]

[1]Institute for Infection Research and Vaccine Development (IIRVD), University Medical Centre Hamburg-Eppendorf, Hamburg, Germany, [2]Department for Clinical Immunology of Infectious Diseases, Bernhard Nocht Institute for Tropical Medicine, Hamburg, Germany, [3]German Centre for Infection Research, partner site Hamburg-Lübeck-Borstel-Riems, Hamburg, Germany, [4]University Medical Centre Hamburg-Eppendorf, First Department of Medicine, Division of Infectious Diseases, Hamburg, Germany, [5]Department of Viroscience, Erasmus Medical Centre, Rotterdam, The Netherlands, [7]Institute of Virology, Philipps University Marburg, Marburg, Germany, [8]German Centre for Infection Research, partner site Gießen-Marburg-Langen, Marburg, Germany, [11]Institute of Virology, University of Veterinary Medicine Hannover, Hanover, Germany, [12]German Centre for Infection Research, partner site Hannover-Braunschweig, Hannover, Germany

STUDY PROTOCOL

A two-center, randomized, double-blind, placebo-controlled, phase Ib study to assess the safety, tolerability and immunogenicity of two ascending doses of the candidate vaccine MVA-MERS-S_DF-1 in healthy study subjects	
EudraCT No.	2019-000715-83
Protocol No.	CEPI-MVA-MERS-S-Phase1b
Registration number	NCT04119440
Version/Date	5.0 / 24-NOV-2022
Sponsor	University Medical Center Hamburg-Eppendorf<br>Martinistr. 52<br>20246 Hamburg, Germany
Coordinating Investi-gator	Prof. Marylyn M. Addo, MD, PhD, MSc, DTM&H<br>University Medical Center Hamburg-Eppendorf<br>I. Department of Medicine<br>Martinistr. 52<br>20246 Hamburg, Germany<br><br>Telephone: [redacted]<br>Fax: [redacted]<br>Email: [redacted]

CONFIDENTIALITY STATEMENT
The information provided in the following document is confidential and is only available for review to Principal Investigators, the Ethics Committee and the Competent Authorities. No disclosure should take place without the written authorization from the Sponsor, except to the extent necessary to obtain informed consent from potential subjects or to obtain approval of this protocol by an Ethics Committee or Regulatory Authorities.

SIGNATURES

This protocol has been approved by University Medical Center Hamburg-Eppendorf.

Sponsor Signature

Sponsor Signature	Date
Sponsor Name and function	

Coordinating Investigator

I hereby confirm that I have acknowledged the protocol and agree to conduct the study in compliance with the protocol.

Coordinating Investigator Signature	Date
Coordinating Investigator Name	

Statistician Signature

Statistician Signature	Date
Statistician Name	

**Summary of changes since last version of protocol (protocol version 1.0 to version 2.0)**		
**Amendment Number**	**Date of Amendment**	**Section Affected by Change**
01	24FEB2021	2 Synopsis
Brief description of change:<br>The synopsis was updated according to the information given in the main body.		
01	24FEB2021	8.3 Exploratory Objectives/ Study Schedule Part B
Brief description of change:<br>As given in the study schedule, footnote h, samples for exploratory objectives can be collected optionally. The decision is made by the site-specific principal investigator. Therefore, only a subset of patients will be included in the analysis of exploratory endpoints. This specification was added in section 8.3 Exploratory Objectives.<br>(according to clarification to protocol no. 01)		
01	24FEB2021	9.1 Overall Study Design and Plan-Description
Brief description of change:<br>Adaption of dose specification for a vaccination of 0.5 ml. The dose per injection is as follows:<br>Low Dose: $1x10^7 \pm 0.5$ log pfu<br>High Dose: $1x10^8 \pm 0.5$ log pfu<br>(according to clarification to protocol no. 01)		
01	24FEB2021	9.4.2 Identity of Investigational Medicinal Product(s)
A description of the placebo product was added.		
01	24FEB2021	9.6.2.2.13.1 Definition of Adverse Events, Period of Observation, Recording of Adverse Events
Correction of inconsistency in the list of AEs classified as solicited.<br>(according to clarification to protocol no. 01)		
01	24FEB2021	9.8.2 Determination of Sample Size
The planned interim analysis will be performed when all subjects completed 3 months (day 56 plus 28 days) in the study to determine optimal dose and injection scheme for future studies.<br>(according to clarification to protocol no. 01)		
01	24FEB2021	Study Schedule, 9.6.2.2.11 Reactogenicity
Subjects are asked to maintain a diary to record daily temperature and injection site and systemic reactions for 7 days after each dosing and to record medical events for 28 days after each dosing. It is planned use to an electronic diary. In case subjects cannot access the electronic diary due to technical limitations they may be provided with a paper diary.<br>(according to clarification to protocol no. 01)		
01	24FEB2021	9.10.1. Local Safety Board (LSB), 9.4.1 Treatments Administered
Correction of description of the LSB, which will not be a subunit of the DSMB. Only the summarized safety report of each LSB Meeting will be submitted to the whole DSMB for notification.<br>(according to clarification to protocol no. 01)		

**Summary of changes since last version of protocol (protocol version 2.0 to version 3.0)**		
**Amendment Number**	**Date of Amendment**	**Section Affected by Change**
02	09DEC2021	2 Synopsis
Brief description of change:<br>The synopsis was updated according to the information given in the main body.		
02	09DEC2021	Study Schedule

Brief description of change:<br>Footnote “o” added to v5, v8, v13: If the vaccination was not administered on the previous vaccination visit but all other events were performed according to protocol the visit one day after vaccination can be skipped.		
02	09DEC2021	6.0 Investigators and study administrative structure
Brief description of change:<br>Performance of safety laboratory events updated.		
02	09DEC2021	9.4.7.3 Screening, Confinement and Regular Visits/ 9.6.2.2.8 Clinical Laboratory Evaluations/ Study Schedule Part B/ Throughout all sections
Brief description of change:<br>Rapid antigen testing for SARS-CoV-2 was added on dosing days. SARS-CoV-2 testing (PCR as well as rapid antigen testing) can be performed throughout the study when clinically indicated and at the discretion of the investigator, or according to local requirements. The tests were added as sub sections.<br>On dosing days an update of I/E Criteria is sufficient.<br>Defined safety samples may be taken and randomization may be performed on pre-vaccination day.<br>A serum pregnancy test can replace a urine pregnancy test in case the screening was performed < 48 hours before vaccination.<br>Time windows for vaccinations was adapted as follows:<br>2nd vaccination: D28, time window of **±3** days<br>3rd vaccination: D56, v4+28, time window of **±3** days<br>4th vaccination: 224 (+28) days after 1st vaccination<br>Minimum of 21 days between two vaccinations.<br>Visit schedule was adapted accordingly.		
02	09DEC2021	8 Study objectives
Brief description of change:<br>Correction of wording of MERS-CoV-S related objectives and endpoints.<br>Assessment of sex differences in the immunity to vaccination was moved from exploratory endpoints to exploratory objectives.<br>Time for 4th vaccination changed.		
02	09DEC2021	9.3.2.1 Reproductive Potential
Brief description of change:<br>Update of contraceptive requirements: No contraception required for post-menopausal females, females with exclusively same-sex sexual partners or sexual abstinent subjects.		
02	09DEC2021	9.4.5 Blinding
Brief description of change:<br>Syringes will be blinded and the blinded study team will perform the vaccination.		
02	09DEC2021	9.4.7.4 Unscheduled Visit
Brief description of change:<br>Description of unscheduled visits to investigate and exclude the possibility of antibody-dependent enhancement (ADE) in case of SARS-CoV-2 infections or vaccinations against COVID-19.		
02	09DEC2021	9.6.2.2.13.1.1 Severity Categorization
Brief description of change:<br>Update of grading tables according to current versions of CTCAE version 5.0 and FDA guidance SEP-2007.		
02	09DEC2021	9.6.2.2.13.2.1 Reporting Procedures
Brief description of change:<br>All AESIs must be reported in the same way as SAEs.		

Summary of changes since last version of protocol (protocol version 3.0 to version 4.0)		
**Amendment Number**	**Date of Amendment**	**Section Affected by Change**
03	05OCT2022	Synopsis
Brief description of change: Extended follow-up phase Part B added.		
03	05OCT2022	Study Schedule
Brief description of change: Footnote p added for Part B: Use of SARS-CoV-2 rapid antigen test from within 4 hours before the screening visit. (according to clarification to protocol no. 06) Footnote q added: women who have had a hysterectomy do not need to have a pregnancy test (according to clarification to protocol no. 4)		
03	05OCT2022	6 Investigators and administrative structure
Brief description of change: Contact information updated.		
03	05OCT2022	8 Study Objectives
Brief description of change: Time interval added for the analyses of objectives/ endpoints.		
03	05OCT2022	9.4.1 Treatments Administered
Brief description of change: Information added on performance of visits in case a subject missed one vaccination (either the 2nd vaccination on D28 or the 3rd vaccination on D56). (according to clarification to protocol no. 05)		
03	05OCT2022	10.2 Additional Reports
Brief description of change: Information on the time point of preparation of the short report on the risks to human health or the environment resulting from the IMP added.		
03	05OCT2022	11 Extended follow-up phase Part B for exploratory objectives
Brief description of change: Section added.		

Summary of changes since last version of protocol (protocol version 4.0 to version 5.0)		
**Amendment Number**	**Date of Amendment**	**Section Affected by Change**
04	24NOV2022	Synopsis
Brief description of change: Documentation of SAEs and AESIs added.		
04	24NOV2022	11.1 Study Schedule Tabelle 6 „Part B – Extended Follow-up“
Brief description of change: Documentation of SAEs and AESIs added.		
04	24NOV2022	11.4.3.1 „Extended Follow-up visits“
Brief description of change: Documentation of SAEs and AESIs added.		
04	24NOV2022	11.4.3.2 „Early Termination“
Brief description of change: Documentation of SAEs and AESIs added.		

04	24NOV2022	11.4.3.3 „Adverse and Serious Adverse Events Assessments“)
Brief description of change:<br>Documentation of SAEs and AESIs added.		

2 SYNOPSIS

**Name of Sponsor/Company:** University Medical Center Hamburg-Eppendorf	**Individual Study Table Referring to Part of the Dossier**	**(For National Authority Use only)**
**Name of Finished Product:** MVA-MERS-S_DF-1	**Volume:**	
**Name of Active Ingredient:** MVA-MERS-S	**Page:**	
**Title of Study:**	A two-center, randomized, double-blind, placebo-controlled, phase Ib study to assess the safety, tolerability and immunogenicity of two ascending doses of the candidate vaccine MVA-MERS-S_DF-1 in healthy study subjects	
**Coordinating Investigator**	Prof. Dr. med. Marylyn Addo	
**Study centers:**	• CTC North GmbH at the University Medical Center Hamburg-Eppendorf Martinistr. 64, 20251 Hamburg, Germany<br>• Erasmus MC (EMC) – Department of Viroscience, Dr. Molewaterplein 50. 3015 GE Rotterdam, The Netherlands	
**Protocol-No.**	CEPI-MVA-MERS-S-Phase1b	
**EudraCT-No.**	2019-000715-83	
**Study Period**	Study is divided into two parts (Part A & Part B)<br>Part A:<br>Open-label run-in phase: period will last for 7 months per subject (screening to follow-up).<br><br>Part B:<br>Randomized controlled double-blind phase: period will last for approx. 10 months per subject (screening to follow-up).<br><br>Study Timelines:<br>Total study duration (FSFV to LSLV) approx. 17 months<br>Screening start is defined as FSFV.	
**Phase of development:**	Phase Ib	
**Objectives:**<br>**Primary Objective**	• To investigate the safety and tolerability of two ascending dose levels and two different dosing intervals of the candidate vaccine MVA-MERS-S_DF-1 in healthy study subjects until the end-of-study visit on D252.<br>• To investigate safety and tolerability of three intramuscular dose administrations of the candidate MVA-MERS-S_DF-1 vaccine in healthy study subjects using the immunization schedule D0/D28/D224 or D0/D56/D224 until the end-of-study visit on D252	

**Secondary Objectives**	• To evaluate MERS-CoV-S-specific antibody responses in healthy male and female study subjects induced by two dosage levels and two different dosing intervals of MVA-MERS-S_DF-1 until the end-of-study visit on D252
**Exploratory Objectives**	• To evaluate MERS-CoV-S-specific cellular immune responses after administration of MVA-MERS-S_DF-1, including MERS-CoV-S-induced B- and T-cell memory responses<br>• To evaluate humoral and cellular immunity against the viral vector MVA<br>• To evaluate pre-existing humoral and cellular immunity to coronaviruses<br>• To evaluate innate immune cell subset phenotypes and function induced by MVA-MERS-S_DF-1<br>• To evaluate early innate immunity gene expression signatures induced by MVA-MERS-S_DF-1<br>• To investigate vaccine-induced non-neutralizing humoral immune responses and antibody functions<br>• To assess sex differences in the immunity to vaccination
**Study Design**	Two-center, randomized, double-blind, placebo-controlled, dose-finding phase Ib study with an open-label run-in phase
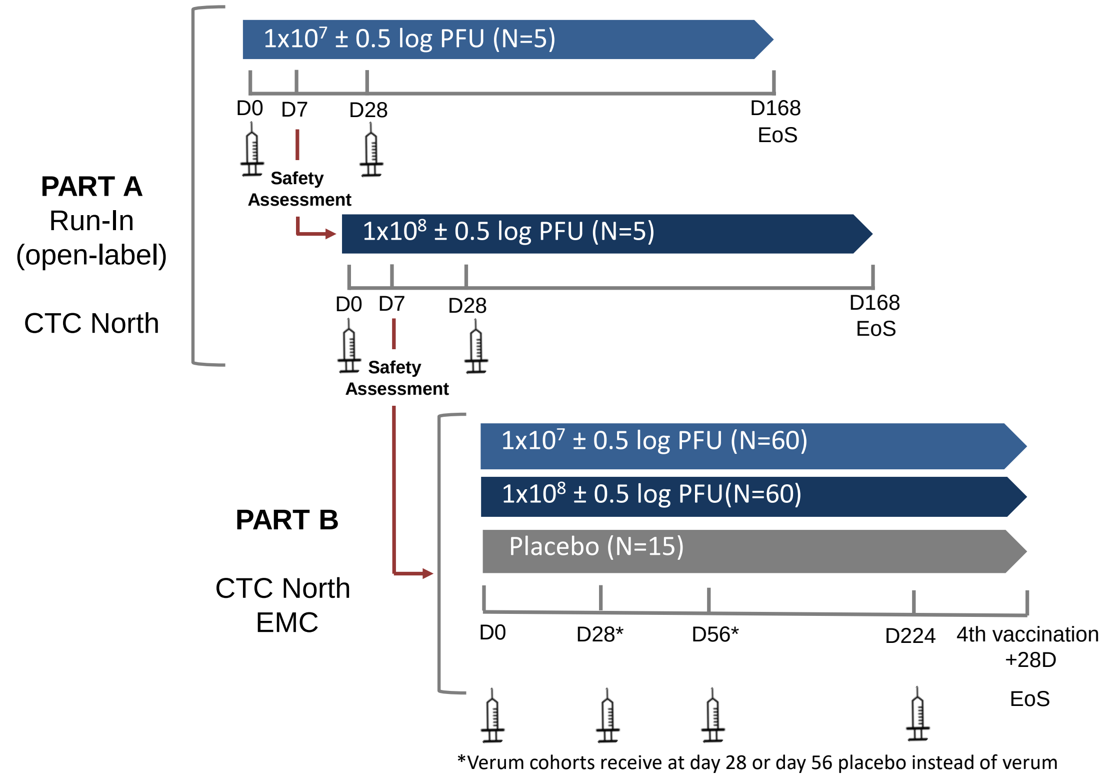 	
**Methodology:**	This will be a Phase Ib, two-center study in approximately 145 healthy adults aged 18-55 years.<br><u>Part A (N=10)</u><br>Study starts with an open-label run-in phase of two dose levels (low dose 1x10^{7} ± 0.5 log pfu, high dose 1x10^{8} ± 0.5 log pfu; 5 subjects per dose level). Part A will be conducted at the CTC North study site.

	For the decision about dose escalation in Part A, the Local Safety Board (LSB) will review safety data (AEs, vital signs, and laboratory safety data) obtained until day 7 after the 1st immunization of all subjects of the low dose cohort.<br><br>For the decision about starting Part B, the LSB will review safety data obtained until day 7 after the 1st immunization of all subjects of the low and high dose cohort from Part A. All subjects will be followed up for safety until D168.<br><br>Part B (N=135)<br><br>Two-center, randomized, double-blind, placebo-controlled, dose-finding study. Subjects will be allocated at CTC North and EMC. Subjects will be randomized to one of the dose cohorts (ratio 2:2:2:2:1):<br>• Low dose on day 0, 28, 224 and placebo on day 56<br>• Low dose on day 0, 56, 224 and placebo on day 28<br>• High dose on day 0, 28,224 and placebo on day 56<br>• High dose on day 0, 56, 224 and placebo on day 28<br>• Placebo on day 0, 28, 56, 224<br><br>Each subject will receive three single vaccine injections and 1 placebo injection, or 4 placebo injections.<br><br>All subjects will be followed up for safety until 28 days after the 4th vaccination.
**Number of subjects:**	N=145 (Part A: N=10, Part B: N=135)<br>Part A (open-label) (study site: CTC North)<br>5 subjects low dose cohort ($1x10^7 \pm 0.5$ log pfu MVA-MERS-S_DF-1)<br>5 subjects high dose cohort ($1x10^8 \pm 0.5$ log pfu MVA-MERS-S_DF-1)<br>Part B (double-blinded) (study sites: CTC North, EMC)<br>30 subjects in low dose on day 0, 28, 224 and placebo on day 56<br>30 subjects in low dose on day 0, 56, 224 and placebo on day 28<br>30 subjects in high dose on day 0, 28, 224 and placebo on day 56<br>30 subjects in high dose on day 0, 56, 224 and placebo on day 28<br>15 Placebo on day 0, 28, 56, and 224
**Key criteria for inclusion and exclusion:**	**Key inclusion criteria:**<br>1) Written informed consent form.<br>2) Healthy male and female subjects aged 18-55 years.<br>3) No clinically significant acute health problems as determined from medical history and physical examination at screening visit.<br>4) Body mass index 18.5 – 30.0 kg/m^2 and weight > 50 kg at screening.<br>5) Non-pregnant, non-lactating female with negative pregnancy test.<br>6) Females who agree to comply with the applicable contraceptive requirements of the protocol.<br><br>**Key exclusion criteria:**<br>1) Receipt of vaccination against MERS in medical history.<br>2) Receipt of any vaccine from 2 weeks prior to each trial vaccination (4 weeks for live vaccines) to 3 weeks after each trial vaccination.

	3) Known allergy to the components of the MVA-MERS-S_DF-1 vaccine product.<br>4) Evidence in the subject's medical history or in the medical examination that might influence either the safety of the subject or the absorption, distribution, metabolism or excretion of the investigational product.<br>5) Any confirmed or suspected immunosuppressive or immunodeficient condition, cytotoxic therapy in the previous 5 years, and/or diabetes.<br>6) Any chronic or active neurologic disorder, including seizures and epilepsy, excluding a single febrile seizure as a child.
**Test product, dose and mode of administration:**	**Test product:** MVA-MERS-S_DF-1<br>**Dose:**<br><br><u>Low dose cohort (LD):</u><br>65 (5 in Part A, 60 in Part B) subjects receiving MVA-MERS-S_DF-1 dose $1x10^7$ ± 0.5 log pfu /dose<br><u>High dose cohort (HD):</u><br>65 (5 in Part A, 60 in Part B) subjects receiving MVA-MERS-S_DF-1 dose $1x10^8$ ± 0.5 log pfu /dose<br><u>Placebo cohort:</u><br>15 subjects receiving placebo<br><br>**Mode of administration:** intramuscular (i.m.)
**Duration of Treatment:**	<u>Part A</u>: Subjects will receive two vaccine injections on D0 and D28 and will be followed up until D168 after 1st vaccination.<br><u>Part B</u>: Subjects will receive four injections (D0/D28/D56/224) and will be followed up until Day 28 after 4th vaccination.
**Criteria for evaluation:**	**Primary Endpoints:**<br>• Overall safety and tolerability of two ascending dose levels and two different dosing intervals of MVA-MERS-S_DF-1 administered at three time points<br>• Frequency and severity of local injection site reactogenicity signs and symptoms<br>• Occurrence and frequency of adverse events<br>• Change from baseline safety laboratory parameters<br><br>Frequency and severity of adverse events associated with MVA-MERS-S_DF-1 vaccine will be collected and measured as followed:<br>• Frequency of solicited local reactogenicity signs and symptoms for 7 days after vaccination<br>• Frequency of solicited systemic reactogenicity signs and symptoms for 7 days after vaccination<br>• Frequency of unsolicited adverse events for 28 days after vaccination (incl. clinically relevant laboratory parameters)<br>• Frequency of serious adverse events (SAE) throughout the study period<br><br>**Secondary Endpoints:**<br>Immunogenicity:

	• Humoral immunity: Magnitude of MERS-CoV-S-specific antibody responses (ELISA and neutralization assays) monitored in approved laboratories<br><br>**Exploratory Endpoints**<br>• Magnitude of cellular immune responses after administration of MVA-MERS-S_DF-1<br>• Magnitude of MVA-MERS-S-specific $CD4^+$ and $CD8^+$ T-cell responses by ELISpot and intracellular cytokine staining using flow cytometry<br>• Magnitude of non-neutralizing antibody responses against MVA-MERS-S<br>• Frequency and magnitude of pre-existing humoral and cellular immunity to non-MERS-CoV, including frequency of cross-reactive serologies<br>• Magnitude of early innate immune cell activation induced by MVA-MERS-S_DF-1<br>• Frequency of innate immune cells induced by MVA-MERS-S_DF-1<br>• Magnitude of early innate immunity gene expression signatures induced by the MVA-MERS-S_DF-1 vaccine<br>• Magnitude of humoral and cellular responses to the MVA vector
**Safety:**	Safety data (AEs, SAEs and vital signs) will be collected at all study visits until the end-of-study visit on D252.<br>Laboratory safety tests (biochemistry, hematology and dipstick urinalysis) will be performed at screening, pre-dosing D0, D28 or D56, and D224 and end of study visit on D252.
**Statistical methods:**	All subjects, who receive at least one injection, will be included in the safety analysis. For safety and tolerability assessments, descriptive statistics and relative and absolute frequencies will be displayed for adverse events, laboratory tests, vital signs, and physical examination. Graphs will be provided as appropriate for safety and tolerability assessments.<br>Immune-monitoring will be performed to define response to vaccination. All subjects who received at least two injections will be included in the per protocol population. Immune response will be defined by humoral immunity parameters. For immune response descriptive statistics will be created. Graphs will be provided as appropriate.<br>The sample size determination is not based on a formal statistical power calculation but the precision of the frequency estimates of adverse events. Based on the current sample size, statistical significance between groups can be detected if the difference in safety is 30% or more.<br>When all subjects completed 3 months in the study, one interim analysis is planned to determine optimal dose and injection scheme for future studies.
**Extended follow-up phase part B for exploratory objectives**	
**Study Period**	Subjects who consent in the extended follow-up phase will be followed up until 24 months (672 days) after the 4th vaccination.<br>End of trial: Last subject last visit defined as last extended follow-up visit.

**Exploratory Objectives**	• To evaluate MERS-CoV-S-specific antibody responses in healthy male and female study subjects until the end of the extended follow-up phase.<br>• To evaluate MERS-CoV-S-specific cellular immune responses, including MERS-CoV-S-induced B- and T-cell memory responses<br>• To evaluate long-term humoral and cellular immunity against the viral vector MVA<br>• To investigate long-term vaccine-induced non-neutralizing humoral immune responses and antibody functions<br>• To assess sex differences in the immunity to vaccination
**Safety**	Documentation of SAEs and AESIs.
**Study Design**	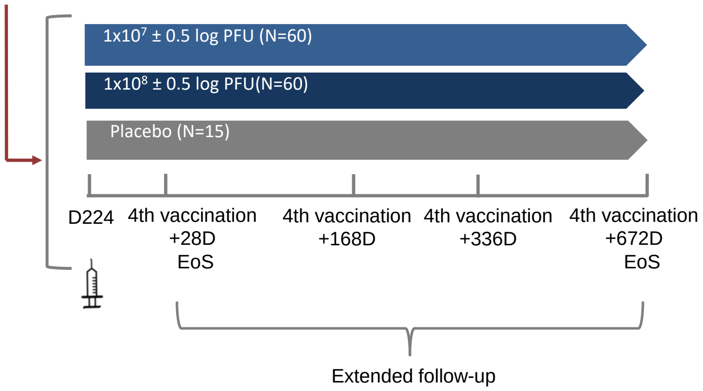
**Methodology:**	Subjects who consent in the extended follow-up phase will be followed up until 24 months (672 days) after the 4th vaccination.
**Inclusion and exclusion criteria**	**Inclusion criteria:**<br>1) Written informed consent for extended follow-up phase.<br>2) Enrollment in interventional part B of the clinical trial.<br>3) At least 2 vaccinations in the interventional part B and being no drop-out.<br><br>**Exclusion criteria:**<br>None
**Duration of Treatment**	No treatment is administered, only follow-up visits for immunological data from subjects who consent in the extended follow-up phase. Those subjects participate until 24 months (672 days) after the 4th vaccination.
**Statistical methods:**	Descriptive statistical analysis will be performed to investigate the durability of vaccine-induced immune responses.

STUDY SCHEDULES

Table 1 Study Schedules

PART A

Phase of study	SCR	Ambulatory Visits						End of study
**Study day**	**-28 – -1**	**0**	**7 (±1)**	**14 (±3)**	**28**	**35 (±3)**	**56 (±7)**	**168 (±14)**
**Study Months**		**0**			**1**		**2**	**6**
Informed Consent	√							
I/E Criteria	√	√[e]			√[e]			
PCR test SARS-CoV-2	√[i]	---------------------------- continuously[i] ------------------------						
Rapid Antigen Test SARS-CoV-2	------------------------------- continuously[m] -------------------------------							
Rapid Antigen Test SARS-CoV-2		√[e]			√[e]			
Medical History & Demographics	√	√[e,g]						
Physical Examination	√	√[a,e]			√[a,e]			
Height and Weight	√							
Vital Signs	√	√[b]			√[b]			√
12-lead ECG	√							√
Vaccination		√[d]			√[d]			
Injection site & systemic events/ reactions assessment		√[b]	√	√	√[b]	√		
Temperature	√	√[b]	√	√	√[b]	√	√	√
Alcohol Test	√	√[e]			√[e]			
Training & dispensing diary (electronic or paper)		√			√			
Review & collection diary (electronic or paper)			√	√	√	√	√	
Adverse Events & Concomitant medication		√[c]	√	√	√[c]	√	√	√

Study day	-28 – -1	0	7 (±1)	14 (±3)	28	35 (±3)	56 (±7)	168 (±14)
**Study Months**		**0**			**1**		**2**	**6**
Safety Lab blood (Clinical Chemistry, Hematology)	√	√[e,f]	√	√	√[e]	√	√	√
Urine Drug Screen	√	√[e,f]			√[e]			
Urine Pregnancy Test [q]	√							√
Serum Pregnancy Test [q]		√[e]			√[e]			
Safety Urinalysis	√	√[e,f]			√[e]			
HIV/HBV/HCV Serology	√							
Humoral responses		√[e]		√	√[e]	√	√	√
PBMC freezing and plasma aliquots		√[e]		√	√[e]	√	√	√

PART B

Phase of study	SCR				Ambulatory Visits									End of study
**Study Visit (v)**	**v0**	**v1**	**v2**	**v3**	**v4**	**v5**	**v6**	**v7**	**v8**	**v9**	**v10**	**v11**	**v12**	**v13**	**v14**
**Study day**	**-28 – -1**	**0**	**1**	**14 (±3)**	**28[l] (±3)**	**29[o] v4+1**	**42 v4+14 (±3)**	**56[l] v4+28 (±3)**	**57[o] v7+1**	**70 v7+14 (±3)**	**84 v7+28 (±7)**	**168 (±7)**	**224 (+28)**	**225[o] v12+1**	**v12+28 (+7)**
**Vaccination Visit**		**1**			**2**			**3**					**4**		
**Study Months**		**0**			**1**			**2**			**3**	**6**	**8-**		**9**
Informed Consent	√														
I/E Criteria	√	√[e,k]			√[e,k]			√[e,k]					√[e,k]		
PCR test SARS-CoV-2	-- continuously[i,n] --														
Rapid Antigen Test SARS-CoV-2	√[m,p]	--- continuously[m] ---													
Rapid Antigen Test SARS-CoV-2		√[e]			√[e]			√[e]					√[e]		
Randomization[n]		√[e,n]													
Medical History & Demographics	√	√[e,g]													
Physical Examination	√	√[a,e]			√[a,e]			√[a,e]					√[a,e]		
Height and Weight	√														
Vital Signs	√	√[b]			√[b]			√[b]					√[b]		√
12-lead ECG	√														√
Vaccination		√[d]			√[d,l]			√[d,l]					√[d]		
Injection site & systemic events/ reactions assessment		√[b]	√	√	√[b]	√	√	√[b]	√	√	√		√[b]	√	
Temperature	√	√[b]	√	√	√[b]	√	√	√[b]	√	√	√		√[b]	√	√
Alcohol Test	√	√[e]			√[e]			√[e]					√[e]		
Training & dispensing diary (electronic or paper)		√			√			√					√		
Review & collection diary (electronic or paper)			√	√	√	√	√	√	√	√	√			√	√
Adverse Events & Concomitant medication		√[c]	√	√	√[c]	√	√	√[c]	√	√	√	√	√[c]	√	√

Study Visit (v)	v0	v1	v2	v3	v4	v5	v6	v7	v8	v9	v10	v11	v12	v13	v14
Study day	-28 – -1	0	1	14 (±3)	28[l] (±31)	29[o] v4+ 1	42 v4+ 14 (±3)	56[l] v4+28 (±31)	57[o] v7+ 1	70 v7+ 14 (±3)	84 v7+ 28 (±7)	168 (±7)	224 (+28)	225[o] v12+1	v12+28 (+7)
Study Months		0			1			2			3	6	8		9
Safety Lab blood (Clinical Chemistry, Hematology)	√	√[e,f,n]	√	√	√[e,n]	√	√	√[e,n]	√	√			√[e,n]	√	√
Urine Drug Screen	√	√[e,f]			√[e]			√[e]					√[e]		
Urine Pregnancy Test [q]	√														√
Serum Pregnancy Test [q]		√[e,f,n]			√[e,n]			√[e,n]					√[e,n]		
Safety Urinalysis	√	√[e,f,n]			√[e,n]			√[e,n]					√[e,n]		
HIV/HBV/HCV Serology	√														
Humoral responses		√[e]		√	√[e]		√	√ [e]		√	√	√	√[e]		√
RNA blood sample[h]		√[e]	√	√	√[e]	√	√	√[e]	√	√			√[e]	√	
PBMC freezing and plasma aliquots[h]		√[e]	√	√	√[e]	√	√	√ [e]	√	√	√	√	√[e]	√	√

Please refer to section 9.4.7.4 for information on unscheduled visits.

[a] Symptom-targeted physical examination

[b] Please refer to Table 2 for frequency of vital signs examinations and temperature

[c] Please refer to Table 2 for frequency of Adverse Events and Concomitant Medication questioning

[d] Subjects will be monitored and evaluated for AEs for at least 1 hours after vaccination and only be discharged if no clinically significant abnormalities, to be judged by the PI, are measured and no other unexpected side effect are observed

[e] before immunization

[f] Samples do not need to be repeated if screening was performed < 48 hours before vaccination.

[g] Update of medical history only

[h] Samples for exploratory objectives can be collected optionally. The decision is made by the site-specific PI.

[i] PCR may be performed at screening and throughout the study at discretion of the Investigator and according to local requirement

[k] Update only

[l] Minimum of 21days between two vaccinations

[m] SARS-CoV-2 testing can be performed throughout the study when clinically indicated and at the discretion of the investigator, or according to local requirements

[n] May be performed one day before on a pre-vaccination day.

[o] If the vaccination was not administered on the previous vaccination visit but all other events were performed according to protocol this visit can be skipped

[p] For Part B: If SARS-CoV-2 rapid antigen test was performed within 4 hours before the screening visit and if the test result is suitable to be used as source data for the clinical trial, e.g. a test done by the study site as part of the hygiene concept, the test does not need to be repeated at screening and the available test results can be used

[q] Not applicable for women who have had a hysterectomy

Table 2 Detailed Time and Events

Study Day	Time after Dose admin-istration (hours)	Dosing	Vital Signs	Temperature	Adverse Events/ Con Med	Injection site/ systemic reac-tion/ Events as-sessment
0 Pre-dose	Pre-dose		√	√		
0	0	√				√ (within 5 minutes after vaccination)
0	1 (within 1h+30 minutes)		√	√	√	√
28 Pre-dose	Pre-dose		√	√	√	
28	0	√				√ (within 5 minutes after vaccination)
28	1 (within 1h+30 minutes)		√	√	√	√
56 Pre-dose	Pre-dose		√	√	√	
56	0	√				√ (within 5 minutes after vaccination)
56	1 (within 1h+30 minutes)		√	√	√	√
224 Pre-dose	Pre-dose		√	√	√	
224	0	√				√ (within 5 minutes after vaccination)
224	1 (within 1h+30 minutes)		√	√	√	√

Part A: only Day 0 and Day 28 applicable

3 TABLE OF CONTENTS

1 TITLE PAGE 1
2 SYNOPSIS 7
3 TABLE OF CONTENTS 17
3.1 List of Tables 19
3.2 List of Figures 19
4 LIST OF ABBREVIATIONS 20
5 ETHICS 23
5.1 Ethics Committee or Institutional Review Board 23
5.2 Ethical Conduct of the Study 23
5.3 Subject Information and Consent 23
5.4 Confidentiality 24
5.5 Insurance 24
5.6 Publication Policy 24
5.7 Qualification of the Investigator 24
6 INVESTIGATORS AND STUDY ADMINISTRATIVE STRUCTURE 25
7 INTRODUCTION PHARMACOLOGICAL CLASSIFICATION 27
7.1 Rationale 27
7.2 Clinical use 28
7.3 Preclinical results 28
7.4 Phlebotomy 28
7.5 Administration of IMP (Study Vaccine) 29
7.6 Risk-benefit considerations 29
8 STUDY OBJECTIVES 29
8.1 Primary Objective 29
8.1.1 Primary Endpoints 30
8.2 Secondary Objectives 30
8.2.1 Secondary Endpoints 30
8.3 Exploratory Objectives 30
8.3.1 Exploratory Endpoints 30
9 INVESTIGATIONAL PLAN 31
9.1 Overall Study Design and Plan-Description 31
9.2 Discussion of Study Design, including the Choice of Control Groups 32
9.3 Selection of Study Population 32
9.3.1 Inclusion Criteria 32
9.3.2 Exclusion Criteria 33
9.3.3 Removal of Subjects from Therapy or Assessment 35
9.3.4 Replacement of subjects 37
9.4 Treatments 37
9.4.1 Treatments Administered 37
9.4.2 Identity of Investigational Medicinal Product(s) 38
9.4.3 Method of Assigning Subjects to Treatment Groups 39

9.4.4 Selection of Doses in the Study ... 40
9.4.5 Blinding ... 40
9.4.6 Prior and Concomitant Therapy ... 40
9.4.7 Treatment Compliance ... 40
9.5 Protocol Deviations (PD) ... 44
9.6 Efficacy and Safety Variables ... 44
9.6.1 Efficacy and Safety Measurement Assessed and Flow Chart ... 44
9.6.2 Appropriateness of Measurements ... 44
9.6.3 Immunology Measurements ... 57
9.6.4 Safety Variables ... 59
9.7 Data Quality Assurance ... 60
9.7.1 Quality Assurance System ... 60
9.7.2 Monitoring ... 60
9.7.3 Documentation and Data Collection ... 60
9.7.4 Data Management ... 61
9.7.5 Archival of documents ... 61
9.8 Statistical Methods Planned in the Protocol and Determination of Sample Size ... 61
9.8.1 Statistical and Analytical Plan ... 61
9.8.2 Determination of Sample Size ... 62
9.9 Changes in the conduct of the study or planned analysis ... 63
9.10 Safety Boards ... 63
9.10.1 LSB ... 63
9.10.2 DSMB ... 63
10 REPORTS ... 63
10.1 Clinical Study Report ... 64
10.2 Additional Reports ... 64
11 EXTENDED FOLLOW-UP PHASE PART B FOR EXPLORATORY OBJECTIVES ... 65
11.1 Study Schedule ... 65
11.2 Subject Information and Consent ... 67
11.3 Study Objectives ... 67
11.3.1 Exploratory Objectives ... 67
11.3.2 Exploratory Endpoints ... 67
11.4 Investigational Plan ... 67
11.4.1 Inclusion Criteria ... 67
11.4.2 Exclusion Criteria ... 67
11.4.3 Treatments ... 68
11.4.4 Immunology Measurements ... 69
11.5 Statistical and Analytical Plan ... 69
11.6 Clinical Study Report ... 70
12 REFERENCES ... 71

3.1 List of Tables

Table 1 Study Schedules 13
Table 2 Detailed Time and Events 16
Table 3 Toxicity grading scale for local adverse events 51
Table 4 Toxicity grading scale for physical observations 51
Table 5 Toxicity grading scale for systemic AEs 52
Table 6: Part B Extended follow-up 65

3.2 List of Figures

Figure 1 Study Design. 32
Figure 2 Staggered approach for the dose administration of Part A. 38
Figure 3 Study Design extended follow-up phase part B 67

4 LIST OF ABBREVIATIONS

ADE	Antibody-Dependent Enhancement
ADEM	Acute Disseminated Encephalomyelitis
ADR	Adverse Drug Reaction
AE	Adverse Event
AEFI	Adverse Event Following Immunization
AESI	Adverse Event of Special Interest
ARDS	Acute Respiratory Distress Syndrome
BNITM	Bernhard Nocht Institute for Tropical Medicine
BP	Blood Pressure
bpm	beats per minute
CAU	Christian-Albrechts-Universität zu Kiel
CCMO	Central Committee on Research Involving Human Subjects
CD	Cluster of Differentiation
CEF	Chicken Embryo Fibroblasts
CEPI	Coalition for Epidemic Preparedness Innovations
CFR	Code of Federal Regulations
cm	centimeter
CNS	Central Nervous System
COGEM	Committee for Genetic Modification
CoV	Coronavirus
COVID-19	Coronavirus SARS-CoV-2
CRF	Case Report Form
CRO	Contract Research Organization
CS	Clinically Significant
CSR	Clinical Study Report
CV	Coefficient of Variation
DMP	Data Management Plan
DPP4	Dipeptidyl Peptidase IV
DRM	Data Review Meeting
DSMB	Data Safety Monitoring Board
EC	Ethics Committee
ECG	Electrocardiogram
eCRF	Electronic Case Report Form
EDTA	Ethylene Diamine Tetra-acetic Acid
ELISA	Enzyme-linked Immunosorbent Assay
EliSpot	Enzyme-linked Immunosorbent Spot
EMA	European Medicines Agency
EMC	Erasmus Medical Center
EoS	End of Study
EU	European Union
FDA	Food and Drug Administration
FOCP	Female of Child-bearing Potential
FSFV	First Subject First Visit

GBS	Guillain Barré Syndrome
GCP	Good Clinical Practice
GCP-V	Regulations on the implementation of Good Clinical Practice in the conduct of clinical trials on medicinal products for human use (GCP Verordnung)
GeoCV	Geometric Coefficient of Variation
GeoM	Geometric Mean
GLP	Good Laboratory Practice
h	Hour
HBsAg	HBV surface antigen
HBV	Hepatitis B Virus
HCV	Hepatitis C Virus
HD	High Dose
hDPP4	Human Dipeptidyl Peptidase IV
HIV	Human immunodeficiency virus
HSP	Henoch Schonlein purpura
i.m.	Intramuscular
IB	Investigator’s Brochure
ICH	International Conference on Harmonization of Technical Requirements for Registration of Pharmaceuticals for Human Use
IenW	Dutch Ministry of Infrastructure and Water Management
IFN-γ	Interferon-γ
IKMB	Institute of Clinical Molecular Biology
IMP	Investigational Medicinal Product
kg	Kilogram
KSA	Kingdom of Saudi Arabia
LD	Low Dose
LMU	Ludwig-Maximilians-Universität München
LSB	Local Safety Board
LSLV	Last Subject Last Visit
MDRD	Modification of Diet in Renal Disease
Mean	Arithmetic mean
Med	Median
MedDRA	Medical Dictionary for Regulatory Activities
MERS	Middle East Respiratory Syndrome
METC	Medisch Ethische Toetsings Commissie
mg	Milligram
min	Minute
mL	Milliliter
mm	millimeter
mmHg	Millimeters of mercury
MSV	Master Seed Virus
MVA	Modified Vaccinia Virus Ankara
MVA-MERS-S	Modified Vaccinia virus Ankara recombinant virus expressing Middle East Respiratory Syndrome Coronavirus Spike protein

N	Number
NCS	Not Clinically Significant
NOAEL	No Observed Adverse Event Level
PBMC	Peripheral Blood Mononuclear Cell
PCR	Polymerase Chain Reaction
PD	Protocol deviation
PFU	Plaque-forming Unit
PI	Principal Investigator
QAU	Quality Assurance Unit
S	Spike
SAE	Serious Adverse Event
SAP	Statistical Analysis Plan
SARI	Severe Acute Respiratory Infection
SARS	Severe Acute Respiratory Syndrome
SAS	Statistical Analysis Software
SD	Standard Deviation
SDV	Source Data Verification
SLE	Systemic Lupus Erythematosus
SOP	Standard Operating Procedure
SPEAC	Safety Platform for Emergency vACcines
SUSAR	Suspected Unexpected Serious Adverse Reaction
TMF	Trial Master File
UKE	University Medical Center Hamburg-Eppendorf
VNT	Virus Neutralisation Test
WHO	World Health Organization

5 ETHICS

5.1 Ethics Committee or Institutional Review Board

This study will be planned and performed in accordance with

- The Declaration of Helsinki in its version of Fortaleza, 2013;
- The EU Clinical Trial Directive 2001/20/EC;
- The EU Clinical Trial Directive 2001/83/EC
- ICH Guideline for Good Clinical Practice E6(R2), of 9 November 2016;
- Guideline on Strategies to Identify and Mitigate Risks for First-In-Human Clinical Trials with investigational medicinal products (CHMP/SWP/28367/07)
- Guidance on the Management of Clinical Trials during the COVID-19 (Coronavirus) Pandemic in its most current version

and other applicable laws.

5.2 Ethical Conduct of the Study

The Sponsor authorizes CR2O to make all necessary applications for the Netherlands and CTC North for all necessary applications for Germany.

CR2O and CTC North will submit all required documents according to local law to the responsible ethics committees (EC), as well as the competent authorities and other agencies if needed (see section 5.1 *Ethics Committee or Institutional Review Board*, page 23) for approval.

The approval of these authorities must be obtained prior to the start of the study in the respective study sites in Germany and the Netherlands.

Copies of the original submission and approval documents will be sent to the Sponsor and will be included in the Clinical Study Report (CSR). A list of the members of the Ethics Committee will also be provided with the CSR.

The Netherlands:

- An application for release of a Genetically Modified Organism, MVA-MERS-S_DF-1, into the environment will be submitted to the Dutch Committee for Genetic Modification (COGEM). The submission will be made by a Biosafety Officer on behalf of the Erasmus Medical Centre Rotterdam. The Dutch Ministry of Infrastructure and Water Management (IenW) will make the final decision on whether to issue a permit to Erasmus MC for performing this clinical trial, based on the recommendations made by the COGEM.
- Ethical approval in the Netherlands will be sought from the Dutch competent authority, the Central Committee on Research Involving Human Subjects (CCMO).
- If no objections are made by the institutions mentioned above, final permission to initiate the trial will be obtained from the local Institutional Review Board of Erasmus MC after feasibility analysis.

5.3 Subject Information and Consent

Before any study specific procedures can take place, an investigator will explain to the subjects the nature, significance and implications of the study. The investigator will explain all methods, rules of conduct and any restrictions which may apply. Possible effects and side effects will be discussed. Subjects will be informed that they are free to withdraw from the study at any time, without giving any reason for doing so. They must be able to understand the full implications of their decision.

All participants will date and sign an informed consent form as evidence of consent. The investigator will also date and sign the informed consent form. The subject information sheet and the informed consent form of each participant will be filed in a study binder. A copy of the signed consent form and the information sheet will be handed to the subjects after signature and before enrollment.

The initial informed consent form, any subsequent revised written informed consent form and any written information provided to the subject must receive the EC's approval in advance of use. The subject should be informed in a timely manner if new information becomes available that may be relevant to the subject's willingness to continue participation in the study.

5.4 Confidentiality

The Principal Investigator (PI) must assure that subjects' anonymity will be strictly maintained and that their identity is protected from unauthorized parties. Only an identification code (i.e., consists of identification number, sex and year of birth) should be recorded on any form or biological sample submitted to the laboratory, Sponsor or Ethics Committee. The PI must keep a Subject Identification log showing codes and names for all subjects screened and enrolled in the trial.

5.5 Insurance

The Sponsor is responsible for the appropriate insurance coverage for the subjects.

5.6 Publication Policy

The Sponsor has to publish the result of this study considering applicable legal requirements and the CEPI publication policy. It is at the discretion of the sponsor to lead the publication activities. The trial will be registered at clinicaltrials.gov.

5.7 Qualification of the Investigator

The PIs and their deputies or sub investigators fulfill the requirements of applicable law. Curriculum vitae of the PIs and deputies/sub investigators will be filed in the trial master file (TMF).

For conducting the study, the PI may delegate tasks to investigators (or other qualified staff). This is to be documented properly. The PI is responsible for the adequate training and supervision of all delegates. No study related procedure must be performed by personnel which is not properly trained and delegated.

In the present document the mere term "Investigator" refers to the PIs or the deputy/deputies.

6 INVESTIGATORS AND STUDY ADMINISTRATIVE STRUCTURE

**Sponsor:**	University Medical Center Hamburg-Eppendorf<br>Martinistr. 52, 20246 Hamburg, Germany
**Coordinating Investigator:**	Prof. Dr. Marylyn M. Addo<br>Division of Infectious Diseases, I. Med. Department<br>University Medical Center Hamburg-Eppendorf<br>Martinistr. 52, 20246 Hamburg, Germany<br>Phone: [redacted]<br>Fax: [redacted]<br>Email: [redacted]
**Study Site Hamburg:**	Prof. Dr. Marylyn M. Addo (Principal Investigator)<br>CTC North GmbH<br>at the University Medical Center Hamburg-Eppendorf<br>Martinistr. 64, 20251 Hamburg, Germany<br>Phone: [redacted]<br>Fax: [redacted]
**Study Site Rotterdam:**	Prof. Dr. Eric van Gorp (Principal Investigator)<br>Erasmus Medical Centre<br>Dr. Molewaterplein 40<br>3015 GD Rotterdam<br>Phone: [redacted]<br>Email: [redacted]
**Medical Monitor:**	Dr. Stefan Schmiedel<br>Division of Infectious Diseases, I. Med. Department<br>University Medical Center Hamburg-Eppendorf<br>Phone: [redacted]<br>Email: [redacted]
**Global CRO**	CR2O<br>at the Toren “Copenhagen” 6th floor<br>Bisonspoor 3002 – C701, 3605 LT Maarssen, The Netherlands<br>Hadil Es-Sbai<br>Phone: [redacted]<br>Email: [redacted]
Responsibilities:	Overall Project Management, Regulatory & Monitoring Netherlands
**German CRO**	CTC North GmbH<br>at the University Medical Center Hamburg-Eppendorf<br>Martinistr. 64, 20251 Hamburg, Germany<br>Laura Kaltenberg<br>Phone: [redacted]<br>Fax: [redacted]<br>Email: [redacted]
Responsibilities:	Local Project Management, Regulatory Germany, Medical Writing, Data Management, Safety Management
**Monitoring Germany**	University Medical Center Hamburg-Eppendorf<br>Martinistr. 52, 20246 Hamburg, Germany

Statistics: Staburo GmbH
Aschauer Str. 30a, 81549 München, Germany
Josef Höfler
Phone: [redacted]
Fax: [redacted]
Email: [redacted]

Safety Laboratories

Site 01 (Hamburg)	Site 02 (Rotterdam)
Center for Diagnostics<br>University Medical Center Hamburg-Eppendorf<br>Martinistr. 52, 20246 Hamburg, Germany	Erasmus Medical Centre<br>Dr. Molewaterplein 40<br>3015 GD Rotterdam, The Netherlands

In consultation with the Sponsor selected safety examinations can be carried out at the study sites

Immunogenicity Laboratories:

Humoral Immunity	Clinical Virology
Prof. Dr. Stephan Becker<br>Institute for Virology<br>Philipps University Marburg<br>Hans Meerweinstr. 2<br>35043 Marburg, Germany	Erasmus Medical Centre<br>Dr. Molewaterplein 40<br>3015 GD Rotterdam<br>The Netherlands
**Humoral Immunity/Vector Immunity**	**Humoral Immunity**
Prof. Dr. Gerd Sutter<br>Institute for Infectious Diseases and Zoonoses<br>Ludwig-Maximilians-Universität München<br>Veterinärstr. 13<br>80539 Munich, Germany	Dr. Bart Haagmans<br>Erasmus Medical Centre<br>Ee building 17th floor<br>Dr Molewaterplein 50<br>3000 CA Rotterdam, The Netherlands
**T cell Immunity**	**Sequencing Projects**
Prof. Dr. Marylyn M. Addo<br>Department of Clinical Immunology of Infectious Diseases<br>Bernhard-Nocht-Institute<br>Bernhard Nocht Str. 74<br>20357 Hamburg, Germany	Prof. Dr. Andre Franke<br>Institute of Clinical Molecular Biology (IKMB)<br>Kiel University, Christian-Albrechts-Universität zu Kiel (CAU)<br>Rosalind-Franklin-Str. 12<br>24105 Kiel, Germany

7 INTRODUCTION PHARMACOLOGICAL CLASSIFICATION

7.1 Rationale

MERS and the need for a MERS Vaccine

In the aftermath of the dramatic and unprecedented Ebola outbreak in West-Africa 2013-2016, for which the world and scientific community were ill-prepared, the WHO initiated a Research and Development "Blueprint Initiative for action to prevent epidemics" (http://www.who.int/csr/research-and-development/blueprint/en/). In this context the WHO convened an ad-hoc expert group to synthesize lessons learned from past global health experiences and agreed on a list of emerging pathogens likely to cause severe outbreaks and for which no or limited medical countermeasures exist (http://www.who.int/medicines/ebola-treatment/WHO-list-of-top-emerging-diseases/en/). MERS-CoV, the causative agent of the Middle Eastern Respiratory Syndrome (MERS), is one of the priority organisms for which such preparedness efforts were be initiated through international collaboration.

MERS is a potentially fatal disease under tight epidemiologic control by the WHO and currently without registered prevention or treatment options. The first case of MERS-CoV infection was identified in a patient with acute pneumonia and renal failure in the Kingdom of Saudi Arabia (KSA) in June 2012 [1]. As of August 2021, 2578 laboratory-confirmed cases of MERS-CoV and 888 deaths have been reported, resulting in a case-fatality rate of 34.4% (WHO Situation Report). MERS-CoV shows an expanding geographical distribution. While infections have been mainly observed in the Middle East, 27 countries have reported MERS cases [2]. To date three cases have been imported to Germany, all with fatal outcome.

Dromedary camels serve as reservoir hosts and the majority of primary human cases can be traced back to dromedary contact. Individuals with close and frequent contact to dromedaries are therefore at high-risk for MERS-CoV infection. Additionally, human-to-human transmission can occur, specifically amplified in health-care settings, identifying health-care workers as an additional high-risk population. The virus affects men and women of all ages. Disease severities induced by MERS-CoV infections vary from silent infections or mild respiratory symptoms to severe pneumonia with acute respiratory distress syndrome, sepsis and multi-organ failure. Some cases develop renal failure concurrently with respiratory failure [3].

The causative agent MERS-CoV expresses the surface glycoprotein named spike (S) protein, which is responsible for binding to the human host cell receptor Dipeptidyl Peptidase IV (DPP4) and mediates viral attachment to host cells, entry and membrane fusion [4]. DPP4 has a broad tissue distribution in humans (including bronchial and renal epithelia). Bat, civet, pig and rabbit DPP4 orthologs may support infection of corresponding cell lines, while canine, feline, rodent and chicken cells were found non-susceptible [5,6]. Based on recent research that revealed a key role of MERS-CoV-S for vaccine development, several MERS vaccine candidates express the full MERS-CoV-S protein.

Vaccine candidates that are based on viral vectors have been shown to induce robust immune responses. In the last decade, the vaccine platform based on the Modified Vaccinia Virus Ankara (MVA) revealed safe and immunogenic profiles. MVA is a replication-deficient viral vector that can be engineered to encode one or more foreign antigens [7]. Several vaccine candidates based on MVA demonstrated induction of cell-based and humoral immune responses. The vector can be used at biosafety level 1 and has intrinsic adjuvant properties.

The MVA-based vaccine against MERS expresses the MERS-CoV-S protein, which has been shown to induce neutralizing antibodies against MERS-CoV in a range of animal models. Facing the human pathogenicity of MERS-CoV and the ongoing MERS cases with increasing geographic distribution, clinical studies to evaluate tolerability and immunogenicity of the vaccine candidate MVA-MERS-S in humans are urgently warranted.

From 2017 to 2018 a first-in-human study was performed in Hamburg (University Medical Center Hamburg Eppendorf and CTC North GmbH) to investigate MVA-MERS-S in 23 healthy adults using a prime-boost regimen. For this trial, MVA-MERS-S was produced on primary chicken embryo fibroblasts (CEF) cells and used for an open, single center phase I trial to assess the safety, tolerability and immunogenicity of two ascending doses (EudraCT No. 2014-003195-23, registration number NCT03615911). Immunizations with MVA-MERS-S revealed a benign safety profile with only transient mild-to-moderate reactogenicity. The safety profile was similar after prime and boost immunization. Participants experienced no severe or serious adverse events. Local reactions, headache and fatigue were the most common AEs. All AEs resolved swiftly and without sequelae. Following booster immunization, 87% of all

vaccinees and 100% of high-dose vaccine recipients showed seroconversion using an S1-ELISA. Antibody titers measured by ELISA correlated well with MERS-S-specific neutralizing antibodies. MERS-CoV-S-specific T-cell responses were detected in 91% of all vaccine recipients. A dose-effect relationship was observed for reactogenicity but not for immunogenicity.

The Coalition for Epidemic Preparedness Innovations (CEPI) has now included the MVA-MERS-S vaccine in its portfolio to fund further clinical development of this vaccine candidate. To be prepared for outbreak responses, CEPI is funding a novel scalable technology to enable stockpile production of the vaccine, necessary for outbreak response. Starting material for this manufacturing process aiming to produce a high stockpile of MVA-MERS-S_DF-1 vaccine will be the master seed virus (MSV) bank established on CEF cells that was tested in the above-mentioned Phase Ia study.

7.2 Clinical use

The MVA platform is a promising vaccine platform, as multiple clinical trials against a variety of infectious diseases revealed a safe and immunogenic profile for MVA-based vaccines. Clinical trials showed the induction of multimodal humoral and cellular immune responses to the target antigen. More than 130 clinical trials testing MVA were published in PubMed. No single study reported any SAE.

In 2018/2019, we performed a Phase I trial testing safety, tolerability and immunogenicity with the MVA-MERS-S vaccine (EudraCT No. 2014-003195-23, NCT03615911; PI: M.M. Addo). MVA-MERS-S was administered using a 28-day interval. The data demonstrated a beneficial safety and immunogenicity profile. Vaccinees experienced no SAE.

The second generation vaccine candidate MVA-MERS-S_DF-1 was built on the same MVA platform that was tested in the Phase Ia. In contrast to the first generation vaccine, MVA-MERS-S_DF-1 was produced on the primary cell line DF-1. The use of DF-1 cells in combination with the CellStack technology enables a large-scale production of MVA-MERS-S_DF1, which is critical for outbreak scenarios.

7.3 Preclinical results

The experimental vaccine MVA-MERS-S has been studied in several animal models as outlined in the investigator's brochure (IB). More specifically, primary pharmacology data were obtained in the following models and studies:

- Mouse immunogenicity (Song F et al., 2013)
- Protection from MERS-CoV challenge in transiently transduced hDPP4 mice (Volz A et al., 2015)
- Rat immunogenicity (as part of GLP compliant pivotal nonclinical toxicology study)
- Dromedary camels (Haagmans BL et al., 2016)

To date there is no appropriate non-human-primate model established for MERS that models human disease in a satisfactory manner.

The second generation vaccine candidate was additionally tested in a bridging study. In concordance with the study by Volz et al., DPP4 mice were vaccinated to compare both vaccine candidates. The results demonstrate comparable vaccine-induced adaptive immune responses. Furthermore, a toxicology study in rabbits was applied, confirming the planned clinical high dose as No Observed Adverse Event Level (NOAEL) dose in the species. Details are listed in the IB.

7.4 Phlebotomy

For part A, the total amount of blood collected from a volunteer will not exceed 400 mL over 7 months. For part B, the total amount of blood collected from a volunteer will be a maximum of 700 mL over 9 months until the end-of-study visit on D252. No more than 500 mL are collected within 12 weeks. These blood volumes should not endanger the otherwise healthy volunteers and should correspond to the volumes of voluntary blood donations. There may be slight bruising, local sensitivities, presyncopic symptoms associated with venipuncture or phlebitis, which may later lead to thrombosis. These events are not documented as AE when they occur.

7.5 Administration of IMP (Study Vaccine)

Serious allergic reactions including anaphylaxis may occur and for this reason subjects will be inoculated in a clinical area where Advanced Life Support trained physicians, equipment and drugs are immediately available for the management of any serious adverse reactions. Participants will be observed for 1 hour after vaccination. Subjects will be monitored and evaluated for AEs closely for at least 30 minutes after administration of vaccine with appropriate medical treatment readily available in case of a rare anaphylactic reaction.

7.6 Risk-benefit considerations

The participation in a Phase Ib study may not be of a therapeutic benefit to healthy subjects. Known risks related to the pharmacological properties of the investigational compound and/or study modalities are possible. To our best knowledge and judgement, the IMP seems to be safe and no severe side effects and no unacceptable adverse drug reactions (ADR) are expected with this study, as the used MVA vaccine vector has been extensively used in clinical trials for other infectious diseases threats encompassing in >6500 individuals, including children, cancer patients and immune-compromised hosts. The vaccine vector has had an excellent safety profile and unexpected adverse reactions conferred by the antigenic insert are not anticipated. The Phase Ia study testing MVA-MERS-S showed no single SAE.

All relevant preclinical studies required for the start of clinical development have been conducted. Preclinical data suggest a favorable safety and tolerability profile. Based on available information and the design of the study, the Sponsor and the Coordinating Investigator consider the trial to be ethically acceptable. The duration of confinement, the medical surveillance and the chosen study design for the sequential dosing of Part A and Part B are considered adequate to ensure safety of the subjects. Special consideration was given to adequate spacing of dosing and frequent safety assessments as mandated and recommended by the regulatory authorities.

The vaccine recipients may benefit from protection against future MERS outbreaks. However, this trial is the first administrations of MVA-MERS-S_DF-1 vaccine to humans. Therefore, to date the risks-benefit ratio remains unknown. Participants will be strongly advised to not consider themselves protected against MERS after vaccination at this stage of vaccine development.

In summary, preclinical and clinical data indicate a favorable safety and tolerability profile. So far, clinical trials on MVA have reported transient and spontaneously resolved adverse events and no serious ones. The duration of confinement, the medical surveillance and the chosen time intervals for the sequential dosing of the first, second and third subjects of each dosing cohort are considered adequate to ensure maximal safety of the subjects and have been established with guidance of the competent authority. Thus, taking into account the safety measures to minimize risks for study participants, the exposure of healthy subjects with MVA-MERS-S vaccine is justified since the potential risks and disadvantages for study participants are outweighed by the potential benefits for medical research, medical practice and eventually for individuals at risk of MERS infection.

Risk Assessment with regards to COVID-19:

The sponsor and investigators carefully reviewed the feasibility, risk and benefits of starting a new clinical trial during the ongoing pandemic. Given the fact that MVA-MERS-S represents an emergency vaccine for another WHO blueprint organism and pandemic threat coronavirus there is an immediate necessity to continue with the MVA-MERS-S vaccine development program. The safety of the trial participants is of primary importance, and all measures will be taken to minimize the risk to trial participants and prioritize trial participant safety and data validity.

8 STUDY OBJECTIVES

8.1 Primary Objective

This study is designed to investigate the safety and tolerability of two ascending dose levels and two different dosing intervals of the candidate vaccine MVA-MERS-S_DF-1 in healthy study subjects and to investigate safety and tolerability of three intramuscular dose administrations of the candidate MVA-MERS-S_DF-1 vaccine in healthy study subjects using the immunization schedules D0/D28/D224 or D0/D56/D224 until the end-of-study visit on D252.

8.1.1 Primary Endpoints

- Overall safety and tolerability of two ascending dose levels and two different dosing intervals of MVA-MERS-S_DF-1 administered at three time points
- Frequency and severity of local injection site reactogenicity signs and symptoms
- Occurrence and frequency of adverse events
- Change from baseline safety laboratory parameters

Frequency and severity of adverse events associated with MVA-MERS-S_DF-1 vaccine will be collected and measured as followed:

- Frequency of solicited local reactogenicity signs and symptoms for 7 days after vaccination
- Frequency of solicited systemic reactogenicity signs and symptoms for 7 days after vaccination
- Frequency of unsolicited adverse events for 28 days after vaccination (incl. clinically relevant laboratory parameters);
- Frequency of serious adverse events (SAE) throughout the study period until the end-of-study visit on D252

8.2 Secondary Objectives

This study is designed to evaluate MERS-CoV-S-specific antibody responses in healthy male and female study subjects induced by two dosage levels and two different dosing intervals of MVA-MERS-S_DF-1 until the end-of-study visit on D252.

8.2.1 Secondary Endpoints

Immunogenicity:

- Humoral immunity: Magnitude of MERS-CoV-S-specific antibody responses (ELISA and neutralization assays) monitored in approved laboratories until the end-of-study visit on D252

8.3 Exploratory Objectives

Samples for exploratory objectives can be collected optionally (please refer to Table 1 *Study Schedules*). The decision is made by the site-specific principal investigator. Therefore, only a subset of patients will be included in the analysis of exploratory endpoints.

- To evaluate MERS-CoV-S-specific cellular immune responses after administration of MVA-MERS-S_DF-1, including MERS-CoV-S-induced B- and T-cell memory responses until the end-of-study visit on D252
- To evaluate humoral and cellular immunity against the viral vector MVA until the end-of-study visit on D252
- To evaluate pre-existing humoral and cellular immunity to coronaviruses until the end-of-study visit on D252
- To evaluate innate immune cell subset phenotypes and function induced by MVA-MERS-S_DF-1 until the end-of-study visit on D252
- To evaluate early innate immunity gene expression signatures induced by MVA-MERS-S_DF-1 until the end-of-study visit on D252
- To investigate vaccine-induced non-neutralizing humoral immune responses and antibody functions until the end-of-study visit on D252
- To assess sex differences in the immunity to vaccination until the end-of-study visit on D252

8.3.1 Exploratory Endpoints

- Magnitude of cellular immune responses after administration of MVA-MERS-S_DF-1 until the end-of-study visit on D252

- Magnitude of MVA MERS-S-specific CD4+ and CD8+ T-cell responses by ELISpot and intra-cellular cytokine staining using flow cytometry until the end-of-study visit on D252
- Magnitude of non-neutralizing antibody responses against MVA-MERS-S until the end-of-study visit on D252
- Frequency and magnitude of pre-existing humoral and cellular immunity to non-MERS-CoV, including frequency of cross-reactive serologies until the end-of-study visit on D252
- Magnitude of early innate immune cell activation induced by MVA-MERS-S_DF-1 until the end-of-study visit on D252
- Frequency of innate immune cells induced by MVA-MERS-S_DF-1 until the end-of-study visit on D252
- Magnitude of early innate immunity gene expression signatures induced by the MVA-MERS-S_DF-1 vaccine until the end-of-study visit on D252
- Magnitude of humoral and cellular responses to the MVA vector until the end-of-study visit on D252

The results from exploratory objectives will not be part of the final study report.

9 INVESTIGATIONAL PLAN

9.1 Overall Study Design and Plan-Description

This will be a Phase Ib, two-center study in approximately 145 healthy adults aged 18-55 years.

The study is separated in two parts:

Part A:

The study starts with a single center open-label run-in phase of two dose levels (cohort 1 “low dose”: $1x10^7$ ± 0.5 log pfu, cohort 2 “high dose”: $1x10^8$ ± 0.5 log pfu) in 10 healthy subjects. 5 subjects will be allocated to each dose cohort and will receive immunization on day 0 and day 28. Part A will only be conducted at the CTC North study site.

Dosing of cohort 2 (HD) will not start before favorable safety assessment of the safety data obtained until day 7 after the 1st immunization of all subjects of the low dose cohort by the local safety board (LSB).

Part B will not start before favorable safety assessment of the safety data obtained until day 7 after the 1st immunization of all subjects of the high dose cohort by the LSB.

Part B:

Two-center, randomized, double-blind, placebo-controlled, dose-finding study. This part is a double-blinded trial in approximately 135 healthy subjects.

Subjects will be randomized to one of the dose cohorts (ratio 2:2:2:2:1):

- Low dose on day 0, 28, 224 and placebo on day 56
- Low dose on day 0, 56, 224 and placebo on day 28
- High dose on day 0, 28, 224 and placebo on day 56
- High dose on day 0, 56, 224 and placebo on day 28
- Placebo on day 0, 28, 56, 224

Each subject will receive three single vaccine injections and 1 placebo injection, or 4 placebo injections.

Part B will be conducted in two centers, CTC North and Erasmus MC.

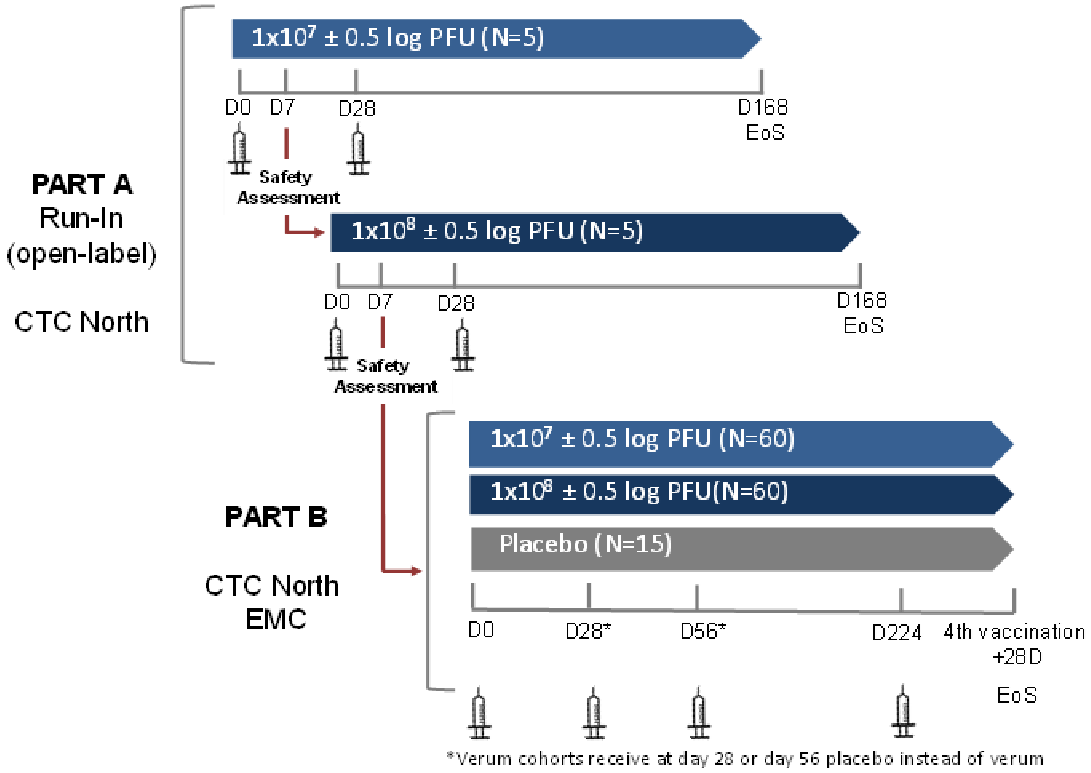

Figure 1 Study Design.

9.2 Discussion of Study Design, including the Choice of Control Groups

The study will be a two-center, randomized, double-blind, placebo controlled study of the MVA-MERS-S_DF-1 candidate delivered by i.m. injection. To evaluate the MERS-S-specific antibody responses and safety profile induced by the two dosage levels of MVA-MERS-S_DF-1 the data will be compared to a placebo control group.

9.3 Selection of Study Population

Approximately 145 healthy male and female subjects, aged between 18 and 55 years will be enrolled in this clinical trial. The subjects will be recruited from the study centers' subject pools and public advertisement. Allocation to a certain treatment number (or subject number) will be done in successive order following screening and based on the subjects' availability.

9.3.1 Inclusion Criteria

The subject must not be enrolled before all inclusion criteria (including test results) are confirmed. Subjects meeting all of the criteria listed below will be included in the study:

1. Ability to understand the subject information and to personally name, sign and date the informed consent to participate in the study.
2. Provided written informed consent.
3. Healthy male and female subjects aged 18 – 55 years inclusive at the time of consent. The date of signing informed consent is defined as the beginning of the screening period. This inclusion criterion will only be assessed at the first screening visit.
4. No clinically significant health problems as determined during medical history and physical examination and clinical laboratory results at screening visit. Following laboratory parameters should be within normal limits: WBC, ANC, platelets. AST, ALT and ALP should be ≤ULN, CrCL >60ml/min and total bilirubin should not exceed 1,5 x ULN. Non-clinically significant, minor deviations of laboratory measurements can be tolerated as long as they will not increase the risk

of the individual having an adverse outcome from participating in this study as judged by the investigator. Deviations of laboratory measurements from the reference ranges of more than grade 1 according to Common Toxicity Criteria lead to an exclusion of the subject.

5. Body weight in defined relation to height. Body mass index 18.5 – 30.0 kg/m^2 and weight >50 kg at screening.
6. Non-pregnant, non-lactating female with a negative pregnancy test at screening and on dosing days (prior to vaccination).
7. Females of child-bearing potential who agree to comply with the applicable contraceptive requirements of the protocol (section 9.3.2.1 *Reproductive Potential*) from at least 14 days prior first vaccination to day 168 (Part A)/day v12+28 (Part B) or females who are permanently sterilized (at least 6 weeks post-sterilization).
8. Be willing to refrain from blood donation during the course of the study.
9. The subject is co-operative and available for the entire study.

9.3.2 Exclusion Criteria

Subjects are excluded from the study if any of the following criteria are met at screening or at dosing days:

1. Prior receipt of a MERS vaccine in medical history.
2. Receipt of any vaccine in the 2 weeks prior to each trial vaccination (4 weeks for live vaccines) or planned receipt of any vaccine in the 3 weeks following each trial vaccination.
3. Known allergy to the components of the MVA-MERS-S_DF-1 vaccine product as chicken proteins or history of life-threatening reactions to vaccine containing the same substances.
4. Known history of anaphylaxis to vaccination or any allergy likely to be exacerbated by any component of the trial vaccine.
5. Participation in a clinical trial or use of an investigational product within 30 days or five times the half-life of the investigational product -whichever is longer- prior to receiving the first dose within this study.
6. Evidence in the subject's medical history or in the medical examination that might influence either the safety of the subject or the absorption, distribution, metabolism or excretion of the investigational product under investigation.
7. Clinically relevant findings in ECG, in particular prolonged QTc (B) of > 450 msec in males and > 460 msec in females.
8. Any positive result for HIV1/2, HCV antibody or HBs antigen testing.
9. Any confirmed or suspected immunosuppressive or immunodeficient condition, cytotoxic therapy in the previous 5 years, and/or diabetes.
10. Subjects with inflammatory, infectious and neuroinflammatory underlying disease which could cause an expected impairment of the blood brain barrier such as meningitis, multiple sclerosis, epilepsy, or Alzheimer's disease.
11. Any chronic or active neurologic disorder, including seizures and epilepsy, excluding a single febrile seizure as a child.
12. Known history of Guillain-Barré Syndrome.
13. Active malignancy or history of metastatic or hematologic malignancy.
14. Suspected or known alcohol and/or illicit drug abuse within the past 5 years. Recreational use of THC is allowed at discretion of the Investigator.
15. Moderate or severe illness and/or fever >38 °C within 1 week prior to vaccination.
16. Administration of immunoglobulins and/or any blood products within the 120 days preceding study entry or planned administration during the study period. Oral or parenteral immunosuppressant and immunomodulating agents (including interferons) should be discontinued at least 4 weeks prior to first injection.

17. History of blood donation within 60 days of enrollment or plans to donate within the treatment phase (until the 2nd (Part A) or 3rd (Part B) vaccination).
18. Receipt of chronic (defined as more than 14 days) immune suppressants or other immune-modifying drugs within 6 months of study inclusion (screening).
    - For corticosteroids, this will mean prednisone, or equivalent, greater than or equal to 0.5 mg/kg/day.
    - Intranasal and inhaled steroids are allowed. Topical steroids are permitted provided they are not required to be applied to injection site.
19. Subjects with skin lesions close to the injection site or active oral lesions will be excluded.
20. Thrombocytopenia, contraindicating intramuscular vaccination based on investigator's judgment.
21. Subjects with a significant infection or known inflammation.
22. History of relevant cardiovascular disorders or evidence of hyper- (sitting blood pressure systolic >140 or diastolic >90 mmHg) or hypotension (sitting blood pressure systolic <90 or diastolic <40 mmHg) at screening.
23. Subjects who are known or suspected not to comply with the study directives.
24. Any other significant finding that in the opinion of the investigator would increase the risk of the individual having an adverse outcome from participating in this study.
25. Investigator or employee of the study site or Sponsor with direct involvement in the proposed study, or identified as an immediate family member (i.e., parent, natural or adopted child) of the investigator or employee with direct involvement in the proposed study.

9.3.2.1 Reproductive Potential

The study population includes female of child-bearing potential (FOCP). FOCP have to agree to comply with the applicable contraceptive requirements of the protocol as named below for the duration of the study. This does not apply for females who are permanently sterilized (at least 6 weeks post-sterilization) or are post-menopausal (post-menopausal is defined as no menses for 12 months without alternative medical cause and ≥ 47 years) or females with exclusively same-sex sexual partners.

Effective contraception is defined as a contraceptive method with failure rate of less than 1% per year when used consistently and correctly and when applicable, in accordance with the product label for example:

- Oral contraceptives, either combined or progesterone alone
- injectable progesterone
- implants of etonogestrel or levonorgestrel
- estrogen vaginal ring
- percutaneous contraceptive patches
- intrauterine device or intrauterine system
- male partner sterilization at least 6 months prior to the female subject's entry into the study, and a monogamous relationship
- male condom combined with a vaginal spermicide (foam, gel, film, cream or suppository)
- male condom combined with a female diaphragm, either with or without a vaginal spermicide (foam, gel, film, cream, or suppository)

Condoms are to be used with the mentioned acceptable contraceptives.

No contraception is required for sexually abstinent subjects. Sexual abstinence (from at least 14 days prior randomization until end of study): acceptable only if it is the participant's preferred and usual form of birth control/lifestyle choice.

9.3.2.2 Restrictions

Subjects may be asked to fast for at least 6 hours prior to dose administration in the morning of dosing days. There is no fasting required after application of IMP.

9.3.3 Removal of Subjects from Therapy or Assessment

The study in its entirety may be discontinued prematurely by the Sponsor at any time (see below) for safety reasons, and/or individual subjects may terminate their participation prematurely, or have their participation be terminated by the investigator.

9.3.3.1 Withdrawal of Subject from the Study

The following circumstances may lead to discontinuation of the study by an individual subject who will then be recorded as a drop-out include but are not limited to the following:

- Withdrawal for personal reasons
- Adverse events necessitating withdrawal from the study
- Sudden incidence of diseases
- Circumstances in which the health of the subject would be endangered upon continued participation in the study
- Subject non-compliance with study requirements
- An AE, which requires discontinuation of the study involvement or results in inability to continue to comply with study procedures
- Significant protocol violation
- Lost-to follow-up
- Pregnancy
- Other (must be specified)

The reason for withdrawal will be recorded in the electronic case report form (eCRF). If withdrawal is due to an AE, appropriate follow-up visits or medical care will be arranged, with the agreement of the subject, until the AE has resolved, stabilized or a non-trial related causality has been assigned. The LSB or DSMB may also recommend withdrawal of subjects.

At least 3 documented attempts must be made within 2 weeks to contact any subject lost to follow-up at any time point prior to the last scheduled office visit. One of the documented attempts must include a written communication (e.g. post mail, courier) with acknowledgement of receipt requested (e.g. certified mail, registered mail) sent to the subject's last known address, requesting that they return any required material (e.g. diary) and return to the study site for final safety evaluations.

A replacing subject will be assigned as described in section 9.3.4 *Replacement of subjects* (page 37).

9.3.3.2 Handling Subjects with Premature Study Discontinuation

In the absence of a medical contraindication or significant protocol violation, every effort will be made by the investigator to keep the subject in the study. If a subject has to be withdrawn, all efforts will be made to complete and report the trial observations as thoroughly as possible. If a subject needs to be withdrawn, all efforts shall be made to follow safety of the subject as per protocol.

Subjects who withdraw or are withdrawn from the study prior to the first injection or subjects who received the first vaccination but are withdrawn without medical reasons before the second vaccination will be replaced. An early termination visit will be performed for all drop-outs. This visit may be a phone visit.

When a subject withdraws from the study after the first vaccination and before the planned end of the study period, all investigations scheduled for the end-of-study visit should be performed if the subject agrees. End-of-study evaluation will be completed at the time of the subject's withdrawal, with an explanation of the reason for this entered onto the respective "end-of-study" section of the eCRF as follows:

- Adverse event (specify)
- Death (specify)

- Protocol violation (specify)
- Medical condition (specify)
- Consent withdrawal, not due to AE
- Lost-to follow-up
- Other (specify)

9.3.3.3 Criteria for Termination of the Study (Holding Rules)

Safety holding rules will apply throughout the entire study period. Should a holding rule be activated, the investigator will inform the Sponsor. Study evaluation will be discussed with the LSB/DSMB. The Sponsor has to inform the Competent Authorities and the Ethics Committee within the timelines defined by national law.

If the event(s) occur in the higher-dose group, the follow-up phase of the lower dose group will continue. The discontinuation of a holding rule should be communicated to all entities in the same manner and timeframe as described above.

The LSB/DSMB safety review will consider:

- The relationship of the AE or SAE to the IMP
- The relationship of the AE or SAE to the IMP dose, or other possible causes of the event
- If appropriate, additional screening or laboratory testing for other subjects to identify those who may develop similar symptoms will be discussed

All injected subjects will be followed for safety until resolution or stabilization (if determined to be chronic sequelae) of their AE. For further details on AE grading please refer to Table 3, Table 4, and Table 5.

<u>The holding rules are as follows:</u>

<u>Part A</u>

- Solicited (expected) local adverse events:
    - If more than 2 injections are followed by Grade 3 solicited swelling or pain or Grade 4 redness beginning within 3 days after injection and persisting at Grade 3 (swelling or pain)/4 (redness) for >48 hours
- Solicited (expected) systemic adverse events:
    - If more than 2 injections are followed by Grade 3 solicited systemic AE (or Grade ≥ 3 physical observations) beginning within 3 days after study injection and persisting at Grade ≥ 3 for >48 hours
- Unsolicited (unexpected) adverse events:
    - If more than 2 individuals develop a Grade ≥ 3 unsolicited AE (including laboratory AE and physical observations) that is considered probably or definitely related to injection and persists at Grade 3 for >48 hours
- Any treatment related SAE
- A suspected unexpected serious adverse drug reaction (SUSAR) occurs that is life-threatening or results in death

<u>Part B</u>

With an interval of 40 subjects enrolled an evaluation of holding rules will be performed.

- Solicited (expected) local adverse events:
    - If more than 25% of injections are followed by Grade 3 solicited swelling or pain or Grade 4 redness beginning within 3 days after injection and persisting at Grade 3 (swelling or pain)/4 (redness) for > 48 hours
- Solicited (expected) systemic adverse events:

  - If more than 25% of injections are followed by Grade 3 solicited systemic AE (or Grade ≥ 3 physical observations as defined above and in section 9.6.2.2.13.1.1 *Severity Categorization*, page 51) beginning within 3 days after study injection and persisting at Grade ≥ 3 for > 48 hours

- Unsolicited (unexpected) adverse events:
  - If more than 25% of subjects develop a Grade ≥ 3 unsolicited AE (including laboratory AE and physical observations) that is considered probably or definitely related to injection and persists at Grade ≥ 3 for > 48 hours
- Any treatment related SAE
- A suspected unexpected serious adverse drug reaction (SUSAR) occurs that is life-threatening or results in death

Or

The Sponsor may terminate the trial at any time or a regulatory authority may request the termination. In the case of study termination, investigators will be informed of the procedures to be followed to ensure adequate consideration is given to the protection of the subject's safety.

In case any holding or stopping rules apply Sponsor needs to be informed within 24 hours. The Sponsor has to inform the Competent Authorities and the Ethics Committee within 15 days.

9.3.4 Replacement of subjects

In case a subject will be replaced as outlined in section 9.3.3.2 *Handling Subjects with Premature Study Discontinuation* (page 35) the replacement subject will be assigned to the subject number as described in section 9.4.3 *Method of Assigning Subjects to Treatment Groups* (page 39). Replacement subject will be assigned to the same treatment sequence as the subject he replaces.

9.4 Treatments

9.4.1 Treatments Administered

All eligible subjects will be assigned to one of the dose cohorts (Part A: low dose, high dose; Part B: low dose: placebo on day 56 or day 28; high dose: placebo on day 56 or day 28; placebo) according to the randomization list. Each subject in Part A will receive two vaccine injections administered as i.m. injections in the deltoid region of the upper arm muscle. Subjects in Part B will receive three vaccine injections and one placebo injection or 4 placebo injections.

Subjects in Part B that missed one vaccination (either the 2nd vaccination on D28 or the 3rd vaccination on D56) because of a reason that does not lead to an exclusion from the study can follow the regular study schedule incl. the next vaccination(s). If the vaccination was not administered on the previous vaccination visit but all other events were performed according to protocol the visit one day after the vaccination can be skipped.

Part A will be performed in a staggered manner. For safety reasons dose administration in each dose level will be performed in at least three groups. The first subject at both levels will be vaccinated 24 hours prior to the second group (second and third subject) of this dose level. The second and the third subject will be vaccinated in an interval of at least 5 min and after a period of at least 24 hours after the first subject to assess for immediate hypersensitivity reaction (within 30 min after the vaccination) or cytokine release (within 24 hours following the vaccination). Dosing events are supervised by an investigator.

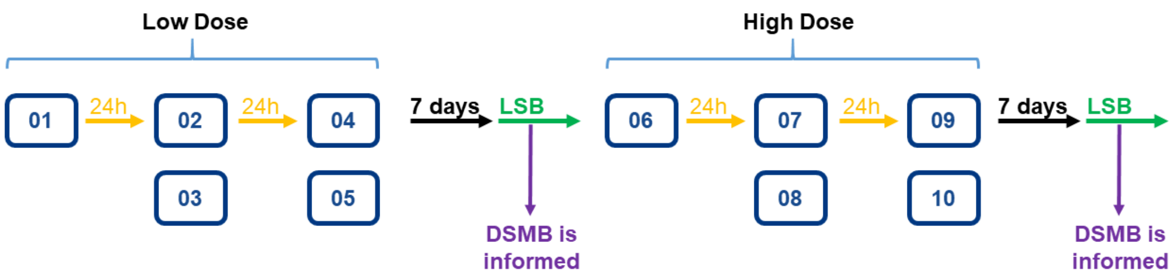

Figure 2 Staggered approach for the dose administration of Part A.

A Data Safety Monitoring Board (DSMB) will be established for this clinical trial.

For the decision about dose escalation in Part A, a Local Safety Board (LSB), will review safety data (AEs, vital signs, and laboratory safety data) obtained until day 7 after the 1st immunization of all subjects of the low dose cohort.

For the decision about entering Part B, the LSB will review safety data obtained until day 7 after the 1st immunization of all subjects of the high dose cohort.

The whole DSMB will be informed about the LSB decisions from Part A and will perform a continuous review of safety data obtained from Part B.

9.4.2 Identity of Investigational Medicinal Product(s)

Name: MVA-MERS-S_DF-1

Dosage form: Suspension for injection

Active substances: MVA-MERS-S

Route: i.m. injections

Manufacturer: IDT Biologika GmbH, Dessau

Name: Placebo

Dosage form: Suspension for injection

Active substances: NA

Components: 30 mM Tris, 6% Sucrose, 0.01% Polysorbat 80, pH 7.7

The Placebo composition was selected based on composition of the MVA-MERS-S_DF-1 vaccine candidate and consists of the same components as the formulation buffer.

Route: i.m. injections

Manufacturer: IDT Biologika GmbH, Dessau

Additional information can be found in the Investigator’s Brochure.

9.4.2.1 Labeling

The study vaccine and placebo will be provided labeled in accordance with applicable laws by IDT Biologika GmbH, Dessau to the study sites.

9.4.2.2 Packaging

The Sponsor will provide study vaccine and placebo for this study. Packaging and labeling of IMP is performed by IDT Biologika GmbH, Dessau. Labels will be written in accordance to local law.

9.4.2.3 Storage

The PI has overall responsibility for ensuring that study medication is stored under appropriate conditions in a secure, limited-access location. Study medication is distributed by a nominated member of the study team.

Study medication must be stored in accordance with labeled storage conditions (≤ -65 °C). Temperature monitoring is required at the storage location to ensure that the study medication is maintained within an established temperature range.

The PI is responsible for ensuring that the temperature is monitored throughout the total duration of the clinical trial and that records are maintained; the temperature should be monitored continuously by using an in-house system such that at least minimum and maximum temperatures over a specific time period can be recorded and retrieved as required.

The Sponsor has to be informed by the clinical trial site of any excursion from the established range as soon as the PI becomes aware of an excursion. The Sponsor has then to confirm the stability of the IMP before further use. Relevant temperature excursions will require site investigation as to cause and re-mediation. The Sponsor will determine the ultimate impact of excursions on the IMP and will provide supportive documentation as necessary.

9.4.2.4 Shipment

The IMP will be provided by the Sponsor in sufficient quantity.

Details of the shipment processes are defined in the Pharmacy Manual.

The batch number of the IMP used in the study will be given in the CSR.

9.4.2.5 Drug Accountability

The PI has the overall responsibility for administering the IMP. The IMP must be administered in the manner specified in the study protocol and the pharmacy manual.

The study site will acknowledge receipt of the study medication documenting shipment content and condition. Damaged supplies will be replaced. Accurate records of all study medication received, dispensed, used, returned or destroyed must be maintained. No study medication may be destroyed or returned from the investigational site without prior knowledge and written consent by the Sponsor. If such transfer is authorized by the Sponsor, all applicable local, state and national laws must be adhered for the transfer.

An Investigator or a delegated member of the study team will administer the IMP. All administrations will be documented in the site's drug accountability log or other study drug record. The PI is responsible for assuring the retrieval of all dispensed study supplies from subjects.

The Sponsor or its representatives must be permitted access to review the supplies storage and distribution procedures and records.

At the end of the study or as instructed by the Sponsor all unused stocks and empty used boxes are sent to a nominated contractor or will be destroyed at the study sites on behalf of the Sponsor. Study medication being returned or destroyed must be counted and verified by clinical investigational site personnel and the PI or deputy. All certificates of delivery/drug receipts should be signed by the site representative to confirm contents of shipment. Shipment return forms must be signed prior to shipment by the study site. The Sponsor must give authorization to return or destroy any study medication prior to shipment or destruction. Shipment of all returned study medication must comply with local, state, and national laws.

Based on the entries in the site drug accountability logs, it must be possible to reconcile study medication delivered with those used and returned. One hundred percent of the study medication must be accounted for and all discrepancies investigated and documented.

9.4.2.6 Subject Compliance

Compliance must be assessed by an investigator.

For study medication administration at the study site, compliance must be assessed by observation of dosing. Designated members of the study team will record details on the drug accountability log or other appropriate source documents.

9.4.3 Method of Assigning Subjects to Treatment Groups

Subjects willing and eligible to participate in this study will be randomized to a treatment cohort according to the randomization list. Detailed process of subject identification and randomization of subject numbers can be found in section 9.4.7.2 *Subject Identification* (page 41). The first 5 subjects in Part A will be

allocated to the low dose cohort, the following 5 subjects to the high dose cohort. Subjects in Part B will be randomized to one of the five groups:

- low dose: placebo on day 56 (n=30)
- low dose: placebo on day 28 (n=30)
- high dose: placebo on day 56 (n=30)
- high dose: placebo on day 28 (n=30)
- placebo cohort (n=15).

9.4.4 Selection of Doses in the Study

The doses selected in this study represent immunogenic dose levels as observed in prior clinical trials using MVA-vector-based vaccines, including a first-in-human pilot trial with the same MVA-MERS-S vaccine construct.

9.4.5 Blinding

As Part A is an open-label study, no blinding procedures are necessary for this part.

Part B is a randomized, double-blind study. Because the placebo and the vaccine are slightly different in aspect, volume and presentation the study will be conducted in a blinded manner for the study team, the Sponsor and designees including laboratory personnel, and the subjects. To ensure blinding for vaccines and blinded study team members, the vaccine preparation will be done by authorized medical or pharmacist personnel (unblinded study team or pharmacist) who will not participate in any of the study events or clinical evaluation of the study by wrapping a blinding tape (e.g. translucent yellow tape) around the syringe. During the study, the access to the IMP will be restricted to the designated unblinded site staff or pharmacist in charge of the vaccine accountability and preparation. The blinded syringes will be handed over by the unblinded study team to the blinded study team. Vaccination will be performed by an authorized blinded study team member. Unblinded and blinded site staff must be identified on the Site Delegation Log.

Emergency envelopes should only be opened if there is an AE that needs unblinding or the investigator decides for safety reason that unblinding is deeply necessary. Emergency envelopes should be maintained in a secure location during the study with 24/7 access. Please refer to the unblinding instructions filed in the Investigator Site File. Any code break will be documented and reported to the Sponsor (or a designee) in a timely manner. In a medical emergency, the Investigator may unblind the treatment for that subject without prior consultation with the Sponsor. In such an event, the investigator will need to contact the Sponsor or a designee as soon as possible after the unblinding to discuss the case.

9.4.6 Prior and Concomitant Therapy

Prior use of any drugs is forbidden as defined in section 9.3.2 *Exclusion Criteria* (page 33). Prior to each dosing, subjects will be asked about concomitant use of drugs. If a subject has used a drug, the investigator has to decide about the exclusion of the subject in consultation with the Sponsor.

Any use of concomitant medication will be documented specifying the substance, dose, time and reason for use.

For symptomatic management of flu-like symptoms, the use of anti-pyretic drugs (e.g. paracetamol/ibuprofen) in the recommended daily dose is permitted after consultation with the investigator.

Concurrent use of any prescription or non-prescription medication, or concurrent vaccination, during the course of the trial (i.e. after randomization or treatment allocation) must first be discussed at least between the investigator and the site's PI prior to administration, unless appropriate medical care necessitates that therapy or vaccination should begin before consultation. The subject will be allowed to continue in the trial if both the PI and the investigator agree. Paracetamol/ibuprofen may be used for minor ailments without consultation with the PI.

9.4.7 Treatment Compliance

9.4.7.1 Admission to the study

Admission to the study will be effective upon the subject's arrival at the study ward. A subject will only be admitted to the study if the Informed Consent Form has been signed and all inclusion and none of

the exclusion criteria are met. Should there be any doubts as to the state of health, a subject will not be admitted to the study.

Subjects who fail to make themselves available upon commencement of the study or who cannot participate for personal reasons will be considered as not admitted to the study. They will be replaced by back-up subjects who will be screened in surplus.

9.4.7.2 Subject Identification

Ascending screening numbers are assigned sequentially per site to all subjects as they consent to take part in the study.

Additionally, subjects that a randomized via the eCRF receive a randomization number which allocates a subject to a treatment cohort. A replacement subject will receive the same treatment as the withdrawn subject.

9.4.7.3 Screening, Confinement and Regular Visits

9.4.7.3.1 Screening (Day -28 to Day -1)

On screening visit the following events will be performed:

- Informed consent
- Inclusion/exclusion criteria
- PCR and rapid antigen test for SARS-CoV-2 may be performed at screening at discretion of the Investigator and according to local requirement
- Medical history/ demographics
- Physical examination
- Height and weight
- Vital signs
- 12-lead ECG
- Temperature
- Alcohol test
- Safety laboratory blood (clinical chemistry, hematology)
- Urine drug screen
- Urine pregnancy test
- Safety laboratory urine
- HIV/HBV/HCV serology

9.4.7.3.2 Regular Ambulatory Visits

Part A

Subjects will visit the study ward for regular ambulatory visits on study days 0 (1st dosing), 7 (±1), 14 (±3), 28 (2nd dosing), 35 (±3) and 56 (±7).

On the ambulatory visits the following events will be performed:

- Inclusion/exclusion criteria on day 0 and 28
- Update medical history on day 0, before immunization
- Physical examination on day 0 and 28 (symptom-targeted, before immunization)
- Vital signs on day 0 and 28
- Vaccination on day 0 and 28
- Injection site & systemic events/ reactions assessment on day 0, 7, 14, 28 and 35

- Temperature on day 0, 7, 14, 28, 35 and 56
- Alcohol test on day 0 and 28
- Training & dispensing diary on day 0, 28
- Review & collection diary on day 7, 14, 28, 35 and 56
- Adverse events & concomitant medication assessment on day 0, 7, 14, 28, 35 and 56
- Safety lab blood (clinical chemistry, hematology) on day 0*, 7, 14, 28, 35 and 56
- Urine drug screen on day 0* and 28
- Serum pregnancy test on day 0* and 28
- Safety lab urine on day 0* and 28
- Humoral responses on day 0, 14, 28, 35 and 56
- PBMC freezing and plasma aliquots on day 0, 14, 28, 35 and 56
- Rapid antigen test for SARS-CoV-2 on day 0 and 28 as required
- PCR and rapid antigen test for SARS-CoV-2 may be performed throughout the study at discretion of the Investigator and according to local requirement

After dosing the following events will be performed **at 1 h (to be completed within 30 minutes):**

- Vital signs (and at 0 h before dose administration)
- Temperature (and at 0 h before dose administration)
- Adverse events/ concomitant medication
- Injection site & systemic events/ reactions assessment (and at 0 h within 5 minutes after dose administration)

* Samples do not need to be repeated if screening was performed < 48 hours before vaccination.

Part B

Subjects will visit the study ward for ambulatory visits on study days 0 (1st dosing), 1, 14 (±3), 28 (±3) (2nd dosing), 29, 42 (±3), 56 (±3) (3rd dosing), 57, 70 (±3), 84 (±7), 168 (±7), 224 (+28), and 225 (4th dosing). Study vaccinations need to be at least 21 days apart. Please refer to Table 1 for detailed information on the timing of study visits depending on previous visit numbers.

On the ambulatory visits the following events will be performed:

- Update of inclusion/exclusion criteria on day 0, 28, 56 and 224
- Randomization on day 0 (or -1 day), before immunization
- Update medical history on day 0, before immunization
- Physical examination on day 0, 28, 56 and 224 (symptom-targeted, before immunization)
- Vital signs on day 0, 28, 56 and 224
- Vaccination on day 0, 28, 56 and 224
- Injection site & systemic events/ reactions assessment on day 0, 1, 14, 28, 29, 42, 56, 57, 70, 84 and 224, 225
- Temperature on day 0, 1, 14, 28, 29, 42, 56, 57, 70, 84 and 224, 225
- Alcohol test on day 0, 28, 56 and 224
- Training & dispensing diary on day 0, 28, 56, 224
- Review & collection diary on day 1, 14, 28, 29, 42, 56, 57, 70, 84, and 225
- Adverse events & concomitant medication assessment on day 0, 1, 14, 28, 29, 42, 56, 57, 70, 84, 168 and 224, 225

- Safety lab blood (clinical chemistry, hematology) on day 0* (or -1 day), 1, 14, 28 (or -1 day), 29, 42, 56 (or -1 day), 57, 70 and 224 (or -1 day), 225
- Urine Drug Screen on day 0*, 28, 56 and 224
- Serum pregnancy test on day 0* (or -1 day), 28 (or -1 day), 56 (or -1 day) and 224 (or -1 day)
- Safety lab urine on day 0* (or -1 day), 28 (or -1 day), 56 (or -1 day) and 224 (or -1 day)
- Humoral responses on day 0, 14, 28, 42, 56, 70, 84, 168 and 224
- RNA blood sample on day 0, 1, 14, 28, 29, 42, 56, 57 and 70, 225 (Samples for exploratory objectives can be collected optionally. The decision is made by the site-specific PI.)
- PBMC freezing and plasma aliquots on day 0, 1, 14, 28, 29, 42, 56, 57, 70, 84, 168 and 224, 225 (Samples for exploratory objectives can be collected optionally. The decision is made by the site-specific PI.)
- Rapid antigen test for SARS-CoV-2 on day 0, 28, 56 and 224
- PCR and rapid antigen test for SARS-CoV-2 may be performed throughout the study at discretion of the Investigator and according to local requirement

After dosing the following events will be performed **at 1 h (to be completed within 30 minutes):**

- Vital signs (and at 0 h before dose administration)
- Temperature (and at 0 h before dose administration)
- Adverse events/ concomitant medication
- Injection site & systemic events/ reactions assessment (and at 0 h within 5 minutes after dose administration)

* Samples do not need to be repeated if screening was performed < 48 hours before vaccination.

As a urine pregnancy test is part of the screening events and a serum pregnancy test is planned before the dosing, only a serum pregnancy test has to be done if the screening was performed < 48 hours before vaccination.

9.4.7.3.3 End of study/Early termination visit

An end-of-study follow-up visit will be performed on day 168 (±14) for part A and on v12 + 28 days (+7) for part B. The following events will be performed:

- Vital signs
- 12-lead ECG
- Temperature
- Review & collection diary
- Adverse events & concomitant medication assessment
- Safety lab blood (clinical chemistry, hematology)
- Urine pregnancy test
- Humoral responses
- PBMC freezing and plasma aliquots (Samples for exploratory objectives can be collected optionally. The decision is made by the site-specific PI.)
- PCR and Rapid antigen test for SARS-CoV-2 as required

9.4.7.4 Unscheduled Visit

Unscheduled visits may be performed at any time during the study. Depending on the reason for the unscheduled visit (e.g., AE, abnormal laboratory values, ECG), appropriate assessments will be performed based on the judgment of the investigator. Results of the assessment and any changes in concomitant treatment will be recorded in the eCRF. After an unscheduled visit, the regular scheduled study visits must continue according to the planned visit and assessment schedule.

An unscheduled visit can be performed in case a subject develops a SARS-CoV-2 infection or is vaccinated against COVID-19 to investigate and exclude the possibility of antibody-dependent enhancement (ADE).

9.4.7.5 Early Termination

All enrolled subjects who complete the study, discontinue early, or withdraw from the study will have the follow-up assessments and procedures completed for their safety as outlined in Table 1 *Study Schedules* (page 13). Subjects who discontinue early or withdraw from the study will be replaced as outlined in section 9.3.3.2 *Handling Subjects with Premature Study Discontinuation* (page 35).

In some cases it may be necessary for subjects to return to the study ward for additional care, confinement, and/or follow-up. Circumstances in which this may be necessary are:

- Follow-up on abnormal laboratory evaluations
- Follow-up on an ongoing AE at the final visit

All additional safety follow-up visits will be at the discretion of the, PI/deputy and the Sponsor..

9.5 Protocol Deviations (PD)

All PDs will be tracked and actions will be defined, as feasible. All PDs classified as major will be reviewed in Data Review Meetings for the interim analysis and the final analysis for assessment of their influence on the quality of the study analysis. Protocol deviations due to COVID-19 will be tracked and reported together with the other protocol deviations in the CSR.

9.6 Efficacy and Safety Variables

9.6.1 Efficacy and Safety Measurement Assessed and Flow Chart

Details regarding scheduled assessments and procedures to be conducted in this study are provided below. For detailed assessment of schedules refer to Table 1 *Study Schedules* (page 13).

All relevant laboratories involved in this trial are accredited for the assays performed. Continuous compliance with legal requirements throughout the trial will be assured.

9.6.2 Appropriateness of Measurements

9.6.2.1 Screening Procedures

Written, signed, and dated informed consent from the subject prior to the performance of any study related procedures must be obtained by an investigator. Subjects will first have ample time to read the subject information before an investigator will start the information and informed consent process. The subject information/informed consent process will be performed according to the study site's SOPs. The investigator will provide the subjects with information of the study and explain the nature of the study point by point. During this verbal subject information process subjects have already the opportunity to ask questions. After that, the subjects have the opportunity to individually ask questions in a one-to-one meeting with the investigator. If the investigator is convinced that the subject understands the nature and risks of the trial, and each subject had ample time for consideration and formulation of questions (which could also mean that the subjects first discuss the decision with friends or family members), and if all questions are answered the subject will asked to personally date and sign the informed consent form. A copy of the signed informed consent form must be given to the subjects for their records.

Screening procedures must be completed between 28 days and 1 days prior to receiving the first dose of study medication. See Table 1 *Study Schedules* (page 13) for a complete list of screening procedures to be performed. Re-checks may be performed for individual events as long as they are within the screening window.

Only an authorized and trained investigator may decide on the eligibility of the subject.

9.6.2.1.1 Screening Failure

A screening failure is defined as a subject who has given informed consent and failed to meet at least one inclusion or exclusion criterion or has not been administered IMP as defined by the protocol.

Eligible subjects who meet all inclusion/exclusion criteria but are unable to participate in the study due to scheduling conflicts/timing will not be considered screening failures.

9.6.2.1.2 Re-screening of Subjects

Subjects who fail to meet all inclusion/exclusion criteria will not be permitted to be re-screened. Screening failures will not be enrolled into the study or receive IMP and cannot be re-screened for the study at any point.

Eligible subjects who meet all inclusion/exclusion criteria but cannot participate in the study due to scheduling conflicts/timing may be rescreened based on investigator's discretion and Sponsor's approval should their availability to participate fall outside the screening window. In these cases, a new screening number must be assigned for each subject to be re-screened and a new informed consent form signed to confirm consent for study participation.

9.6.2.2 Study Examinations

Assessments are to be performed according to the schedule shown in Table 1 *Study Schedules* (page 13) and Table 2 *Detailed Time and Events* (page 16) and depend on time-point of IMP administration.

9.6.2.2.1 Safety

Safety will be evaluated by collecting reported adverse events at regular intervals throughout the study and by the assessment of physical examination findings, vital signs, clinical laboratory parameters and adverse events.

9.6.2.2.2 Medical and Medication History

A complete medical and medication history as well as demographic information will be assessed at the time points indicated in Table 1 *Study Schedules* (page 13).

The medical history will be reviewed and recorded, including:

Medical and Medication History

- Recent ingestion of medication (30 days prior to entering the screening period)
- History of respiratory, cardiovascular, renal, gastrointestinal, hepatic, endocrine, hematological, neurological, psychiatric, musculoskeletal and other diseases.

Demographic information

- Year of birth
- Sex

9.6.2.2.3 Physical Examination

A complete physical examination will be performed at the time-points described in Table 1 *Study Schedules* (page 13).

The physical examination will include a review of the following body systems:

- General appearance
- Skin
- Head, eyes, ears, nose and throat
- Spine/Neck/Thyroid
- Musculoskeletal
- Respiratory
- Cardiovascular
- Neurological
- Abdomen (including liver and kidneys).

Any abnormalities or changes in intensity from the screening visit noted during the review of body systems have to be documented in the source documents. Clinically significant abnormal findings discovered during a physical examination after screening will be documented either as part of medical history (subject forgot to mention an intermittent medical condition at screening) or documented as an Adverse Event or part of an Adverse Event (up from the time after dose administration), if the discovered symptom is leading to a diagnosis.

9.6.2.2.4 Electrocardiogram

A 12-lead ECG will be done at the time points described in Table 1 *Study Schedules* (page 13). Actual ECG assessment times will be documented.

Subjects must be resting in a supine position for at least 5 minutes prior to collecting the ECG. At a minimum, the date and time of when the event was performed, the investigator's assessment and the heart rate, RR, PR, QT, and QRS intervals are to be collected. All clinically significant abnormalities will be recorded on the appropriate source documents.

9.6.2.2.5 Vital Signs

Blood pressure should be determined by cuff (using the same method and in the same position throughout the study). Measurements of vital signs (systolic and diastolic blood pressure as well as pulse rate) will be performed after the subject has been in a sitting position for at least 5 minutes at the time-points specified in Table 1 *Study Schedules* (page 13). Actual vital sign assessment times will be recorded.

All measurements of vital signs must be recorded in the appropriate source documents.

9.6.2.2.6 Temperature

Temperature will be measured using a digital thermometer (orally at the time points specified in Table 1 *Study Schedules* (page 13)).

9.6.2.2.7 Height and Weight

Measurements of height and weight will be performed according to the schedule in *Table 1 Study Schedules* (page 13).

Height is measured in centimeters (cm) and weight is measured in kilograms (kg). Measurements are to be taken in light clothing and socks (without shoes) with pockets emptied. The subject's height is recorded to the nearest cm and weight is recorded to the nearest 0.1 kg.

Height and weight will be measured and used to calculate the BMI using the following formula:

$$\text{BMI} = \frac{weight\,[kg]}{(height\,[m])^2}$$

9.6.2.2.8 Clinical Laboratory Evaluations

All laboratory assays will be performed according to the laboratory's normal procedures. Reference ranges will be supplied by the laboratory and used to assess the laboratory data for clinical significance and out-of-range pathological changes. The physician should assess out-of-range laboratory values for clinical significance, indicating if the value(s) is/are not clinically significant (NCS) or clinically significant (CS). Abnormal laboratory values that are unexpected or not explained by the subject's clinical condition may be, at the discretion of the physician, Investigator or Sponsor, repeated until confirmed, explained, or resolved as soon as possible.

The following laboratory assessments will be performed:

9.6.2.2.8.1 Clinical Chemistry

Blood samples for serum biochemistry will be collected at the time points described in Table 1 *Study Schedules* (page 13). The following parameters will be assessed:

Sodium	Aspartate transaminase (AST)
Potassium	Alanine transaminase (ALT)
Calcium	Alkaline phosphatase (ALP)
Urea	Gamma glutamyl transferase (GGT)

Creatine kinase (total)	Total bilirubin
Albumin	Glucose
Total protein	Pancreas specific amylase
Creatinine	Lactate dehydrogenase (LDH)
Creatinine clearance (MDRD) (only at Screening)	C reactive protein (CRP)

9.6.2.2.8.2 Hematology

A sample of blood will be drawn at the time points described in Table 1 *Study Schedules* (page 13). The following parameters will be assessed:

Hemoglobin	Mean corpuscular hemoglobin concentration (MCHC)
Hematocrit	White blood cell (WBC) count; total and differential
Red blood cells (RBC)	Neutrophils
Mean corpuscular volume (MCV)	Lymphocytes
	Monocytes
Platelet count	Eosinophils
Mean corpuscular hemoglobin (MCH)	Basophils

9.6.2.2.8.3 Serum Pregnancy Test

A sample of blood will be drawn at the time points described in Table 1 *Study Schedules* (page 13). The following parameters will be assessed:

ß-HCG

9.6.2.2.8.4 Urine Pregnancy Test

In all female participants a urine beta-HCG test will be performed on fresh midstream urine at the time points described in Table 1 *Study Schedules* (page 13).

9.6.2.2.8.5 Safety Urinalysis

The following parameters will be analyzed in fresh midstream urine at the time points described in Table 1 *Study Schedules* (page 13).

pH	Ketones
Specific gravity	Bilirubin
Protein	Blood
Glucose	

Microscopic examination will be conducted if blood is detected during urinalysis. The microscopic examination will comprise of RBC, WBC, casts, and bacteria.

9.6.2.2.8.6 Serology

During the screening period only, blood samples will be drawn to test for the presence of HIV, HBsAg, and HCV antibody at the time points described in Table 1 *Study Schedules* (page 13).

HIV testing (HIV I and HIV II)	HCV antibody screen
HBV testing (HBsAg)	

9.6.2.2.8.7 PCR for SARS-CoV-2

For PCR for SARS-CoV-2 samples are taken from nose or nose and throat or from throat depending on the available test at the time points described in Table 1 *Study Schedules* (page 13).

9.6.2.2.8.8 Rapid Antigen Test

For rapid antigen test for SARS-CoV-2 samples are taken from nose or nose and throat or from throat depending on the available test at the time points described in Table 1 *Study Schedules* (page 13).

9.6.2.2.9 Drug and Alcohol Screen

An alcohol test and urine screen for drugs of abuse will be performed at the time points described in Table 1 *Study Schedules* (page 13). Additional drug and alcohol screens may be performed at the physician's discretion.

Urine samples are to be tested for the following:

Methadone (MTD)	Barbiturates
Benzodiazepines	Tricyclic Antidepressants (TCA)
Cocaine	Morphine
Amphetamine/Methamphetamine (MET)	Tetrahydrocannabinol (THC)
Ecstasy	

9.6.2.2.10 Alternative measures with respect to SARS-CoV-2

In case trial participants may not be able to come to the site for protocol-specified visits (e.g., due to the public health emergency related to COVID-19), safety assessments may be performed using alternative methods (e.g., phone contact, virtual visit, alternative location for assessment). This is in accordance with EMA's "Guidance on the Management of Clinical Trials during the COVID-19 (Coronavirus) pandemic Version 2 (27/03/2020)", which states that "Where a trial participant is unable to attend the site, other measures, such as home nursing, if possible given social distancing needs, or contact via phone or telemedicine means, may be required to identify adverse events and ensure continuous medical care and oversight". There will likely be further updates to this guidance, and the sponsor will take these into consideration for decision-making during the course of the trial.
All efforts should be made to collect blood samples for safety and humoral immune response assessment via a home visit within the protocol-specified time window.

Any subject with clinical suspicion of SARS-CoV-2 infection will undergo appropriate testing and referral within the local healthcare system as appropriate.
The WHO/European Centre for Disease Prevention and Control case definition for a suspected case requiring diagnostic testing will be used. This definition may change during the course of the trial:

1. A patient with acute respiratory tract infection (sudden onset of at least one of the following: cough, fever, shortness of breath) AND with no other etiology that fully explains the clinical presentation AND with a history of travel or residence in a country/area reporting local or community transmission during the 14 days prior to symptom onset;

OR

2. A patient with any acute respiratory illness AND having been in close contact with a confirmed or probable COVID-19 case in the last 14 days prior to onset of symptoms;

OR

3. A patient with severe acute respiratory infection (fever and at least one sign/symptom of respiratory disease (e.g. cough, fever, shortness breath)) (SARI) AND requiring hospitalization AND with no other etiology that fully explains the clinical presentation.

Additional testing may be required during the course of the clinical trial at discretion of the investigator and according to local requirement.

9.6.2.2.11 Reactogenicity

To assess reactogenicity after each immunization body temperature and reaction are recorded once daily for at least 7 days after vaccination or longer in case of systemic adverse events until they have subsided. Fever is defined as at least one measured body temperature of ≥38 °C. If fever is detected,

temperature should be measured twice a day (in the morning and evening) until two consecutive measurements are <38 °C. According to the defined time frames in the study schedules, recording might be shortened to less days. During the ambulatory visits at the study ward temperature and solicited injection site and systemic reactions will be assessed and recorded by a designated member of the study team. After dismissal subjects are asked to maintain a diary to record daily temperature and injection site and systemic reactions for 7 days after each dosing and to record medical events for 28 days after each dosing. Subjects will be trained on the thermometer and diary use. The diary might be an electronic diary or a paper diary.
The following items will be recorded:

- Daily temperature (incl. the method by which it was taken)
- Daily measurement of intensity grade of all other injection site and systemic reactions
- Action taken for each recorded event (none, medication taken, physician consulted, hospitalization)

9.6.2.2.12 Local Reaction Assessments

A local reaction is defined as any morphological or physiological change at or near the reaction site, not resulting from a generalized systemic reaction, i.e. urticaria or other distinct clinical entities, i.e. lymphadenopathy. Local reactions include, but are not limited to: pain, erythema, hematoma maculopapular eruptions, induration, pruritus, blistering, ulceration, cellulitis, phlebitis, necrosis or abscesses.

Local reactions, occurring after administration of the IMP, will be graded using the toxicity grading scale in section 9.6.2.2.13.2.7 *Regulatory Agency, Independent Ethics Committee, and Investigative Site Reporting* (page 57).

9.6.2.2.13 Adverse and Serious Adverse Events Assessments

Subjects will be questioned in a general way to ascertain if AEs have occurred (e.g. "Have you had any health problems since the last time you came to the clinic/since you were last questioned?"). This open, standardized questioning should be done discretely in order to prevent subjects from influencing each other. Spontaneous reports of AEs will also be recorded as well as AEs that are observed by an investigator or a staff member.

All AEs will be reviewed, confirmed, and classified by a qualified, designated investigator.

9.6.2.2.13.1 Definition of Adverse Events, Period of Observation, Recording of Adverse Events

An ***Adverse Event*** **(AE)** is any untoward medical occurrence in a clinical investigation subject administered an IMP and that does not necessarily have a causal relationship with this treatment. An AE can therefore be any unfavorable and unintended sign (including an abnormal laboratory finding), symptom, disease or exacerbation of a pre-existing condition temporally associated with the use of a medicinal (test) product, whether or not considered related to the medicinal product (ICH Guidance E2A 1995).

In this clinical trial AEs will be collected from the time of first administration of the IMP until the last follow-up visit, regardless of the relationship to the investigational medicinal product. Following these specifications all AEs in this clinical trial fulfill the WHO's definition of an ***Adverse Event Following Immunization*** (AEFI): An ***AEFI*** is any untoward medical occurrence which follows immunization and which does not necessarily have a causal relationship with the usage of the vaccine. The adverse event may be any unfavorable or unintended sign, abnormal laboratory finding, symptom or disease

Thus, the terms AE and AEFI are interchangeable. For better readability the common term AE will be used.

All AEs will be recorded on the appropriate source documents and subsequently will be entered into the AE module of the eCRF. Where possible, a diagnosis rather than a list of symptoms should be recorded. If a diagnosis has not been made each symptom should be entered as a separate AE.

CAVE: Solicited AEs have to be documented separately as described below. Those must not be summarized in a diagnosis.

If an event fulfills the criteria for an AE but occurs after a study subject signed the informed consent but before the administration of the first vaccination it will be documented in the medical history. If the event occurs after the first immunization the event fulfills the definition AE. An AE will also be documented in case of a worsening of severity of a pre-dose event from the medical history.

All AEs have to be recorded until the last trial day according to the clinical trial protocol. If the investigator becomes aware of a serious AE considered related to the investigational medicinal product, it has to be reported in the safety data base.

A **solicited AE** is a predetermined event occurring within 7 days after application of IMP, which may reflect safety concerns related to the investigational product. The solicited AEs for this study only include:

- Local reaction (swelling, redness/ erythema, itching, warmth, induration, hematoma or pain at site of injection)
- Fever
- Chills
- Myalgia (described to the subject as generalized muscle aches)
- Arthralgia (described to the subject as generalized joint aches)
- Fatigue
- Headache
- Gastrointestinal symptoms (nausea, vomiting, abdominal pain, loose stool and/or diarrhea)
- Malaise

Any of the following events will be classified as an adverse event of special interest (AESI) (according to SPEAC, 14-OCT-2019):

Body System	Relevant to Vaccine Safety in General	Relevant to MERS Clinical Disease/Complications
Neurologic	• Generalized convulsion<br>• Myelitis<br>• Aseptic meningitis<br>• GBS	• Encephalitis<br>• Encephalopathy<br>• ADEM<br>• CNS vasculopathy (stroke)
Hematologic	• Thrombocytopenia	• Disseminated intravascular coagulation
Immunologic	• Anaphylaxis<br>• Vasculitides*	• Enhanced disease if exposed to MERS-CoV after vaccination<br>• Pulmonary eosinophilia/possible immune complexes
Pregnancy<br>Foetus		• Maternal death<br>• Stillbirth<br>• Preterm birth
Respiratory		• ARDS<br>• Pneumonia/pneumonitis
Renal		• Acute renal dysfunction/failure

* Systemic lupus erythematosus (SLE), Henoch Schonlein purpura (HSP), single-organ cutaneous vasculitis, vasculitic peripheral neuropathy

For AESIs with severe conditions the diagnosis certainty as defined according to the Brighton Collaboration [8] should be captured in the safety database.

Reporting procedure for AESIs are the same as for SAEs (section 9.6.2.2.13.2 *Serious Adverse Event (SAE) Procedures*, page 55).

All AEs must be followed to closure (i.e. the subject's health has returned to his/her baseline status or all variables have returned to normal), an outcome is reached, stabilization (the investigator does not expect any further improvement or worsening of the event), or the event is otherwise explained regardless of whether the subject is still participating in the clinical trial and clinical judgment indicates that

further follow-up is not warranted. When appropriate, medical tests and examinations are performed so that resolution of event(s) can be documented.

9.6.2.2.13.1.1 Severity Categorization

The severity of AEs must be recorded. If the severity of an AE/SAE changes, or if there are any other changes then these must be made within the same AE record. Also, only the highest severity needs to be recorded. Worsening of pre-treatment events from the medical history, after initiation of IMP, must be recorded as new AEs.

The medical assessment of severity is determined by using the following definitions:

Mild: A type of AE that is usually transient and may require only minimal treatment or therapeutic intervention. The event does not generally interfere with usual activities of daily living.

Moderate: A type of AE that is usually alleviated with additional specific therapeutic intervention. The event interferes with usual activities of daily living, causing discomfort but poses no significant or permanent risk of harm to the research subject.

Severe: A type of AE that interrupts usual activities of daily living, or significantly affects clinical status, or may require intensive therapeutic intervention.

The term “severe” is here used to describe the severity/intensity of the specific event; it is not the same as “serious”, which is based on subject/event outcome or action criteria.

In addition to the severity assessment with the categories mild, moderate and severe, for the AEs described below, the severity will be assessed according to the following scales (grade 0 to 4 or 5).

Toxicity Grading Scale for Local Adverse Events

Table 3 Toxicity grading scale for local adverse events

Local Reaction to Injectable Product	Mild (Grade 1)	Moderate (Grade 2)	Severe (Grade 3)	Potentially Life Threatening (Grade 4)
Pain	Does not interfere with activity	Repeated use of non-narcotic pain reliever > 24 hours or interferes with activity	Any use of narcotic pain reliever or prevents daily activity	Emergency room (ER) visit or hospitalization
Tenderness	Mild discomfort to touch	Discomfort with movement	Significant discomfort at rest	ER visit or hospitalization
Erythema/Redness*	2.5 – 5 cm	5.1 – 10 cm	> 10 cm	Necrosis or exfoliative dermatitis
Induration/Swelling**	2.5 – 5 cm and does not interfere with activity	5.1 – 10 cm or interferes with activity	> 10 cm or prevents daily activity	Necrosis

*In addition to grading the measured local reaction at the greatest single diameter, the measurement should be recorded as a continuous variable.
**Induration/swelling should be evaluated and graded using the functional scale as well as the actual measurement.

https://www.fda.gov/regulatory-information/search-fda-guidance-documents/toxicity-grading-scale-healthy-adult-and-adolescent-volunteers-enrolled-preventive-vaccine-clinical accessed on 06MAY2021

Toxicity Grading Scale for Physical Observations

Table 4 Toxicity grading scale for physical observations

Observation	Grade 1	Grade 2	Grade 3	Grade 4	Grade 5
Fever (orally)	38.0 °C - 39.0 °C	> 39.0 °C - 40.0 °C	> 40.0 °C for ≤ 24 hours	> 40.0 °C for ≥ 24 hours	Death

Observation	Grade 1	Grade 2	Grade 3	Grade 4	Grade 5
Sinus tachycardia (bpm)*	Asymptomatic, intervention not indicated	Symptomatic, non-urgent medical intervention indicated	Urgent medical intervention indicated	-	-
Sinus bradycardia (bpm)**	Asymptomatic, intervention not indicated	Symptomatic, intervention not indicated; change in; change in medication initiated	Symptomatic, intervention indicated	Life-threatening consequences, urgent intervention indicated	Death
Hypertension	**Adult:** Systolic BP 120 - 139 mm Hg or diastolic BP 80 - 89 mm Hg	**Adult:** Systolic BP 140 - 159 mmHg or diastolic BP 90 - 99 mmHg if previously WNL; change in baseline medical intervention indicated; recurrent or persistent (≥24 hrs); symptomatic increase by >20 mmHg (diastolic) or to >140/90 mmHg; monotherapy indicated initiated	**Adult:** Systolic BP ≥160 mm Hg or diastolic BP ≥100 mm Hg; medical intervention indicated; more than one drug or more intensive therapy than previously used indicated	**Adult and Pediatric:** Life-threatening consequences (e.g. malignant hypertension, transient or permanent neurologic deficit, hypertensive crisis); urgent intervention indicated	Death
Hypotension	Asymptomatic, intervention not indicated	Non-urgent medical intervention indicated	Medical intervention indicated; hospitalization indicated	Life-threatening consequences and urgent intervention indicated	Death

Definitions	
Fever	A disorder characterized by elevation of the body's temperature above the upper limit of normal.
Tachycardia	A disorder characterized by a dysrhythmia with a heart rate greater than 100 beats per minute that originates in the sinus node.
Bradycardia	A disorder characterized by a dysrhythmia with a heart rate less than 60 beats per minute that originates in the sinus node.
Hypertension	A disorder characterized by a pathological increase in blood pressure.
Hypotension	A disorder characterized by a blood pressure that is below the normal expected for an individual in a given environment.

Adapted from the Common Terminology Criteria for Adverse Events (CTCAE), Version 5.0 Published: November 27, 2017: https://ctep.cancer.gov/protocolDevelopment/electronic_applications/docs/CTCAE_v5_Quick_Reference_5x7.pdf accessed on 06MAY2021

Toxicity grading scale for systemic AEs excluding the physical observations listed above

Table 5 Toxicity grading scale for systemic AEs

Systemic sign/symptom	Mild (Grade 1)	Moderate (Grade 2)	Severe (Grade 3)	Potentially Life Threatening (Grade 4)
Headache	No interference with activity	Repeated use of non-narcotic pain reliever > 24 hours or	Significant; any use of narcotic pain reliever or prevents daily activity	ER visit or hospitalization

		some interference with activity		
Fatigue	No interference with activity	Some interference with activity	Significant; prevents daily activity	ER visit or hospitalization
Myalgia	No interference with activity	Some interference with activity	Significant; prevents daily activity	ER visit or hospitalization
Nausea/vomitting	No interference with activity or 1 – 2 episodes/24 hours	Some interference with activity or > 2 episodes/24 hours	Prevents daily activity, requires outpatient IV hydration	ER visit or hospitalization for hypotensive shock
Diarrhea	2 – 3 loose stools or < 400 gms/24 hours	4 – 5 stools or 400 – 800 gms/24 hours	6 or more watery stools or > 800gms/24 hours or requires outpatient IV hydration	ER visit or hospitalization

**Systemic Illness**	**Mild (Grade 1)**	**Moderate (Grade 2)**	**Severe (Grade 3)**	**Potentially Life Threatening (Grade 4)**
Illness or clinical adverse event (as defined according to applicable regulations)	No interference with activity	Some interference with activity not requiring medical intervention	Prevents daily activity and requires medical intervention	ER visit or hospitalization

https://www.fda.gov/regulatory-information/search-fda-guidance-documents/toxicity-grading-scale-healthy-adult-and-adolescent-volunteers-enrolled-preventive-vaccine-clinical accessed on 06MAY2021

**Systemic sign/symptom**	**Mild (Grade 1)**	**Moderate (Grade 2)**	**Severe (Grade 3)**	**Potentially Life Threatening (Grade 4)**
Arthralgia	No interference with activity	Some interference with activity	Significant; prevents daily activity	ER visit or hospitalization
Chills	No interference with activity	Some interference with activity	Significant; prevents daily activity	ER visit or hospitalization
Abdominal Pain	No interference with activity	Some interference with activity not required medical intervention	Prevents daily activity and requires medical intervention	ER visit or hospitalization

Analogous to the FDA's 2007 voluntary guidance.

9.6.2.2.13.1.2 Relationship Categorization

An investigator assesses each AE for its relationship to the IMP.

The assessment of the relationship of an AE to the administration of IMP is a clinical decision based on all available information at the time of and after the occurrence of the event. The factors which may be considered when evaluating the relationship of an AE to the IMP include: time from exposure to investigational medicinal product until onset of the event; recovery or improvement on discontinuation of investigational medicinal product; availability of alternative explanations such as underlying or intercurrent diseases; concomitant medications or treatments; pharmacology and pharmacokinetic of the investigational medicinal products; known response pattern for this class of drug; recurrence on reintroduction of the investigational medicinal product.

If there is no valid reason for suggesting a relationship, then the AE should be classified as 'not related'. Otherwise, if there is any valid reason, even if undetermined or untested, for suspecting a possible cause-and-effect relationship between the investigational medicinal product and the occurrence of the AE, then the AE should be considered 'related'. The causality must be documented in the source document.

The following additional guidance may be helpful:

Term	Relationship	Definition
Related	Yes	The temporal relationship between the event and the administration of the investigational medicinal product is compelling and/or follows a known or suspected response pattern to that product, and the event cannot be explained by the subject's medical condition, other therapies, or accident.
Not Related	No	The event can be readily explained by other factors such as the subject's underlying medical condition, concomitant therapy, or accident and no plausible temporal or biologic relationship exists between the investigational medicinal product and the event.

Upon request of the Sponsor the following additional categorization according to the WHO-CIOMS classification for causality assessment may be necessary:

If the physician decides there is a causal relationship, he/she should assess the relationship of an AE to the IMP according to the following categories:

**Levels**	**Definition**
**Very likely/ certain**	Clinical event with a **proximal, biologically plausible, time** relationship to vaccine administration and which **cannot be explained** by concurrent disease or other drugs or chemicals
**Probable**	Clinical event with a **relatively brief interval between vaccination and the event** and which **unlikely to be attributed** to concurrent disease or other drugs or chemicals
**Possible**	Clinical event with a **relatively brief interval between vaccination and the event** but which **could also be explained** by concurrent disease or other drugs or chemicals
**Unlikely**	Clinical event with a **time** relationship to vaccine administration that makes causal connection **improbable** but which **could plausibly be explained** by underlying disease or other drugs or chemicals
**Unrelated**	Clinical event with a biologically **incompatible time** relationship to vaccine administration and which **could be explained** by underlying disease or other drugs or chemicals
**Unclassifiable**	Clinical event with **insufficient information** to permit assessment and identification of the cause

9.6.2.2.13.1.3 Outcome Categorization

The outcome of AEs must be recorded during the course of the study in the eCRF. Outcomes are as follows:

- Resolved/resolved
- Recovering/resolving
- Resolved with sequelae
- Not recovered/not resolved/ ongoing
- Fatal

- Unknown

9.6.2.2.13.1.4 Clinical Laboratory Evaluations

A change in the value of a safety laboratory investigation can represent an AE if the change is clinically relevant or if, during treatment with the IMP, a shift of a parameter is observed from a normal value to an abnormal value, or a further worsening of an already abnormal value. When evaluating such changes, the extent of deviation from the reference range, the duration until return to the reference range, either while continuing treatment or after the end of treatment with the IMP, and the range of variation of the respective parameter within its reference range, must be taken into consideration.

If, at the end of the treatment phase, there are abnormal laboratory values which were not present at baseline, further clinical or laboratory investigations should be performed until the values return to within reference range or until a plausible explanation (e.g. concomitant disease) is found for the pathological laboratory values.

The investigator should decide, based on the above criteria and the clinical condition of a subject, whether a change in a laboratory parameter is clinically significant and therefore represents an AE.

9.6.2.2.13.1.5 Procedures in Case of Pregnancy

All pregnancies that occur during the trial in female trial subjects as well as female partners of male trial subjects should be followed up. Although pregnancy is not an SAE, information about any pregnancy should be reported promptly to CTC North Safety Department on the same timeline as an SAE (without undue delay but not later than within 24 hours of the first awareness of the event) using the pregnancy form filed in the investigator site file. In case the pregnant woman gives her informed consent regarding the data collection, monitoring of the pregnant subject or female partner shall continue until the conclusion of the pregnancy. Study vaccination will be discontinued for the pregnant subject. Should pregnancy result in a congenital abnormality, birth defect, miscarriage, or medically indicated abortion an SAE must be submitted to Sponsor using the SAE reporting procedures described in section 9.6.2.2.13.2.1 *Reporting Procedures* (page 55).

9.6.2.2.13.1.6 Abuse, Misuse, Overdose, and Medication Error

Abuse, misuse, overdose, or medication error (as defined below) must be reported to the Sponsor according to the SAE reporting procedure whether or not they result in an AE/SAE.

Misuse - Intentional or unintentional use of a study medication other than as directed or indicated at any dose, which is at or below the dose defined for overdose. (Note: this includes a situation where the study medication is not used as directed at the dose prescribed by the protocol.)

Overdose - Intentional or unintentional intake of a dose of study medication higher than the protocol prescribed dose for each subject.

Medication Error - A mistake made in prescribing, dispensing, administration, and/or use of the study medication. For studies, medication errors are reportable only as defined below.

Administration of an expired product should be considered as a reportable medication error.

Cases of subjects missing doses of product are not considered reportable as medication errors.

9.6.2.2.13.2 Serious Adverse Event (SAE) Procedures

9.6.2.2.13.2.1 Reporting Procedures

All initial SAE reports must be reported by the investigator to the CTC North Safety Department without undue delay but not later than within 24 hours of the first awareness of the event. All SAE follow-up reports must be reported in a timely manner. The investigator must complete, sign, and date the Serious Adverse Event Form provided in the eCRF and verify the accuracy of the information recorded on the form with the corresponding source documents (Note: source documents are not to be sent unless requested) and send the form via the eCRF form to the Sponsor's responsible Person for Pharmacovigilance:

Name:	CTC North Safety Department
Fax number:	[redacted]

Phone number:	[redacted]
Email:	[redacted]

CTC North forwards all SAEs for review to the medical monitor of the Sponsor. The medical monitor assesses SAEs causality and expectedness and returns the SAE documentation to the CTC North Safety Department.

All AESIs must be reported in the same way as SAEs.

Additional diagnostics for AESIs and SAEs only need to be captured in the safety database, not in the eCRF.

In case an SAE is considered related to the study medication (i.e. a SUSAR), the CTC North Safety Department informs the medical monitor and the Coordinating Investigator. On behalf of the Sponsor CTC North has to inform the competent authorities, the ethics committees and the PIs about the SUSAR according to the applicable law and within the appropriate timelines.

For the Netherlands, the Sponsor or delegated CRO will report the SAEs through the web portal *ToetsingOnline* to the accredited METC that approved the protocol, within 7 days of first knowledge for SAEs that result in death or are life threatening followed by a period of maximum of 8 days to complete the initial preliminary report. All other SAEs will be reported within a period of maximum 15 days after the Sponsor has first knowledge of the serious adverse events.

9.6.2.2.13.2.2 Serious Adverse Event (SAE) Definition

A Serious Adverse Event (SAE) is any untoward medical occurrence (whether considered to be related to IMP or not) that follows immunization and at any dose:

- Results in death
- Is life-threatening
- Requires inpatient hospitalization or prolongation of existing hospitalization
- Results in persistent or significant disability/incapacity
- Is a congenital abnormality/birth defect
- Is an Important Medical Event, i.e. an event that may jeopardize the subject and may require medical or surgical intervention to prevent one of the outcomes listed in this definition

Hospitalizations which are the result of elective or previously scheduled surgery for pre-existing conditions which have not worsened after initiation of treatment should not be classed as SAEs. For example, an admission for a previously scheduled ventral hernia repair would not be classed as a SAE.

However, complication(s) resulting from a hospitalization for an elective or previously scheduled surgery that meets serious criteria must be reported as a SAE(s).

9.6.2.2.13.2.3 SAE Onset and Resolution Dates

The onset date of the SAE is defined as the date the event meets serious criteria. SAE stop date is defined as the date the event no longer meets serious criteria. The resolution date is the date the symptoms are resolved or resolved with sequelae/event is no longer present. In the case of hospitalizations, the hospital admission and discharge dates are considered the onset and resolution dates, respectively.

9.6.2.2.13.2.4 Fatal Outcome

Any SAE that results in the subject's death (i.e. the SAE was noted as the primary cause of death) should have fatal checked as an outcome and the resolution date of death recorded as the resolution date. For all other events ongoing at time of death that did not contribute to the subject's death, the outcome should be considered not resolved, without a resolution date recorded.

For any SAEs that results in the subject's death or any ongoing events at the time of death, the action taken with the IMP should be recorded as "dose not changed" or "not applicable" (if the subject never received IMP).

9.6.2.2.13.2.5 Serious Adverse Reaction (SAR)

An AE (expected or unexpected) that is both serious and, in the opinion of the reporting investigator or Sponsors, believed to be possibly, probably or definitely due to an IMP or any other study treatments, based on the information provided.

9.6.2.2.13.2.6 Suspected unexpected serious adverse reaction (SUSAR)

Adverse events are SUSARs if the following three conditions are met:

- the event must be serious;
- there must be a certain degree of probability that the event is a harmful and an undesirable reaction to the medicinal product under investigation, regardless of the administered dose;
- the adverse reaction must be unexpected, that is to say, the nature and severity of the adverse reaction are not in agreement with the product information as recorded in investigator's Brochure for an unauthorized medicinal product.

9.6.2.2.13.2.7 Regulatory Agency, Independent Ethics Committee, and Investigative Site Reporting

The Sponsor is responsible for SUSAR reporting to the relevant Regulatory Authorities/European Union (EU) central Independent Ethics Committees and Investigators participating in the clinical trial.

The expedited reporting will occur not later than 15 days after the Sponsor has first knowledge of the adverse reactions. For fatal or life threatening cases the term will be maximal 7 days for a preliminary report with another 8 days for completion of the report.

In addition to the expedited reporting of SUSARs, the Sponsor will submit, once a year throughout the clinical trial, a safety report (DSUR) to the responsible authorities and Ethics Committees.

The Netherlands:

The Sponsor will report expedited the following SUSARs to the METC:

SUSARs that have arisen in the clinical trial that was assessed by the METC;

SUSARs that have arisen in other clinical trials of the same sponsor and with the same medicinal product, and that could have consequences for the safety of the subjects involved in the clinical trial that was assessed by the METC.

The remaining SUSARs are recorded in an overview list (line-listing) that will be submitted once every half year to the METC. This line-listing provides an overview of all SUSARs from the study medicine, accompanied by a brief report highlighting the main points of concern.

The expedited reporting of SUSARs through the web portal Eudravigilance or local reporting portals is sufficient as notification to the competent authority.

The Sponsor will report expedited all SUSARs to the competent authorities in other Member States, according to the requirements of the Member States.

9.6.3 Immunology Measurements

Detailed information on blood sampling and sample processing are defined in the Laboratory Manual.

9.6.3.1 Immunogenicity Assays

The information on the blood volume drawn for immunogenicity assays given below refers to maximum amounts. Details on the exact blood volume are specified in the Laboratory Manual.

9.6.3.1.1 Enzyme-Linked Immunosorbent Assay (ELISA)

To identify binding MVA-MERS-S antibody responses induced by the candidate vaccine, ELISA assays will be performed on serum samples drawn at the time points described in Table 1 *Study Schedules* (page 13). The analysis will be performed at the Philipps University Marburg, Germany and the EMC in Rotterdam, Netherlands, according to the processes defined in the Laboratory Manual. The cut-off values used to define seroconversion is assay-dependent and is specified in the Laboratory Manual.

9.6.3.1.2 Neutralization Assay

Neutralizing antibody responses against MVA will be measured using the same serum sample as used for ELISA (see Table 1 *Study Schedules* (page 13)). The analysis will be performed at the Philipps

University Marburg, Germany and at the EMC in Rotterdam, Netherlands. Details are described in the Laboratory Manual. The Philipps University Marburg will perform a VNT assay; and EMC the PRNT80 assay.

9.6.3.1.3 T-cell Responses

The magnitude and kinetics of MVA- as well as MERS-S specific T-cell responses will be assessed by IFN-γ ELISpot and flow cytometry assays. The assays will be performed on cryopreserved PBMC (EDTA tubes) obtained at the time points described in Table 1 *Study Schedules* (page 13). Blood samples for T-cell immunogenicity analysis will be drawn by direct venipuncture into four EDTA tubes, capped and mixed by inversion (x3). The actual time that the sample was obtained will be recorded on the source documents. After applying a tourniquet, venous blood will be taken with a disposable needle. The magnitude and kinetics of T-cell responses will be assessed by IFN-γ ELISpot and flow cytometry assays. The analysis will be performed by the UKE Group Emerging Infections, located at BNITM, Hamburg, Germany, according to the processes defined in the Laboratory Manual.

9.6.3.1.4 Innate immunity

Innate immune responses will be evaluated to understand early immune signatures induced by the vaccine candidate with potential to predict subsequent adaptive immunogenicity.

First, in the exploratory translational research program, gene expression analysis from whole blood (2.5 mL PAXgene tube) will be performed at baseline and early time points post-vaccination as described in Table 1 *Study Schedules* (page 13) to investigate early innate immunity gene signatures induced by the vaccine candidate and their impact on adaptive B- and T- cell immunity. Analyses will be performed by the UKE Group Emerging Infections, located at BNITM, Hamburg, Germany in cooperation with the Group from Andre Franke, located in Kiel.

Innate immune cells will be investigated with special focus on activation status. For this, PBMC will be analyzed using flow cytometry.

Lastly, plasma obtained by centrifugation of EDTA-blood tubes will be analyzed via Luminex Multiplex Assays. Flow cytometry and Luminex analyses will be performed by the UKE Group Emerging Infections, located at BNITM, Hamburg, Germany.

9.6.3.2 Shipment of Samples

Samples from the study site CTC North will be transported to the UKE Group Emerging Infections, located at BNITM, Hamburg, Germany.

After processing and aliquoting sera, samples will be stored at ≤ -20 °C. Part of frozen serum samples will be shipped to Marburg, Rotterdam and Munich to assess the presence or induction of MVA, MERS-S or coronavirus antibodies. Analyses will be performed at the Philipps-University Marburg (Prof. Stephan Becker), at the EMC (Dr. Haagmans) and at the Institute for Infectious Diseases and Zoonoses at LMU (Prof. Sutter):

Prof. Stephan Becker
Institute for Virology
Philipps University Marburg
Hans Meerweinstr. 2
35043 Marburg, Germany

Dr. Bart Haagmans
Erasmus Medical Centre
Ee building 17th floor
Dr Molewaterplein 50
3000 CA Rotterdam, The Netherlands

Prof. Dr. Gerd Sutter
Institute for Infectious Diseases and Zoonoses
Ludwig-Maximilians-Universität München
Veterinärstr. 13
80539 Munich, Germany

Samples from the study site EMC will be transported to the Research Group of B. Haagmans located at EMC.

After processing and aliquoting sera, samples will be frozen and stored at ≤ -20 °C. Part of frozen serum samples will be shipped from EMC to the Philipps University Marburg (Prof. Stephan Becker) and to the Institute for Infectious Diseases and Zoonoses at LMU (Prof. Sutter).

After processing of PBMC and freezing at ≤ -150 °C, samples will be shipped to the UKE Group Emerging Infections (Prof. Marylyn Addo), located at BNITM, Hamburg. In addition, PAXGene tubes will be shipped to the Laboratory in Hamburg.

Prof. Dr. Marylyn M. Addo
Department of Clinical Immunology of Infectious Diseases
Bernhard-Nocht-Institute
Bernhard Nocht Str. 74
20357 Hamburg, Germany

Part of the PAXgene samples from both trial sites received by the BNITM will be investigated in cooperation with the Institute of Clinical Molecular Biology in Kiel (Prof. Franke).

Prof. Dr. Andre Franke
Institute of Clinical Molecular Biology (IKMB)
Kiel University, Christian-Albrechts-Universität zu Kiel (CAU)
Rosalind-Franklin-Str. 12
24105 Kiel, Germany

9.6.3.3 Future Use of Stored Samples

Left over blood samples may be used in the further evaluation of an adverse event or for the subsequent evaluation of additional parameters that are identified as important to the evaluation of an individual subject or to the study, or for further research regarding the immune response to the MVA-MERS vaccine. Any unused part of the blood samples will be securely stored at Sponsor in Germany, Hamburg for 15 years for analyses of exploratory objectives. Parts of the pseudonymized blood samples may be shipped to collaborators for further evaluation.

9.6.4 Safety Variables

Assessment of safety is performed for the safety collective. Safety data include:

- Adverse events (including changes from baseline in physical examination findings)
- Clinical laboratory results
- Vital signs
- Physical examination

The safety evaluation will be based upon the review of the individual values (potentially clinically important abnormalities) and descriptive statistics (summary tables, graphics).

9.6.4.1 Adverse Events

The adverse events will be listed per subject using MedDRA terminology (lower level term, preferred term and system organ class) and will be reported in tables summarizing the frequency of subjects with adverse events and adverse events by treatment and body system, the number of adverse events and subjects with adverse events by treatments and the characteristics of adverse events.

For the hematology, clinical laboratory and the urine analysis deviations from the reference ranges will be summarized in frequency tables.

9.6.4.2 Clinical Laboratory

All relevant clinical laboratory variables obtained during screening, final examination or the clinical trial periods will be reported in appropriate tables together with descriptive statistics. Clinical laboratory findings outside of the reference range will be flagged.

9.6.4.3 Vital Signs

For blood pressure and pulse rate descriptive statistics will be listed by sampling times (screening and follow-up) according to the data captured in the eCRF.

9.6.4.4 ECG

The results (normal or abnormal, and clinical significance) of the 12- lead ECG will be listed by sampling times (screening and End of study) according to the data captured in the eCRF.

9.7 Data Quality Assurance

9.7.1 Quality Assurance System

Protocol development, case report form and trial master file, investigator site file, content of subject information and consent, application for ethics approval, data processing, central and on-site monitoring, and evaluation will follow the Standard Operating Procedures (SOP) of the CTC North, CR2O and CR2O subcontractors.

The study may be subject to audits by the QAU of the Sponsor, the CTC North, CR2O or the study site.

9.7.2 Monitoring

During the study, the monitor/clinical research associate will visit the investigational site regularly to check the completeness of subject records, the accuracy of entries in the eCRF, the adherence to the protocol and to GCP, the progress of enrollment, and to ensure that study drug is being stored, dispensed, and accounted for according to specifications. The monitor/clinical research associate is capable of reading the local language as well as being fluent in English. Key trial personnel must be available to assist the monitor during these visits. The investigator must maintain source documents for each subject in the study, consisting of case and visit notes (hospital or clinic medical records) containing demographic and medical information, laboratory data and the results of any other tests or assessments. All information on eCRFs must be traceable to these source documents in the subject's file. Data not requiring a written or electronic record will be defined before study start and will be recorded directly in the eCRFs, which will be documented as being the source data. The investigator must also keep the original of the signed informed consent form. The investigator must give the monitor access to all relevant source documents to confirm their consistency with the eCRF entries. Monitoring standards require full verification for the presence of informed consent, adherence to the inclusion/exclusion criteria, documentation of SAEs, and the recording of data that will be used for all primary and safety variables.

A combination of remote monitoring and regular on-site visits will be performed depending on the possibility for on-site visits due to the current COVID-19 pandemic. Close-out visits are planned as on-site visits. The frequency and kind of monitoring visits will depend on the study site's recruitment rate and current situation in each of the study site. The detailed extent of the monitoring and the risk based approach considering the COVID-19-pandemic situation and its influence on the daily life and the health system will be defined in the overall monitoring plan applicable for both study sites.

9.7.3 Documentation and Data Collection

Subject medical information obtained by this study is confidential, and disclosure to third parties other than those noted below is prohibited. With the subject's permission, medical information may be given to his personal physician or other appropriate medical personnel responsible for his/her welfare.

The information required by the protocol is entered into an electronic subject source. The electronic subject source is to be considered source data in addition to the automatic print outs as well as paper subject records.

Paper source data templates will be prepared to collect and document at least the data required for transfer into the eCRF. During initial data collection, the study staff enters the information directly into these source data templates. All entries will be completed in blue ball-point pen; they must be clearly legible and signed by the person who made the entry.

Next, site staff will transfer the study data from the source documents into the electronic CRF. All eCRFs will be checked for completeness and electronically signed by an investigator in order to ensure data entry accuracy.

If a subject is a screening failure (according to section 9.6.2.1.1 *Screening Failure*, page 44), only baseline characteristic data of this subject may be entered in the eCRF and will not be analyzed in the CSR.

Corrections to source data documents will be dated and initialed. Reasons for the corrections should be given. Corrections to eCRF entries must be electronically signed and reasons for the corrections must be provided. The date on which the correction was performed is automatically recorded by the system's audit trail.

A study monitor will review the eCRF data for completeness and accuracy during the monitoring visits (source data verification (SDV)). The study monitor will point out any discrepancies between source data and the data captured in the eCRF. The monitor will issue electronic queries to site staff to initiate

discrepancy resolution. Discrepancies which require eCRF data corrections have to be resolved by authorized site personnel by answering these monitoring queries. Discrepancies which results in a correction of data have to be resolved by authorized site personnel.

Data generated by this study must be available for inspection by representatives of other national and local health authorities, the Sponsor, and the for each study site, if appropriate.

Subjects will be identified on CRFs and other documents submitted to the Sponsor or organizations working on behalf of the Sponsor only by subject number, not by name or initials. Documents not to be submitted to the Sponsor or organizations working on behalf of the Sponsor that identify the subject (e.g., the signed informed consent) must be maintained in confidence at the study site.

9.7.4 Data Management

Data management will double check all eCRF entries as defined in the data management plan (DMP). Quality control and data validation procedures such as programmed automatic edit and consistency checks ensure data validity and accuracy immediately at the point of entry into the clinical database. The database application which is used to capture electronic study data is fully CFR part 11 (Code of Federal Regulations) compliant. Thus, it is access restricted, demands electronic signatures, maintains an electronic audit trail and provides appropriate backup functionalities. Details of the application and eCRF configuration and all further data management procedures will be described in the DMP.

The database will only be locked after all queries and discrepancies that may occur during data entry are resolved.

Upon request safety reports and interim analysis will be generated and provided to the respective members of the LSB/DSMB.

After database lock, the data in the study data base will be exported and SAS datasets will be compiled for statistical analysis. The data will be exported in SAS transport files or other SAS-compatible format and transferred electronically to the responsible biometrician for statistical analysis. The locked SAS database will be used to generate the subject listings, tabulations, and analyses.

9.7.5 Archival of documents

The study sites will maintain the trial documents and take measures to prevent accidental or premature destruction of these documents according to the respective SOPs.

All documents related to the study will be retained by the Sponsor until at least 15 years after the end of the study.

The Sponsor will receive an electronic copy of the eCRF for archival.

If documents shall be retained for a longer period, it is the responsibility of the Sponsor to inform the study sites when these documents no longer need to be retained.

In case of any change concerning the archiving modalities the study sites have to inform the Sponsor immediately.

9.8 Statistical Methods Planned in the Protocol and Determination of Sample Size

9.8.1 Statistical and Analytical Plan

Details for the statistical evaluation of the results will be given in a separate statistical analysis plan (SAP).

9.8.1.1 Software to be used

The data will be analyzed using SAS 9.4 or later by the Sponsor and/or designated CRO. Any analysis done independently by the investigator should be submitted to the Sponsor before publication or presentation.

9.8.1.2 Analysis Populations

Eligibility of subjects will be determined within the data review meeting (DRM). The following populations will be investigated.

- Safety population: All subjects who received at least one vaccination will be part of the safety population.

- Per protocol population: All subjects who received at least two injections will be considered the per protocol population.

All safety endpoints will be investigated in the safety population. Immune response endpoints will be investigated in the per protocol population only.

9.8.1.3 Statistical analyses

The primary objective of the study will be the assessment of safety, and in particular reactogenicity in the 7 days following injection.

All safety information will be listed by subject and by treatment group.

Data will be summarized with respect to demographic and baseline characteristics, immune response, and safety data. Safety data comprises AEs, laboratory tests, vital signs, and physical examination. AE data will be reported tabulated by system organ class and preferred term using MedDRA coding.

Comparability across dose-levels as well as compared to placebo will be evaluated descriptively. Furthermore, the subgroup sex will be assessed.

Exposure to study medication will be summarized by number of injections and dose intensity using descriptive statistics. Reasons for not giving all protocol required injections will be presented.

Efficacy will be determined by immune response to vaccination as expressed by activation of immune cells in peripheral blood.

For continuous variables, the mean, standard deviation (SD), coefficient of variation (CV), minimum, maximum and median will be reported per treatment group. For parameters that appear to be log-normally distributed, the geometric mean (GeoM) and the geometric coefficient of variation (GeoCV) may be reported.

Unless otherwise specified, baseline is defined as the time-point closest but prior to the first administration of the vaccine.

Categorical variables will be summarized by treatment group in frequency tables (number and percentages).

Number of related AEs between groups will be compared using descriptive statistics. For the total frequency of adverse events by treatment group 95% Miettinen-Nurminen confidence intervals will be presented. Furthermore, humoral response based on number of responders, mean, and median of ELISA and virus neutralization assays will be compared between groups.

If deemed necessary, 95% confidence intervals will be provided for means (metric variables) and percentages (categorical variables).

Further details regarding the analyses will be provided in the SAP.

9.8.2 Determination of Sample Size

The sample size for part B was determined by practical considerations and is not based on a statistical power calculation. However, differences between groups can be detected with the following assumptions. Assuming there are 135 subjects in the per protocol population (112 evaluable subjects and 23 drop outs assuming a dropout rate of 20%). One dose cohort with the same verum vaccination time points consists of 30 subjects (25 evaluable and 5 dropouts), and placebo consists of 15 subjects (12 evaluable and 3 dropouts). Under the assumption that 8 of 25 subjects (32%) have at least one AE, and assuming 0 of 12 subjects have at least one AE, the 95% confidence interval would be significantly different.

If considering all subjects as one group without considering the different verum vaccination time points, one dose cohort consists of 60 subjects (50 evaluable and 10 dropouts) and placebo consists of 15 subjects (12 evaluable and 3 dropouts). Under the assumption that 10 of 50 subjects (20%) have at least one AE and assuming 0 of 25 subjects have one AE, the 95% confidence interval would be significantly different between the two groups.

These values are deemed sufficient to detect a significant health risk from the vaccination without exposing too many subjects to the investigational product.

When all subjects completed 3 months (study day 56 plus 28 days) in the study, one interim analysis is planned to determine optimal dose and injection scheme for future studies.

9.9 Changes in the conduct of the study or planned analysis

Modifications of the protocol are permitted only if they are authorized by the Sponsor and the Coordinating Investigator in writing.

Deviations and changes to the study protocol will be classified by the Sponsor and the study center as:

Note-to-File: This refers to clarifications which are not considered changes of the protocol.

Study protocol amendment: This refers to changes of the protocol. If they fulfill the criteria as set out in applicable law for definition as substantial amendment they need to be approved by the Ethics Committee, the Competent Authority or both. Changes to the study protocol may also induce revision of the subject information sheet/informed consent form. Accordingly, subjects undergoing trial assessment procedures at the time of implementation of the change have to be given the amended version and have to be asked for consent to continue on this amended trial.

A ‘substantial amendment’ is defined as an amendment to the terms of the applicable regulations.

All substantial amendments will be notified to the relevant authorities.

9.10 Safety Boards

9.10.1 LSB

During Part A of the trial, the LSB will review and judge the safety and tolerability.

For the decision about dose escalation in Part A, the Local Safety Board (LSB) will review safety data (AEs, vital signs, and laboratory safety data) obtained until day 7 after the 1st immunization of all subjects of the low dose cohort.

For the decision about entering Part B, the LSB will review safety data obtained until day 7 after the 1st immunization of all subjects of the high dose cohort. After the 1st dosing of the low dose cohort the LSB evaluation will take the results of the pre-clinical studies and ongoing phase I studies into account. Rules for decision will be layed out in a charter.

In addition, an LSB meeting will be conducted whenever safety relevant data occur that might have an influence on the trial.

Members of the LSB will be the medical monitor, as well as the coordinating investigator, or at least one of the deputies and a representative of the Sponsor.

At a minimum the holding rules (criteria for termination of the study) specified in the protocol will automatically apply (if activated) for not proceeding with a higher dose.

The decisions of the LSB meeting will be briefly summarized and signed by all members involved in the meeting and the decision. The summarized safety report of each LSB Meeting will be submitted to the whole DSMB for notification. Relevant findings will be reported to the responsible authorities.

9.10.2 DSMB

During Part B of the trial, the DSMB will review and judge the safety and tolerability.

Evaluation will take the results of the pre-clinical studies and the ongoing studies in prior mentioned other countries into account. Rules for decision will be laid down in a charter.

The advice(s) of the DSMB will be sent to the Sponsor of the study. Relevant findings will be reported to the responsible authorities.

10 REPORTS

All reports to the Sponsor will be in English. The Sponsor will receive the original CSR.

The CSR is the property of the Sponsor. Publication of the report or of part of it may only be allowed when authorized by the Sponsor in consultation with the study center.

10.1 Clinical Study Report

All clinical, analytical and statistical results will be presented in a CSR. The outline of this report will accord to the ICH-GCP E3 document "Structure and Content of Clinical Study Reports" of July 17, 1996. Within a year upon completion of the clinical trial, a summary of the CSR will be sent to the Ethics Committees and Competent Authorities as required by appropriated law.

10.2 Additional Reports

Upon completion of the study, a short report will be sent to the ethics committees, stating any undesired event and indicating whether study objectives have been attained. Short reports to the authorities after study termination will be provided as required by law. The short report on the risks to human health or the environment resulting from the IMP will be prepared after the main part of the clinical trial (EOS on D 252) is completed.

The Sponsor or a designee will submit a summary of the progress of the trial to the accredited METC once a year. Information will be provided on the date of inclusion of the first subject, numbers of subjects included and numbers of subjects that have completed the trial, serious adverse events/ serious adverse reactions, other problems, and amendments.

11 EXTENDED FOLLOW-UP PHASE PART B FOR EXPLORATORY OBJECTIVES

11.1 Study Schedule

Table 6: Part B Extended follow-up

Phase of study[6]	Ambulatory Visits		
**Study Visit (v)**	**v15**	**v16**	**v17**
**Study day**	**v12+168 (+84)**	**v12+336 (+84)**	**v12+672 (±84)**
**Study Months**	**15**	**21**	**33**
Informed Consent[1]	√		
I/E Criteria	√		
PCR test SARS-CoV-2	------------------- continuously[5] --------------------		
Rapid Antigen Test SARS-CoV-2	------------------- continuously[5] --------------------		
Targeted illnesses[2]	√	√	√
Serious adverse events (SAEs) & adverse events of special interest (AESIs)[7]	√	√	√
Targeted Concomitant Medication[3]	√	√	√
Targeted exposure questioning[4]	√	√	√
Humoral responses	√	√	√
PBMC freezing and plasma aliquots	√	√	√

[1] Signed informed consent form needs to be available latest at visit 15 before performing any study related assessments. The consent might be obtained at an earlier visit as well.

[2] COVID-19 infection since the last visit, febrile illness within 4 weeks before each study visit

[3] All COVID-19 vaccinations and pox vaccinations or other MVA (incl. MVA-vectored) vaccinations since last study visit, other vaccinations within 4 weeks before each study visit, receipt of chronic (defined as more than 14 days) immune suppressants or other immune-modifying drugs (for corticosteroids, this will mean prednisone, or equivalent, greater than or equal to 0.5 mg/kg/day, intranasal, inhaled and topical steroids are not considered immune suppressant treatment)

[4] Stays in one of the following countries since the last visit:

- Saudi Arabia
- United Arab Emirates
- Jordan
- Qatar
- Kuwait
- Iran
- Oman
- Kenya

[5] SARS-CoV-2 testing can be performed throughout the study when clinically indicated and at the discretion of the investigator, or according to local requirements.

[6] Participation in the extended follow-up phase for exploratory objectives is optional. The decision is made by the site-specific PI.

[7] SAEs according to section 9.6.2.2.13.2.2 *Serious Adverse Event (SAE) Definition*:

Any untoward medical occurrence (whether considered to be related to IMP or not) that follows immunization and at any dose:

- results in death
- is life threatening
- requires inpatient hospitalization or prolongation of existing hospitalization
- results in persistent or significant disability/incapacity
- is a congenital abnormality/birth defect
- is an Important Medical Event, i.e. an event that may jeopardize the subject and may require medical or surgical intervention to prevent one of the outcomes listed in this definition

AESI from the list in 9.6.2.2.13.1 *Definition of Adverse Events, Period of Observation, Recording of Adverse Events*

- Generalized convulsion

- Myelitis
- Aseptic meningitis
- GBSThrombocytopenia
- Anaphylaxis
- Vasculitides (Systemic lupus erythematosus (SLE), Henoch Schonlein purpura (HSP), single-organ cutaneous vasculitis, vasculitic peripheral neuropathy
- Encephalitis
- Encephalopathy
- ADEM
- CNS vasculopathy (stroke)
- Disseminated intravascular coagulation
- Enhanced disease if exposed to MERS-CoV after vaccination
- Pulmonary eosinophilia/possible immune complexes
- Maternal death
- Stillbirth
- Preterm birth
- ARDS
- Pneumonia/pneumonitis
- Acute renal dysfunction/failure

11.2 Subject Information and Consent

Consent for the extended follow-up phase of the clinical trial will be obtained separately.

11.3 Study Objectives

11.3.1 Exploratory Objectives

- To evaluate MERS-CoV-S-specific antibody responses in healthy male and female study subjects until the end of the extended follow-up phase.
- To evaluate MERS-CoV-S-specific cellular immune responses, including MERS-CoV-S-induced B- and T-cell memory responses
- To evaluate long-term humoral and cellular immunity against the viral vector MVA and other relevant orthopoxviruses
- To investigate long-term vaccine-induced non-neutralizing humoral immune responses and antibody functions
- To assess sex differences in the immunity to vaccination

11.3.2 Exploratory Endpoints

- Immunogenicity:
  - Humoral immunity: Magnitude of MERS-CoV-S-specific antibody responses (ELISA and neutralization assays) monitored in approved laboratories
- Magnitude of long-term cellular immune responses after administration of MVA-MERS-S_DF-1
- Magnitude of long-term MVA-MERS-S-specific CD4+ and CD8+ T-cell responses by ELISpot and intracellular cytokine staining using flow cytometry
- Magnitude of long-term non-neutralizing antibody responses against MVA-MERS-S
- Magnitude of long-term humoral and cellular responses to the MVA vector

11.4 Investigational Plan

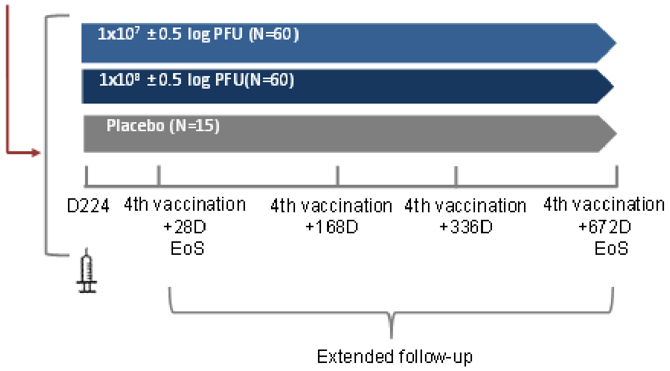

Figure 3 Study Design extended follow-up phase part B

11.4.1 Inclusion Criteria

1) Written informed consent for extended follow-up phase.
2) Enrollment in interventional part B of the clinical trial.
3) At least 2 vaccinations in the interventional part B and being no drop-out.

11.4.2 Exclusion Criteria

None.

It is explicitly possible to perform the extended follow-up visits in subjects that participate in any kind of a clinical study. There is no interference with samples or reactions expected as the extended follow-up of this study only includes blood samples for immunogenicity analyses and documentation of targeted

information to support the interpretation of results. The total amount of blood collected from a volunteer will not exceed 300 mL over 24 months. In case subjects participate in another clinical study, the extended follow-ups can be performed upon approval of the other study's sponsor.

11.4.3 Treatments

There is no treatment in this extended follow-up.

11.4.3.1 Extended Follow-up visits

Subjects from part B who had an end of study visit on v12 + 28 days (+7) and who agree to participate in the extended follow-up phase return to the study site 6 months (+84 days), 12 months (+84 days), and 24 months (+84 days) after the 4th vaccination for additional follow-up visits. Participation in the extended follow-up phase for exploratory objectives is optional. The decision is made by the site-specific PI. The following events will be performed:

- Informed consent for extended follow-up. The signed form needs to be available latest at visit 15 before performing any study related assessments. The consent might be obtained at an earlier visit as well.
- Inclusion/exclusion criteria at visit 15
- Targeted illnesses:
  - Covid-19 infection since the last visit
  - Febrile illness within 4 weeks before each study visit
- SAEs according to section 9.6.2.2.13.2.2 Serious Adverse Event (SAE) Definition:
  - Any untoward medical occurrence (whether considered to be related to IMP or not) that follows immunization and at any dose:
    - results in death
    - is life threatening
    - requires inpatient hospitalization or prolongation of existing hospitalization
    - results in persistent or significant disability/incapacity
    - is a congenital abnormality/birth defect
    - is an Important Medical Event, i.e. an event that may jeopardize the subject and may require medical or surgical intervention to prevent one of the outcomes listed in this definition
  - AESI from the list in 9.6.2.2.13.1 Definition of Adverse Events, Period of Observation, Recording of Adverse Events

Any of the following events will be classified as an adverse event of special interest (AESI) (according to SPEAC, 14-OCT-2019):

Body System	Relevant to Vaccine Safety in General	Relevant to MERS Clinical Disease/Complications
Neurologic	• Generalized convulsion<br>• Myelitis<br>• Aseptic meningitis<br>• GBS	• Encephalitis<br>• Encephalopathy<br>• ADEM<br>• CNS vasculopathy (stroke)
Hematologic	• Thrombocytopenia	• Disseminated intravascular coagulation
Immunologic	• Anaphylaxis<br>• Vasculitides*	• Enhanced disease if exposed to MERS-CoV after vaccination<br>• Pulmonary eosinophilia/possible immune complexes
Pregnancy<br>Foetus		• Maternal death<br>• Stillbirth<br>• Preterm birth

Respiratory		• ARDS<br>• Pneumonia/pneumonitis
Renal		• Acute renal dysfunction/failure

* Systemic lupus erythematosus (SLE), Henoch Schonlein purpura (HSP), single-organ cutaneous vasculitis, vasculitic peripheral neuropathy

- Targeted Concomitant Medication:
  - Covid-19 vaccinations and pox vaccinations or other MVA (incl. MVA-vectored) vaccinations since the last study visit
  - Other vaccinations within 4 weeks before each study visit
  - Receipt of chronic (defined as more than 14 days) immune suppressants or other immune-modifying drugs
    - For corticosteroids, this will mean prednisone, or equivalent, greater than or equal to 0.5 mg/kg/day
    - Intranasal, inhaled and topical steroids are not considered immune suppressant treatment
- Targeted exposure questioning: Stays in one of the following countries since the last visit:
  - Saudi Arabia
  - United Arab Emirates
  - Jordan
  - Qatar
  - Kuwait
  - Iran
  - Oman
  - Kenya
- Humoral responses
- PBMC freezing and plasma aliquots
- PCR and rapid antigen test for SARS-CoV-2 may be performed throughout the study at discretion of the Investigator and according to local requirements

11.4.3.2 Early Termination

A subject that ended participation in the extended follow-up phase early should be asked to inform the study site about SAEs and AESIs that occurred since the last study visit.

11.4.3.3 Adverse and Serious Adverse Events Assessments

In the extended follow-up phase only COVID-19 infections since the last visit and febrile illnesses within 4 weeks before each study visit as well as SAEs and AESIs are documented.

11.4.4 Immunology Measurements

Immunological methods will be continued as described in 9.6.3 except the analysis of the innate immune system 9.6.3.1.4 which will not be performed in the extended study.

11.5 Statistical and Analytical Plan

Descriptive statistical analysis will be performed to investigate the durability of vaccine-induced immune responses.

11.6 Clinical Study Report

The clinical study report for the interventional part will be finalized although the extended follow-up is still ongoing. The results of the extended follow-up are exploratory objectives inly and therefor will not be presented the clinical study report.

12 REFERENCES

1 Zaki AM, van Boheemen S, Bestebroer TM, Osterhaus AD, Fouchier RA. Isolation of a novel coronavirus from a man with pneumonia in Saudi Arabia. N Engl J Med. 2012 Nov 8; 367(19): 1814-20.

2 http://www.who.int/emergencies/mers-cov/en/, accessed on 15-JUL-2020, 15:18

3 Memish ZA, Zumla AI, Al-Hakeem RF, Al-Rabeeah AA, Stephens GM. Family cluster of Middle East respiratory syndrome coronavirus infections. N Engl J Med. 2013 Jun 27; 368(26):2487-94.

4 Raj VS, Smits SL, Provacia LB, van den Brand JM, Wiersma L, Ouwendijk WJ, Bestebroer TM, Spronken MI, van Amerongen G, Rottier PJ, Fouchier RA, Bosch BJ, Osterhaus AD, Haagmans BL. Adenosine deaminase acts as a natural antagonist for dipeptidyl peptidase 4-mediated entry of the Middle East respiratory syndrome coronavirus. J Virol. 2014 Feb; 88(3):1834-8.

5 Costello DA, Millet JK, Hsia CY, Whittaker GR, Daniel S. Single particle assay of coronavirus membrane fusion with proteinaceous receptor-embedded supported bilayers. Biomaterials. 2013 Oct; 34(32):7895-904.

6 Chan PK, Chan MC. Tracing the SARS-coronavirus. J Thorac Dis. 2013 Aug; 5 (Suppl 2):S118-21.

7 Sutter G, Moss B. Nonreplicating vaccinia vector efficiently expresses recombinant genes. Proc Natl Acad Sci U S A. 1992 Nov 15; 89(22):10847-51.

8 https://www.brightoncollaboration.org/case-definitions, accessed 15-JUL-2020, 15:20
