# Supplementary material for: Two-year persistence of MERS-CoV-specific antibody and T cell responses after MVA-MERS-S vaccination in healthy adults
Source: Nat Commun. 2026 Jan 9;17:480. doi: 10.1038/s41467-025-68248-5 (PMC12800105; doi:10.1038/s41467-025-68248-5)
Supplement: Supplementary file 3 — Reporting Summary [file 41467_2025_68248_MOESM3_ESM.pdf]

nature portfolio

Corresponding author(s): Dr. Leonie Mayer

Last updated by author(s): Sep 23, 2025

Reporting Summary

Nature Portfolio wishes to improve the reproducibility of the work that we publish. This form provides structure for consistency and transparency in reporting. For further information on Nature Portfolio policies, see our Editorial Policies and the Editorial Policy Checklist.

Statistics

For all statistical analyses, confirm that the following items are present in the figure legend, table legend, main text, or Methods section.

n/a	Confirmed	
☐	☒	The exact sample size (*n*) for each experimental group/condition, given as a discrete number and unit of measurement
☐	☒	A statement on whether measurements were taken from distinct samples or whether the same sample was measured repeatedly
☐	☒	The statistical test(s) used AND whether they are one- or two-sided<br>*Only common tests should be described solely by name; describe more complex techniques in the Methods section.*
☒	☐	A description of all covariates tested
☐	☒	A description of any assumptions or corrections, such as tests of normality and adjustment for multiple comparisons
☐	☒	A full description of the statistical parameters including central tendency (e.g. means) or other basic estimates (e.g. regression coefficient) AND variation (e.g. standard deviation) or associated estimates of uncertainty (e.g. confidence intervals)
☐	☒	For null hypothesis testing, the test statistic (e.g. F, t, r) with confidence intervals, effect sizes, degrees of freedom and P value noted<br>*Give P values as exact values whenever suitable.*
☒	☐	For Bayesian analysis, information on the choice of priors and Markov chain Monte Carlo settings
☒	☐	For hierarchical and complex designs, identification of the appropriate level for tests and full reporting of outcomes
☐	☒	Estimates of effect sizes (e.g. Cohen's *d*, Pearson's *r*), indicating how they were calculated

Our web collection on statistics for biologists contains articles on many of the points above.

Software and code

Policy information about availability of computer code

Data collection	Excel v1808 (build 10403.20013, Microsoft), electronic case report forms (eCRF): secuTrial data base (version 5.1.0.20), Harmony image software (version 4.9, Perkin Elmer)
Data analysis	GraphPad Prism v9.5.1 (build 733, Dotmatics), Excel v1808 (build 10403.20013, Microsoft)

For manuscripts utilizing custom algorithms or software that are central to the research but not yet described in published literature, software must be made available to editors and reviewers. We strongly encourage code deposition in a community repository (e.g. GitHub). See the Nature Portfolio guidelines for submitting code & software for further information.

Data

Policy information about availability of data

All manuscripts must include a data availability statement. This statement should provide the following information, where applicable:

- Accession codes, unique identifiers, or web links for publicly available datasets
- A description of any restrictions on data availability
- For clinical datasets or third party data, please ensure that the statement adheres to our policy

The de-identified, individual-level immunogenicity data generated in this study have been deposited in the ZFDM repository database of the University of Hamburg under accession code 17971 [http://doi.org/10.25592/uhhfdm.17971] and are provided in the Supplementary Information/Source Data file. De-identified, individual-level demographic and clinical data are not publicly available due to data privacy laws. Access for non-commercial purposes can be requested by

researchers from the corresponding author (l.mayer@uke.de) and will be granted upon signing of a data transfer agreement. An initial response to data access requests will be given within four weeks. The original clinical study protocol is available in the supplementary information. Source data are provided with this paper.

Research involving human participants, their data, or biological material

Policy information about studies with human participants or human data. See also policy information about sex, gender (identity/presentation), and sexual orientation and race, ethnicity and racism.

Reporting on sex and gender	Biological sex was self-reported by study participants (see Table 1 and Table S1 for demographics stratified by treatment group). Antibody data was not stratified by sex in our study due to small sample sizes of individual treatment groups. As pre-specified by the study protocol of this randomized, double-blind trial, men and women were randomly assigned to treatment groups.
Reporting on race, ethnicity, or other socially relevant groupings	We did not report on race, ethnicity, or other socially relevant groupings in this study.
Population characteristics	Only healthy adults (aged 18-55 years) were included in this phase 1 vaccine trial. Immunogenicity data are reported only for participants who had received all three MVA-MERS-S vaccine doses according to the study protocol.
Recruitment	Volunteers were recruited through public advertisement.
Ethics oversight	The trial protocol was reviewed and approved by the relevant authorities in Germany (Paul Ehrlich Institute) and the Netherlands (Central Committee on Research Involving Human Subjects) and by the ethics committees of the Hamburg medical association and Erasmus Medical Center. The protocol amendment to conduct the two-year follow-up of the Hamburg study cohort was approved by the ethics committee of the Hamburg medical association.

Note that full information on the approval of the study protocol must also be provided in the manuscript.

Field-specific reporting

Please select the one below that is the best fit for your research. If you are not sure, read the appropriate sections before making your selection.

☒ Life sciences ☐ Behavioural & social sciences ☐ Ecological, evolutionary & environmental sciences

For a reference copy of the document with all sections, see nature.com/documents/nr-reporting-summary-flat.pdf

Life sciences study design

All studies must disclose on these points even when the disclosure is negative.

Sample size	No power calculations were done for the phase 1b clinical trial. 139 participants were enrolled (a sample size of 135 participants was planned), which resulted in nominally sized treatment groups for a phase 1 trial. As the two-year follow-up was only conducted at the Hamburg study site, the possible sample size was restricted to 74. Of those, 55 participants re-consented to the two-year follow-up and were included in the analyses of this article.
Data exclusions	Immunogenicity data of participants who had not received all three MVA-MERS-S vaccine doses according to study protocol were excluded from the analyses. These data can be provided upon request.
Replication	The WHO international standard (1st international standard for anti-MERS-CoV antibody NIBSC Code: 19/178) was measured alongside the nFRNT, S1 IgG ELISA and VNT antibody assays and used to normalize the results of the S1 IgG ELISA and the nFRNT assay. Samples were measured in triplicates in the VNT assay and a human monoclonal antibody served as a neutralisation control. All three antibody assays have been published previously and showed strong inter-assay correlations in MERS-CoV-vaccinated sera (Raadsen et al., 2025, Lancet Infectious Diseases). All attempts at replication were successful.
Randomization	Computer randomisation was used to assign participants to one of five groups, with an allocation ratio of 2:2:2:2:1. An unmasked team ran the randomisation procedure via an electronic data capture system (SecuTrial; Interactive Systems, Berlin, Germany) during the first vaccination visit and prepared syringes of MVA-MERS-S or placebo for administration by masked staff.
Blinding	The sponsor, clinical and laboratory staff, and the participants were masked to the treatment allocation. Syringes were wrapped in semitranslucent tape to obscure slight differences in aspects between the two doses of the vaccine and placebo. To mask participants to allocation to the 28-day or 56-day intervals, placebo doses were administered to the treatment groups on whichever day they did not receive their second dose of MVA-MERS-S. Unmasked staff (ie, nurses conducting the randomisation and preparing the syringes, the pharmacist, and pharmacy staff) did not do any activities related to the participants and were not involved in outcome assessment. Effective masking was substantiated by study monitors. An in-person monitoring visit was done during vaccination of the first individual that was dosed at both sites, to check if blinding procedures were followed correctly.

Reporting for specific materials, systems and methods

We require information from authors about some types of materials, experimental systems and methods used in many studies. Here, indicate whether each material, system or method listed is relevant to your study. If you are not sure if a list item applies to your research, read the appropriate section before selecting a response.

Materials & experimental systems

n/a	Involved in the study
☐	☒ Antibodies
☐	☒ Eukaryotic cell lines
☒	☐ Palaeontology and archaeology
☒	☐ Animals and other organisms
☐	☒ Clinical data
☒	☐ Dual use research of concern
☒	☐ Plants

Methods

n/a	Involved in the study
☒	☐ ChIP-seq
☒	☐ Flow cytometry
☒	☐ MRI-based neuroimaging

Antibodies

Antibodies used	HRP-labelled rabbit anti-human IgG (polyclonal, Dako Agilent), anti-VSV-G neutralising antibody (clone 8G5F11, Absolute Antibody), human anti-MERS spike monoclonal antibody (clone m336, Detai Bio-Tech Co.)
Validation	anti-VSV-G neutralising antibody (clone 8G5F11, Absolute Antibody):<br>- recombinant monoclonal antibody to VSV-G, manufactured using Ab's Recombinant Platform with variable regions (i.e. specificity) from the hybridoma 8G5F11.<br><br>human anti-MERS spike monoclonal antibody (clone m336, Detai Bio-Tech Co.):<br>- a human recombinant monoclonal antibody that detects of Spike glycoprotein of Middle East respiratory syndrome-related coronavirus and is tested for use in ELISA and neutralization assays<br><br>All antibodies were acquired commercially and were validated by the manufacturer.

Eukaryotic cell lines

Policy information about cell lines and Sex and Gender in Research

Cell line source(s)	Human embryonic kidney cell line (HEK-293T), human lung adenocarcinoma cell line (Calu-3), Human hepatocellular carconima cell line (Huh7)
Authentication	The cell lines were not further authenticated after purchase.
Mycoplasma contamination	All cell lines tested negative for mycoplasma contamination.
Commonly misidentified lines<br>(See ICLAC register)	None.

Clinical data

Policy information about clinical studies

All manuscripts should comply with the ICMJE guidelines for publication of clinical research and a completed CONSORT checklist must be included with all submissions.

Clinical trial registration	NCT04119440
Study protocol	The full trial protocol can be found in the supplementary information of this article.
Data collection	Recruitment for the phase 1b clinical trial started in April 2021. Study visits including data collection took place between July 2021 and November 2022 at the Rotterdam and Hamburg study sites. The study protocol was amended to allow for an extended follow-up of study participants of the Hamburg study site. This two-year follow-up was conducted between December 2022 and February 2025.
Outcomes	The primary objective of this trial was safety and tolerability. The secondary objective was the evaluation of MERS-CoV-specific antibody responses, as a measure of vaccine immunogenicity. These objectives were pre-specified in the study protocol. The extended follow-up was only subject to exploratory objectives (as specified in the study protocol). These exploratory objectives included the evaluation of MERS-CoV-specific antibody responses to assess the long-term durability of the vaccine-induced immune response.

Plants

Seed stocks	*Report on the source of all seed stocks or other plant material used. If applicable, state the seed stock centre and catalogue number. If plant specimens were collected from the field, describe the collection location, date and sampling procedures.*
Novel plant genotypes	*Describe the methods by which all novel plant genotypes were produced. This includes those generated by transgenic approaches, gene editing, chemical/radiation-based mutagenesis and hybridization. For transgenic lines, describe the transformation method, the number of independent lines analyzed and the generation upon which experiments were performed. For gene-edited lines, describe the editor used, the endogenous sequence targeted for editing, the targeting guide RNA sequence (if applicable) and how the editor was applied.*
Authentication	*Describe any authentication procedures for each seed stock used or novel genotype generated. Describe any experiments used to assess the effect of a mutation and, where applicable, how potential secondary effects (e.g. second site T-DNA insertions, mosiacism, off-target gene editing) were examined.*
